# Volatilomic Study of the Chemodiversity of Metabolites in Wild Macromycetes of the Lower Montane Humid Forest

**DOI:** 10.3390/jof12090630

**Published:** 2026-08-23

**Authors:** Juan Pablo Betancourt Arango, Gonzalo Taborda Ocampo, Yenny Leandra Valencia-Cardona, Sandra Montoya Barreto

**Affiliations:** 1 Research Group in Chromatography and Related Techniques, Department of Chemistry, University of Caldas, Manizales 170004, Colombia; chemquantum@outlook.com (J.P.B.A.); gtaborda@ucaldas.edu.co (G.T.O.); 2 Technological Development Center for Bioprocess and Agroindustry Plant, Department of Engineering, Research Group in Food and Agroindustry, University of Caldas, Manizales 170004, Colombia; leandra.valenciacardona@udecaldas.edu.co

**Keywords:** macromycete fungi bioprospecting, volatile organic compounds, solid-phase microextraction, volatilomics

## Abstract

Colombia’s rich biodiversity highlights the potential for chemical exploration of plants, animals, and fungi. Fungi play essential ecological roles in organic matter degradation, nutrient cycling, and symbiotic interactions. Fungal volatilomics has emerged as a valuable approach for characterizing volatile organic compounds (VOCs) with potential biotechnological, pharmaceutical, and environmental applications. This study aimed to characterize the volatile organic compounds (VOCs) naturally emitted by wild macromycete species collected in the Botanical Garden of the University of Caldas (Manizales, Colombia), contributing to the chemical characterization of their volatilome and providing a basis for future studies on fungal biomarkers and bioactive metabolites. VOCs were extracted using static and dynamic headspace solid-phase microextraction (HS-SPME, SHS-SPME, and DHS-SPME) with PDMS/DVB/CARB fibers and Tenax tubes. Samples were analyzed both in situ and under controlled laboratory conditions using GC–MS for qualitative identification. More than 120 VOCs were identified, including alcohols, ketones, terpenoids, aromatic compounds, and branched alkanes. The volatilomic profiles differed among fungal species and according to the extraction methodology employed, highlighting compounds such as 1-octen-3-ol, 3-octanone, D-limonene, caryophyllene, and 2,6-di-*tert*-butyl-4-methylphenol. Multivariate statistical analyses and metabolic pathway annotation revealed distinct volatilomic patterns and suggested associations with lipid metabolism, fatty acid biosynthesis, and terpenoid biosynthesis. The chemical diversity identified demonstrates that tropical wild macromycetes constitute an important source of structurally diverse volatile metabolites. Furthermore, the complementary use of SHS-SPME, DHS-SPME, and laboratory HS-SPME expanded VOC coverage, providing a comprehensive volatilomic dataset that may support future applications in fungal chemotaxonomy, bioprospecting, ecological studies, and the discovery of candidate bioactive compounds.

## 1. Introduction

Colombia is a country rich in biodiversity, particularly in higher plant and animal species. However, current research has largely focused on flora and fauna, with relatively few studies addressing the chemical exploration and biological potential of fungi [1]. These organisms play a vital role in natural ecological cycles, often in association with other species, and are key contributors to soil formation. Fungi can be classified as macromycetes or micromycetes, with the ability to degrade various types of natural organic materials through parasitic, symbiotic, or saprophytic interactions [2]. Thus, mycological flora represents an indispensable component of global biodiversity. Although fungal diversity has been estimated to comprise several million species worldwide, only a small proportion has been formally described to date, highlighting the substantial taxonomic and biochemical knowledge gaps that remain for this kingdom [1].

Macromycetes are a group of fungal organisms distinguished by the production of visible fruiting bodies, such as mushrooms, brackets, or sclerotia, predominantly belonging to the phyla Basidiomycota and Ascomycota. These fungi play essential ecological roles as saprotrophic decomposers, mycorrhizal symbionts, and occasionally as parasites, contributing significantly to organic matter recycling and the balance of natural ecosystems [3]. Their ability to degrade complex polymers such as lignin, cellulose, and chitin is due to the production of a wide array of specialized extracellular enzymes, including laccases, peroxidases, and cellulases, positioning them as key players in soil formation and nutrient cycling [4,5]. Moreover, macromycetes have attracted considerable scientific interest for their ability to produce bioactive secondary metabolites, including terpenoids, phenolic compounds, polysaccharides, and volatile compounds, which possess antimicrobial, antioxidant, immunomodulatory, and even antitumor properties [6]. These attributes highlight their relevance not only in ecological contexts but also in biotechnology, medicine, and sustainable agriculture. Despite this growing interest, only a relatively small fraction of fungal diversity has been taxonomically and chemically characterized, underscoring the enormous potential of macromycetes as a source of novel natural products and structurally diverse metabolites [7].

Additionally, macromycetes have become a subject of increasing interest in the emerging field of volatilomics, a discipline focused on the study of volatile organic compounds (VOCs) emitted by living organisms [8]. Fungal VOCs (FVOCs) generated by macromycetes represent a unique class of secondary metabolites with potential applications as chemical biomarkers, species identification tools, and bioactive agents in food, pharmaceutical, and environmental industries [9]. These volatile emissions play not only functional roles in fungal ecology (insect attraction or defense against other microorganisms), but also contribute to chemotaxonomic profiling, enabling differentiation of metabolomic patterns among edible, toxic, and environmentally significant fungi. Furthermore, fungal volatilomics has emerged as a complementary strategy for investigating fungal biodiversity because VOC profiles frequently reflect species-specific metabolic characteristics and ecological adaptations.

The volatilome of a fungal fruiting body is strongly influenced by its physiological state and surrounding environmental conditions. Under natural in situ conditions, volatile organic compounds are continuously released as products of primary and secondary metabolism while the fungus remains attached to its original substrate and maintains interactions with neighboring microorganisms, plants, insects, and abiotic environmental factors [10]. Consequently, VOCs collected directly in the field are considered representative of the natural volatilomic state of the organism. In contrast, detachment of fruiting bodies from their natural substrate for laboratory analysis interrupts water and nutrient exchange, modifies oxygen availability and humidity, and exposes tissues to mechanical manipulation during sampling and transport. These changes may disrupt cellular redox homeostasis, promoting the accumulation of reactive oxygen species (ROS) and triggering oxidative stress responses that include lipid peroxidation, activation of antioxidant enzymes, and remodeling of secondary metabolism. Such physiological alterations have been associated with qualitative and quantitative modifications in fungal volatile emissions, particularly in compounds derived from fatty acid oxidation and terpenoid metabolism [11]. Therefore, differences observed between VOC profiles obtained in situ and after laboratory handling may reflect metabolic changes associated with post-harvest physiological stress. However, because oxidative stress was not directly evaluated through biochemical or molecular assays in the present study, these interpretations are based on metabolite annotation and previous experimental evidence reported in the literature and should not be interpreted as direct experimental demonstration.

Various analytical techniques have been employed to identify secondary metabolites in fungi, including high-performance liquid chromatography (HPLC–DAD/MS), gas chromatography–mass spectrometry (GC–MS), and nuclear magnetic resonance (NMR) spectroscopy. These techniques enable the identification of novel fungal species by comparing metabolite profiles in conjunction with molecular phylogeny [12]. Thus, it is crucial to implement strategies aimed at the discovery of novel natural products (NPs) that facilitate differentiation between fungal species. GC–MS analysis, in particular, has proven effective in distinguishing edible, toxic, and potentially toxic species through the identification of amino acids, fatty acids, and sterols via derivatization, as well as a wide range of volatile organic compounds (VOCs) [13]. Fungi generate a diverse array of VOCs, with approximately 250 fungal-derived compounds biochemically synthesized through primary and secondary metabolic pathways. These compounds fulfill multiple functions, including roles in medical diagnostics and ecological signaling, as VOC-mediated interactions are crucial in natural environments [14].

The diversity of FVOCs holds significant biotechnological potential for applications such as biofuel development, mycofumigation, and biocontrol strategies, representing a novel approach for bioprospecting and the discovery of new NPs [14]. Moreover, fungal-bacterial interactions contribute to the generation of a broad spectrum of microbial volatile organic compounds (MVOCs) [15], including FVOCs [16]. The variation in VOCs is influenced by fungal species, developmental stage, and environmental conditions that modulate their production [17]. FVOCs are characterized by different combinations of organic functional groups, producing aromas typically associated with molds, mushrooms, and yeasts. Among these, 1-octen-3-ol (commonly referred to as mushroom alcohol or matsutake alcohol) stands out for its involvement in communication, germination inhibition, plant growth promotion, and semiochemical signaling in arthropod interactions [17]. The identification of FVOCs has been instrumental in developing fungal chemotaxonomy, facilitating the detection of candidate volatile biomarkers to characterize both environmentally beneficial fungi and those hazardous to human health [18].

VOCs are produced by all living microorganisms, which express their biological potential through active interactions within natural biological cycles and cross-kingdom activities involving fungi and other ecosystem organisms [19]. The genetic basis for the biosynthesis of VOCs and the enzymes involved in the assembly of secondary metabolites is currently under investigation [20]. Research on fungal VOC biosynthesis has reported insights into key flavor components, such as sulfur-containing compounds, C_8_ structures, and certain aldehydes, linked to specific functional groups [21]. Nonetheless, the underlying biochemical mechanisms remain an active area of study. Consequently, studies integrating analytical chemistry, fungal taxonomy, and multivariate statistical analyses are essential to expand current knowledge of fungal volatilomes and to generate hypotheses regarding the metabolic origin and ecological relevance of these compounds.

In tropical countries like Colombia, biodiversity is vast due to geographic location, climate, and the abundance of natural resources, among other factors. Within this context, forests and ecosystems located in various regions of the country—such as those found in the department of Caldas—hold great potential for the bioprospecting of the diverse groups of organisms inhabiting these environments, with fungi being among the least studied kingdoms. Furthermore, there is a lack of both basic and applied research on the fungal biota of Colombian forests, particularly concerning taxonomic identification and biotechnological potential [22]. To address this knowledge gap, the present study aimed to characterize the volatile organic compounds (VOCs) emitted by different genera of wild macromycetes under their natural in situ conditions and following laboratory sampling, using complementary HS-SPME and DHS-SPME extraction strategies coupled with GC–MS analysis. In addition, multivariate statistical analyses and metabolic pathway annotation were employed to compare volatilomic profiles among fungal species and extraction methodologies, providing a comprehensive chemical characterization of fungal VOC diversity. The inferred metabolic pathways and ecological implications discussed herein are based on metabolite annotation and literature-supported interpretation and should therefore be considered as hypotheses requiring further biological validation.

## 2. Materials and Methods

### 2.1. Sampling and Sample Preparation

Fungal samples were randomly collected from different areas of the Botanical Garden of the University of Caldas, located in Manizales, Colombia (5°03′25.5″ N, 75°29′27.2″ W; approximately 2150 m above sea level). According to Holdridge’s life zone classification, the study area corresponds to a Lower Montane Wet Forest (LMWF), characterized by annual precipitation exceeding 1800 mm, a mean annual temperature of 17.5 °C, and persistently high relative humidity, providing favorable environmental conditions for the development of diverse macromycete communities. During the sampling campaigns, the soil remained highly moist due to recent rainfall and was covered by a leaf litter layer approximately 3 cm thick, creating an organic substrate that promotes fungal growth and fruiting body development. The sampled fungi were collected from naturally occurring substrates, including decaying wood, leaf litter, and soil-associated organic matter. Specimen collection was conducted under the Framework Permit for Biological Collections granted through Resolution No. 1166 of 9 October 2014, issued by the National Environmental Licensing Authority (ANLA), and its subsequent amendments contained in Resolutions No. 2497 of 2018, No. 854 of 2019, No. 1396 of 2021, No. 519 of 2022, No. 26 of 2024, No. 002547 of 2024, and No. 000680 of 2025.

Volatile organic compounds (VOCs) were extracted both in situ and under laboratory conditions using small portions of each fungal specimen. For the in situ analyses, VOCs were collected immediately after locating the fruiting bodies while they remained attached to their natural substrate, thereby preserving their physiological state and minimizing environmental disturbance. In contrast, laboratory analyses were performed after transporting the specimens from the field, where the interruption of water and nutrient exchange together with sample manipulation could promote post-harvest physiological responses associated with oxidative stress. Consequently, the in situ extractions were considered representative of the naturally emitted fungal volatilome, whereas laboratory extractions were regarded as reflecting VOC profiles potentially influenced by collection-induced physiological changes rather than experimentally induced oxidative stress. Depending on specimen availability and field conditions, individual fungal samples were analyzed using one, two, or all three extraction approaches (SHS-SPME, DHS-SPME, and laboratory HS-SPME), providing complementary volatilomic information for each taxonomically identified specimen. Solid-phase microextraction (SPME) was performed using fibers coated with polydimethylsiloxane/divinylbenzene/carboxen (PDMS/DVB/CARB).

### 2.2. Extraction Procedure of Volatile Organic Compounds (VOCs) in Fungi

#### 2.2.1. Dynamic Headspace Solid-Phase Microextraction (DHS-SPME) for Field VOC Collection

For this DHS method, field extractions were performed for 60 min using a silanized glass tube packed with Tenax TA adsorbent (Sigma-Aldrich, St. Louis, MO, USA) connected to a 9 V portable diaphragm vacuum pump (NMP 03 Series, KNF Neuberger Inc., Trenton, NJ, USA) operating at its maximum flow rate. After sampling, the tube was transported to the laboratory for preparation and subsequent GC–MS analysis. A total of 300 µL of HPLC-grade hexane (Merck KGaA, Darmstadt, Germany) was used to elute the VOCs adsorbed onto the Tenax TA into a glass vial, from which 1 µL of the extract was injected into the chromatographic system. The extraction was performed at ambient temperature using a 50/30 µm DVB/CAR/PDMS StableFlex™ SPME fiber (Supelco, Bellefonte, PA, USA).

Dynamic headspace extraction continuously draws the surrounding air through the adsorbent, allowing the collection of volatile compounds emitted by fungal fruiting bodies under natural environmental conditions. This approach favors the recovery of highly volatile compounds present at low concentrations by continuously concentrating VOCs throughout the sampling period. Because the fruiting bodies remained attached to their natural substrate during sampling, the extracted VOCs were considered representative of the natural fungal volatilome under field conditions.

#### 2.2.2. Static Headspace Solid-Phase Microextraction (SHS-SPME) for Field VOC Collection

SHS sampling was conducted in the field by positioning the SPME fiber near the air intake of the DHS setup. The exposure time was 30 min. Sampling was performed inside a polyester sampling bag using a 50/30 µm DVB/CAR/PDMS StableFlex™ fiber (Supelco, Bellefonte, PA, USA) under ambient temperature conditions. Unlike DHS-SPME, SHS-SPME does not involve active airflow. Instead, volatile compounds accumulate within the enclosed headspace surrounding the fungal specimen and are passively adsorbed onto the SPME fiber. This methodology preserves the equilibrium composition of the volatilome under natural (in situ) conditions while minimizing disturbance of the fungal microenvironment. Similar to DHS-SPME, fruiting bodies remained attached to their original substrate throughout sampling, minimizing physiological alterations associated with specimen manipulation.

#### 2.2.3. Headspace Solid-Phase Microextraction (HS-SPME) for Laboratory VOC Extraction

Field-collected fungal samples were placed into 20 mL glass headspace vials (Supelco, Bellefonte, PA, USA) for laboratory analysis. VOC extraction was conducted under static headspace conditions at room temperature for 30 min using a 50/30 µm DVB/CAR/PDMS StableFlex™ fiber (Supelco, Bellefonte, PA, USA). Following exposure, the fiber was thermally desorbed in the injection port of the GC–MS system.

Laboratory HS-SPME analysis was performed under controlled experimental conditions to minimize environmental variability associated with temperature, air movement, and humidity. Consequently, this approach provides a complementary volatilomic profile that can be directly compared with the in situ extractions (DHS-SPME and SHS-SPME). Unlike the field-based extractions, laboratory analyses were performed after the fruiting bodies had been detached from their natural substrate and transported to the laboratory. This procedure interrupts water and nutrient exchange and exposes fungal tissues to handling and modified environmental conditions. Therefore, laboratory VOC profiles were interpreted as representing post-collection volatilomic changes potentially associated with physiological responses reported in the literature for fungal oxidative stress. Oxidative stress was not experimentally induced nor directly measured in this study; consequently, any stress-related interpretation is based on comparative metabolomic analysis and literature-supported biological inference.

The combined application of these three extraction strategies was intended to maximize VOC coverage, since each methodology exhibits different extraction efficiencies depending on compound volatility, polarity, and environmental conditions. Rather than representing experimentally induced biological treatments, these complementary extraction approaches provide distinct analytical windows for characterizing the fungal volatilome under field and post-collection conditions.

#### 2.2.4. Rationale for the Comparison Between In Situ and Laboratory VOC Profiles

The experimental design was based on comparing VOCs collected directly from fruiting bodies under natural field conditions with VOCs obtained after specimen collection and laboratory processing. The in situ extraction approaches (DHS-SPME and SHS-SPME) were performed while the fruiting bodies remained attached to their natural substrate, thereby representing the natural volatilomic profile under environmental conditions. In contrast, laboratory HS-SPME analysis was conducted after specimen removal and transport to the laboratory. Because detachment from the substrate interrupts water and nutrient exchange and exposes fungal tissues to handling and changes in environmental conditions, laboratory VOC profiles may reflect physiological responses associated with post-harvest stress, including metabolic alterations previously associated with oxidative stress in fungi. It should be emphasized that oxidative stress was not experimentally induced nor directly quantified through biochemical or molecular assays in this study. Therefore, any discussion regarding oxidative stress is based on comparative metabolomic observations and previous literature describing fungal physiological responses following tissue removal and environmental perturbation.

### 2.3. Gas Chromatography–Mass Spectrometry (GC–MS) Analysis

Analysis was performed using a Shimadzu GCMS-QP2010 Plus gas chromatograph coupled to a quadrupole mass spectrometer (Shimadzu Corporation, Kyoto, Japan). A ZB-5 fused-silica capillary column (Phenomenex Inc., Torrance, CA, USA) (30 m length × 0.25 mm internal diameter × 0.25 µm film thickness) was employed. The SPME fiber was thermally desorbed in the injector port at 250 °C in splitless mode prior to chromatographic separation. The injector temperature was set at 250 °C, while the ion source and interface were maintained at 300 °C [8]. The temperature program began at 40 °C, increased to 280 °C at a rate of 6 °C min^−1^, followed by a rapid increase to 300 °C at 30 °C min^−1^ and held for 1 min, resulting in a total run time of 41.67 min. Helium (99.999% purity) was used as the carrier gas at a constant flow rate of 1.0 mL min^−1^ in SPME injection mode.

Mass spectra were acquired under electron ionization (EI) at 70 eV. Data acquisition was performed using GCMSsolution software version 4.30 (Shimadzu Corporation, Kyoto, Japan) in full-scan mode over an m/z range of 40–500, enabling the qualitative characterization of fungal volatile organic compounds. Instrument performance and chromatographic stability were periodically verified through the analysis of quality control (QC) samples interspersed throughout the analytical sequence, as described in Section 2.6.

### 2.4. Molecular Identification of Fungal Species

The molecular identification of fungal species was carried out by CorpoGen Research and Biotechnology S.A.S. (Bogotá, Colombia; NIT 830.009.610-5). Mycelial material obtained from the selected fungal specimens was submitted for genomic DNA extraction and purification. The internal transcribed spacer (ITS) region of the ribosomal DNA was amplified by polymerase chain reaction (PCR) using the universal fungal primers ITS5 (5′-GGAAGTAAAAGTCGTAACAAGG-3′) and ITS4 (5′-TCCTCCGCTTATTGATATGC-3′). PCR products were subsequently purified and sequenced using the Sanger chain-termination method. Following sequencing, sequence editing and assembly were performed to generate consensus sequences for each specimen prior to taxonomic assignment.

Taxonomic identification was conducted using the BLASTn algorithm, version 2.17.0, available through the National Center for Biotechnology Information (NCBI), complemented with comparisons against the UNITE fungal database, GenBank, EMBL (European Molecular Biology Laboratory), DDBJ (DNA Data Bank of Japan), RefSeq, and the MycoID platform (MycoBank/BioloMICS). The final molecular identification was established according to the highest sequence identity obtained among the consulted databases. For several specimens, molecular identification could not be completed because the extracted DNA was insufficient or did not meet the quality requirements for successful PCR amplification and sequencing. Consequently, these specimens were maintained at the level of morphological identification and are reported in the results section.

### 2.5. VOC Identification

VOC identification was performed using GCMSsolution software version 4.30 (Shimadzu Corporation, Kyoto, Japan) coupled to the Shimadzu GCMS-QP2010 Plus system. Compound annotation was based on mass spectral matching against the NIST14, NIST14S, Adams Essential Oil Library, Essential Oils Library, and FFNSC (Flavor and Fragrance Natural and Synthetic Compounds) libraries using a minimum similarity index (SI) threshold of 80%. When available, metabolite annotations were further supported by comparison with PubChem, the Kyoto Encyclopedia of Genes and Genomes (KEGG), and the Human Metabolome Database (HMDB), allowing the assignment of unique chemical identifiers for subsequent structural classification and metabolic pathway annotation.

The software performed automated baseline correction, noise smoothing, peak deconvolution, and chromatographic peak integration based on total ion chromatogram (TIC) peak areas. Identified VOCs were annotated according to their retention time (RT), mass-to-charge ratio (m/z), similarity index (SI), peak area, adduct information, PubChem identifier, and metabolomics identification level, according to the analytical capabilities of the GC–MS platform. Since compound identities were assigned exclusively by spectral library matching without confirmation using authentic reference standards or retention index verification, all metabolite annotations were considered putative (Metabolomics Standards Initiative, MSI Level 2). Consequently, all subsequent chemical classifications, metabolic pathway assignments, and biological interpretations presented in this study should be regarded as annotation-based inferences rather than experimentally confirmed metabolite identifications.

### 2.6. Quality Assurance and Quality Control (QA/QC)

To ensure the reliability and reproducibility of the analytical workflow, quality assurance (QA) and quality control (QC) procedures were implemented throughout sample preparation, chromatographic analysis, and data processing. System quality control (SQC) injections were performed using solvent blanks and quality control samples to evaluate instrument stability, chromatographic reproducibility, and mass spectrometer performance during the analytical sequence. A total of six QC injections were distributed throughout the batch, with one QC sample analyzed after every five experimental samples to continuously monitor analytical performance. QC injections were used to verify signal stability, retention time reproducibility, and instrument response, as well as to detect background noise, potential carryover, and signal drift during the analytical sequence. Procedural blanks consisting of extraction materials and solvents processed under the same analytical conditions were also included to identify contaminants originating from laboratory consumables, reagents, or the analytical system. Features consistently detected in blank samples were removed from the final data matrix prior to multivariate statistical analysis. Instrument performance was considered acceptable when QC samples exhibited highly consistent chromatographic profiles and clustered closely during principal component analysis (PCA), confirming the stability and reproducibility of the analytical platform throughout the study.

### 2.7. Structural Analysis of Metabolites

Identified metabolites were annotated, whenever possible, using the Human Metabolome Database (HMDB; https://www.hmdb.ca/ accessed on 19 August 2026) and the Kyoto Encyclopedia of Genes and Genomes (KEGG; https://www.genome.jp/kegg/ accessed on 19 August 2026). Depending on the availability of information, individual metabolites could be assigned HMDB identifiers, KEGG identifiers, both identifiers, or remain without database annotation. The complementary use of these databases increased the chemical coverage of the detected volatile organic compounds by providing structural, physicochemical, and biochemical information that supported subsequent metabolite classification.

Chemical enrichment analysis was performed using the MetaboAnalyst 6.0 platform [23], allowing the classification of metabolites into major chemical families and the statistical evaluation of enriched compound classes across the fungal volatilome. The enrichment analysis was performed exclusively from the annotated chemical identities of the detected VOCs and therefore reflects the statistical overrepresentation of chemical classes rather than direct evidence of metabolic activity. HMDB and KEGG annotations were subsequently employed to associate the identified metabolites with previously reported biochemical pathways for visualization and functional interpretation. However, these pathway assignments were based exclusively on database annotations and published biochemical knowledge. Therefore, the metabolic networks and pathway enrichments presented in this study should be interpreted as annotation-based hypotheses that provide a biochemical framework for discussion, rather than as experimental demonstrations of pathway activity in the analyzed fungal species.

### 2.8. Multivariate Analysis

A multivariate statistical analysis was performed using the Pythonversion 3.14.6 programming language through custom-developed scripts for data importation, quality verification, missing value imputation, scaling, normalization, and statistical analysis. Prior to multivariate analysis, VOC peak areas were standardized using Z-score normalization to minimize differences in signal intensity among variables and improve comparability across samples. Multivariate normality was assessed using the Henze–Zirkler (HZ) test to determine whether the dataset satisfied the assumptions required for parametric multivariate analyses. The outcome of this assessment was subsequently used to guide the selection of the most appropriate statistical methods. Exploratory analysis was performed using Principal Component Analysis (PCA) to evaluate the overall structure of the dataset, visualize sample distribution, identify potential clustering patterns, and assess analytical reproducibility through the inclusion of quality control (QC) samples. Scree plots and cumulative variance analyses were subsequently generated to determine the contribution of each principal component and evaluate the dimensionality of the volatilomic dataset. Sample relationships were further investigated by Hierarchical Cluster Analysis (HCA).

To evaluate the discrimination of samples according to extraction methodology, several supervised machine-learning algorithms were compared, including Naïve Bayes, Support Vector Machine (SVM), CatBoost, Balanced Bagging, XGBoost, Gradient Boosting, Logistic Regression, Balanced Random Forest, K-Nearest Neighbors (KNN), Partial Least Squares Discriminant Analysis (PLS-DA), and Random Forest (RF). Model performance was assessed using accuracy, precision, F1-score, and cross-validation accuracy, and the best-performing algorithms were subsequently compared. Random Forest was selected for downstream interpretation because it provided robust classification performance while allowing estimation of variable importance for sample discrimination. Volcano plot analysis was additionally performed to identify VOCs exhibiting statistically significant differential abundance between extraction methods. Variable importance scores and multivariate classifications were interpreted exclusively as statistical indicators of sample discrimination and should not be regarded as direct evidence of biological function, metabolic regulation, or causal biochemical mechanisms. Instead, these analyses were used to identify candidate discriminatory VOCs for subsequent interpretation in the context of published literature and metabolite annotation databases.

### 2.9. Construction of the Biosynthetic Pathway

Metabolite identifiers from the Human Metabolome Database (HMDB), MetaCyc [24], and the Kyoto Encyclopedia of Genes and Genomes (KEGG) [25] were retrieved, whenever available, through the Chemical Entities of Biological Interest (ChEBI) database (https://www.ebi.ac.uk/chebi/ accessed on 19 August 2026), which was used to harmonize metabolite annotations among databases. These identifiers were subsequently queried using a custom Python script to establish metabolite–pathway associations based on curated biochemical information available in the referenced databases. The resulting annotations enabled the construction of integrated metabolite–pathway networks, allowing visualization of the relationships between the identified VOCs and previously reported fungal metabolic pathways, as well as the recognition of major biosynthetic classes and potential metabolic precursors. The pathway reconstruction was based exclusively on bioinformatic annotation and database integration and did not involve metabolomic flux analysis, transcriptomic profiling, enzymatic assays, or gene expression experiments. Consequently, the reconstructed networks represent putative biochemical associations inferred from metabolite annotation rather than experimentally validated biosynthetic pathways or active metabolic fluxes in the analyzed fungal specimens. Accordingly, these analyses were intended to provide a functional biochemical framework for interpreting the detected VOCs and for comparison with previously reported fungal metabolic pathways, thereby facilitating hypothesis generation for future experimental validation.

## 3. Results

### 3.1. Metabolite Identification in Fungi

Metabolite identification from the fruiting bodies of wild mushrooms was performed on 15 collected macromycete specimens representing 14 taxonomically independent fungal taxa. These metabolite profiles were obtained from morphologically and, whenever possible, molecularly identified macromycete specimens. Two specimens (VCQ12 and VCQ17) corresponded to the genus *Laetiporus* and were collected from different locations within the Botanical Garden. Although both specimens belong to the same fungal genus, they were treated as independent biological samples because they originated from different substrates and microhabitats, allowing the evaluation of possible spatial variability in their volatilomic profiles. Although 15 fungal specimens were included, the complete dataset comprised 28 biological volatilomic samples because several specimens were analyzed using more than one extraction technique (DHS-SPME in situ, SHS-SPME in situ, and HS-SPME under laboratory conditions). Additionally, six system quality control (QC) injections were incorporated throughout the analytical sequence, yielding a total of 34 chromatographic datasets used for multivariate statistical analyses.

Five specimens were analyzed using the three complementary extraction techniques: dynamic headspace solid-phase microextraction conducted in situ (DHS-SPME), static headspace solid-phase microextraction conducted in situ (SHS-SPME), and headspace solid-phase microextraction performed under laboratory conditions (HS-SPME) [26]. For four fungal specimens, VOC extraction was possible using two of the three extraction techniques, whereas the remaining specimens were analyzed using only one technique because of sample availability and field conditions. The results obtained for each specimen, including the number of VOCs identified with each extraction methodology, are summarized in Table 1. The highest numbers of VOCs were detected in Lentinus sp. (41 VOCs), *Crepidotus* sp. (34 VOCs), Agaricales sp. (33 VOCs), and *Annulohypoxylon stygium* (31 VOCs), whereas *Auricularia nigricans* exhibited the lowest number of detected compounds (3 VOCs). Overall, VOC abundance varied considerably among fungal taxa and according to the extraction methodology employed, suggesting that both biological diversity and analytical strategy contributed to the observed volatilomic variability.

**Table 1:** Identification of Fungal Species and Techniques Used for VOCs.

Species Number	Collection	Morphological Identification	Molecular Identification	Technique Used	Number of Identified VOCs	Supplementary Information
**1**	VCQ6	*Morchella esculenta*	*Morchella esculenta*	HS-SPME in laboratory	6	Table S1
**2**	VCQ7	*Scleroderma meridionale*	NI **	DHS-SPME in situ	29	Table S2
				SHS-SPME in situ	18	Table S3
				HS-SPME in laboratory	31	Table S4
**3**	VCQ9	*Pleurotus djamor*	*Pleurotus djamor*	DHS-SPME in situ	22	Table S5
				HS-SPME in laboratory	24	Table S6
				SHS-SPME in situ	11	Table S7
**4**	VCQ12	*Laetiporus sulphureus*	*Laetiporus sulphureus*	HS-SPME in laboratory	20	Table S8
				SHS-SPME in situ	5	Table S9
**5**	VCQ13	*Polyporus* spp.	*Polyporus tricholoma*	HS-SPME in laboratory	10	Table S10
**6**	VCQ14	*Xylaria striata*	*Xylaria striata*	DHS-SPME in situ	9	Table S11
				HS-SPME in laboratory	15	Table S12
**7**	VCQ15	*Lentinus* sp.	NI **	HS-SPME in laboratory	41	Table S13
				SHS-SPME in situ	16	Table S14
**8**	VCQ16	*Auricularia* spp.	*Auricularia nigricans*	HS-SPME in laboratory	3	Table S15
**9**	VCQ17	*Laetiporus* sp.	NI **	HS-SPME in laboratory	28	Table S16
**10**	VCQ18	*Agarical* *	NI **	HS-SPME in laboratory	33	Table S17
**11**	VCQ19	*Crepidotus* sp.	NI **	HS-SPME in laboratory	34	Table S18
**12**	VCQ23	*Cookeina* sp.	NI **	DHS-SPME in situ	18	Table S19
				HS-SPME in laboratory	28	Table S20
				SHS-SPME in situ	26	Table S21
**13**	VCQ27	*Trametes* sp.	NI **	HS-SPME in laboratory	10	Table S22
**14**	VCQ28	*Stereum* sp.	*Stereum sanguinolentum*	HS-SPME in laboratory	11	Table S23
				SHS-SPME in situ	28	Table S24
				DHS-SPME in situ	22	Table S25
**15**	VCQ30	*Panus similis*	*Panus similis*	DHS-SPME in situ	7	Table S26
				SHS-SPME in situ	5	Table S27
				HS-SPME in laboratory	20	Table S28

* Identification was resolved only to the taxonomic order Agaricales because neither morphological nor molecular evidence allowed a more specific assignment. ** NI: Molecular identification was not achieved because the purity of the mycelium in each sample was insufficient for sequencing.

### 3.2. Structural Analysis of the Fungal Volatilome

**Figure 1:**
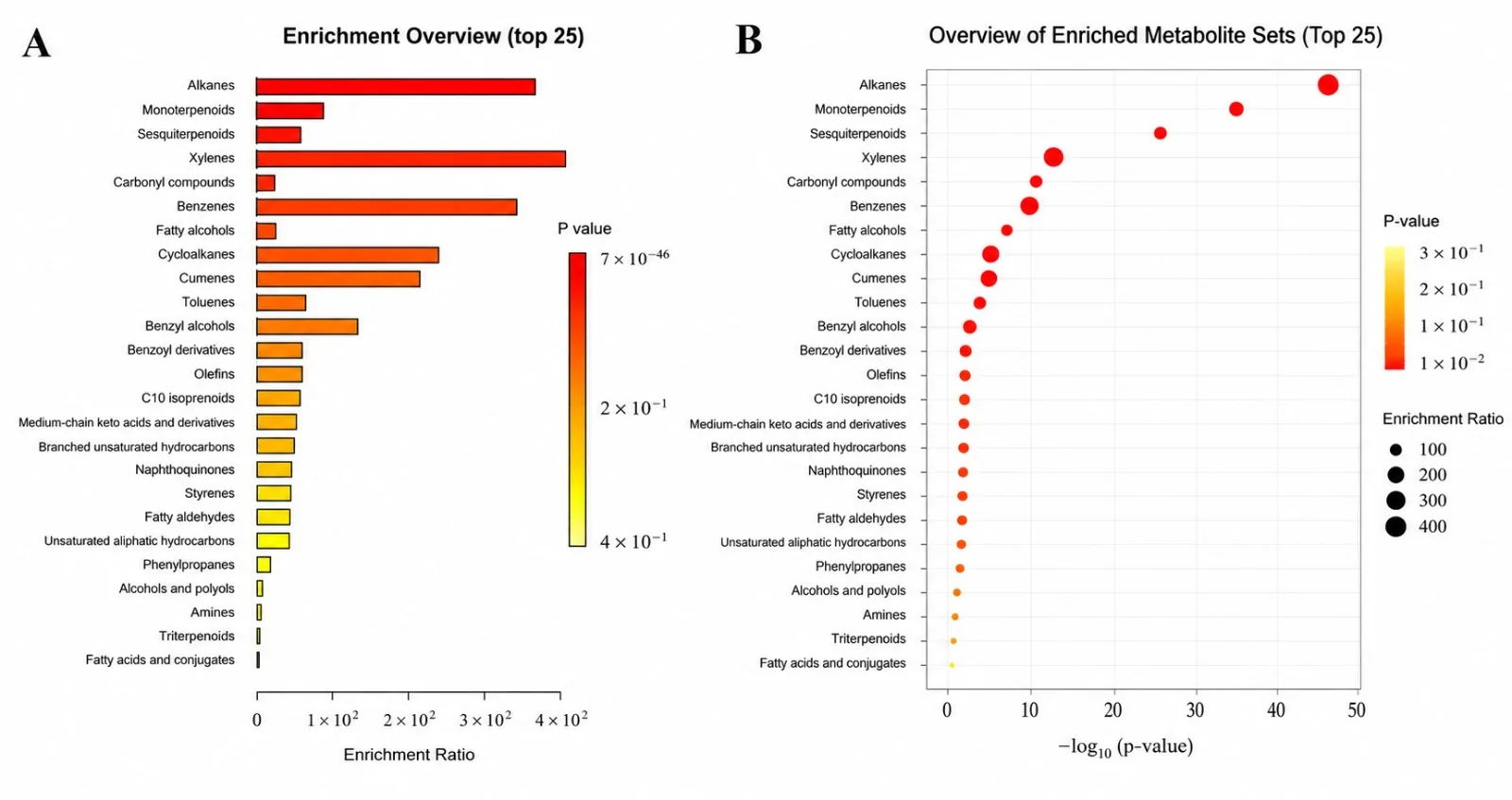
(**A**). Results of metabolite enrichment analysis by chemical structure. (**B**). Bubble plot for significance of metabolite categories identified.

Based on the volatile organic compounds (VOCs) identified in the different fungal samples, KEGG or HMDB identifiers were assigned to each metabolite whenever database information was available. The available KEGG and HMDB identifiers were subsequently used as input in MetaboAnalyst 6.0 to perform a chemical enrichment analysis based on metabolite structural classification. A total of 25 enriched metabolite classes were identified, providing an overview of the predominant chemical families represented within the fungal volatilome (Figure 1A).

Figure 1A presents a horizontal bar chart showing the enriched metabolite classes according to their Enrichment Ratio, while the color scale represents the statistical significance (*p*-value), ranging from yellow (lower significance) to dark red (higher significance). The enrichment analysis showed that Alkanes, Monoterpenoids, Sesquiterpenoids, Xylenes, Carbonyl compounds, and Benzenes exhibited the highest enrichment ratios (>500) together with the lowest *p*-values, indicating that these chemical classes constitute the predominant structural groups within the detected fungal volatilome. In contrast, chemical classes such as Amines, Fatty acids and conjugates, Triterpenoids, and other minor metabolite families displayed comparatively lower enrichment ratios and statistical significance.

Figure 1B complements the enrichment analysis by displaying the statistical significance of the enriched metabolite classes as a bubble plot. In this representation, the x-axis corresponds to the enriched metabolite sets, whereas the y-axis represents the –log_10_(*p*-value). Bubble size is proportional to the enrichment ratio, while the color gradient reflects statistical significance, ranging from yellow to dark red. Large red bubbles located in the upper-right region of the plot correspond to highly enriched and statistically significant metabolite classes, particularly Alkanes, Monoterpenoids, and Sesquiterpenoids. Conversely, smaller yellow bubbles represent metabolite classes with lower enrichment ratios and reduced statistical significance, including Amines, Triterpenoids, and Fatty acids and conjugates. Overall, the structural enrichment analysis indicates that the fungal volatilome is predominantly composed of hydrocarbon- and terpene-related compounds, whereas nitrogen-containing compounds and complex lipid-derived metabolites are comparatively less represented. These results provide a global overview of the chemical diversity detected among the analyzed fungal species and establish the basis for the subsequent multivariate and pathway analyses.

### 3.3. Multivariate Analysis

#### 3.3.1. Principal Component Analysis

Multivariate statistical analysis was performed using a volatilomic dataset generated from 15 collected macromycete specimens representing 14 taxonomically independent fungal taxa. Although 15 fungal specimens were included in the study, two specimens (VCQ12 and VCQ17) belonged to the same fungal genus (*Laetiporus*) but were collected from different sampling locations within the Botanical Garden. Consequently, they were considered independent biological specimens because they represent distinct environmental collections. The final analytical dataset consisted of 28 biological volatilomic samples, since several fungal specimens were analyzed using more than one extraction methodology. Specifically, volatile organic compounds (VOCs) were obtained using three complementary extraction approaches: Dynamic Headspace Solid-Phase Microextraction performed in situ (DHS-SPME), Static Headspace Solid-Phase Microextraction performed in situ (SHS-SPME), and laboratory-based Headspace Solid-Phase Microextraction (HS-SPME). Each extraction generated an independent volatilomic profile for the corresponding fungal specimen, thereby increasing the number of biological observations available for statistical analysis [27]. Additionally, six Quality Control (QC) injections were incorporated throughout the analytical sequence to monitor instrumental stability and analytical reproducibility, resulting in a final multivariate matrix composed of 34 chromatographic datasets (28 biological samples and 6 QC injections).

The chromatographic data obtained by HS-SPME-GC-MS were organized into a multivariate matrix consisting of 34 observations (rows) and 401 detected volatile variables (columns). Each row corresponded to an individual chromatographic acquisition (biological sample or QC injection), whereas each column represented a detected volatile organic compound (VOC) characterized by its chromatographic and mass spectral features. Prior to statistical analysis, the dataset underwent quality verification and preprocessing using custom Python scripts specifically developed for volatilomic data analysis. Peak intensities were standardized using Z-score normalization, minimizing differences in signal magnitude among variables while preserving the intrinsic biological variability of the samples. Subsequently, multivariate normality was evaluated using the Henze–Zirkler (HZ) test. The obtained *p*-value (<0.05) indicated that the dataset did not satisfy the assumptions of multivariate normality, thereby supporting the application of multivariate statistical methods that do not rely on Gaussian data distributions.

**Figure 2:**
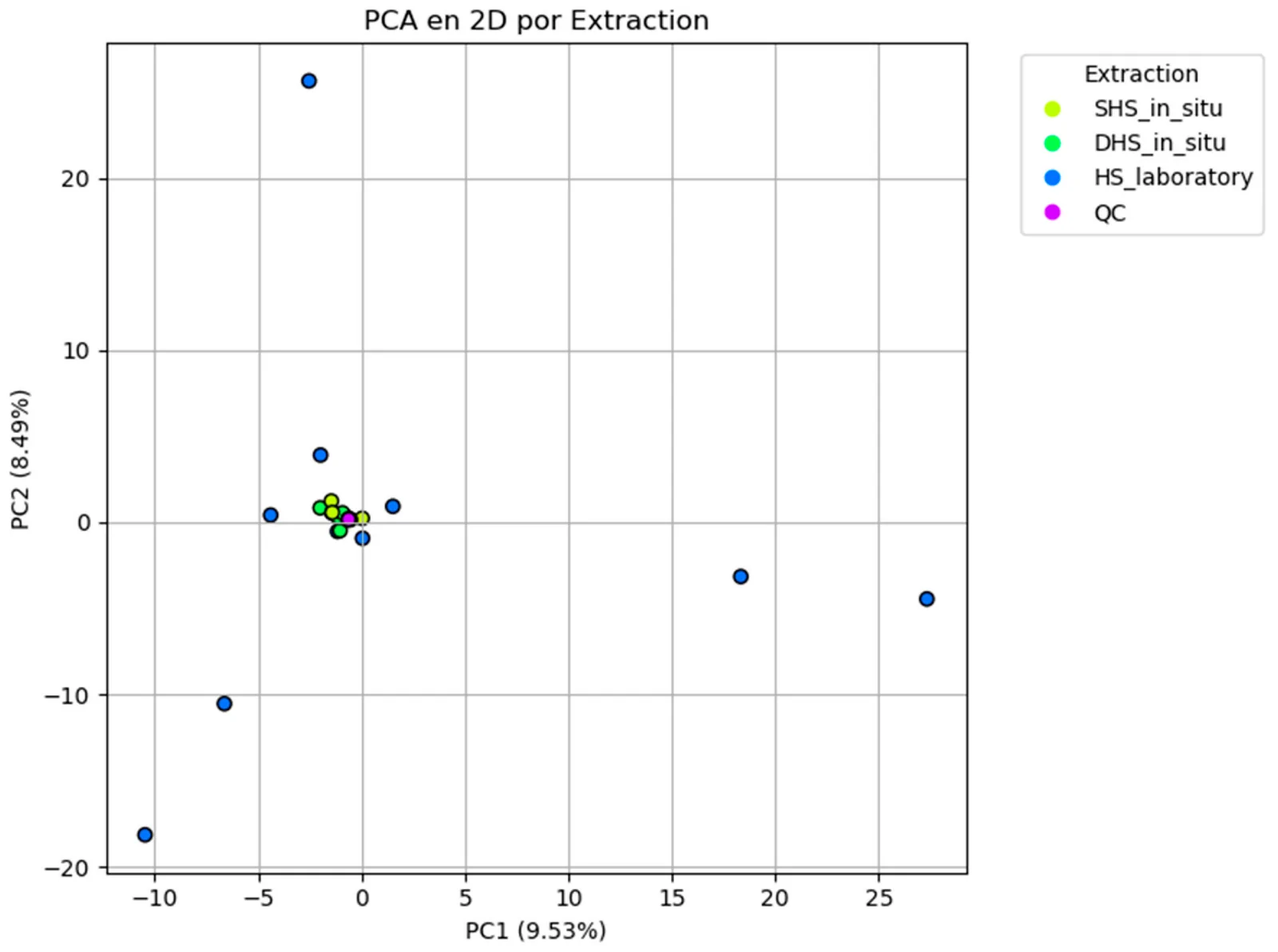
Data exploration through Principal Component Analysis (PCA).

Based on these preliminary evaluations, an exploratory analysis was performed using Principal Component Analysis (PCA) (Figure 2). PCA was selected as an unsupervised dimensionality reduction technique because it enables visualization of the overall structure of complex volatilomic datasets without imposing predefined sample classes. The first principal component (PC1) explained 9.53% of the total variance, whereas the second principal component (PC2) explained 8.49%, resulting in a cumulative explained variance of 18.02% for the first two dimensions.

Although the cumulative variance explained by PC1 and PC2 appears relatively modest, this behavior is expected for untargeted volatilomic datasets composed of hundreds of chemically diverse metabolites. In these datasets, the overall biological variability is typically distributed across numerous orthogonal principal components because individual VOCs contribute independently to the chemical diversity of the samples. Consequently, the total variance is not concentrated within the first two or three principal components, but rather progressively distributed among multiple latent dimensions. This characteristic reflects the intrinsic complexity of fungal volatilomes rather than a limitation of the PCA model itself. The cumulative variance analysis presented in the following section further demonstrates that additional principal components progressively contribute to the explained variance, confirming that the volatilomic information is inherently multidimensional.

Despite the relatively low variance explained by the first two principal components, the PCA score plot provided valuable information regarding the spatial organization of the fungal volatilomes and the overall relationships among samples. The analysis integrated volatilomic profiles obtained from different fungal taxa together with the three extraction methodologies employed throughout the study, allowing visualization of trends associated with both biological diversity and analytical methodology. Although complete class separation was not expected at this exploratory stage, PCA enabled the identification of clustering tendencies, the evaluation of sample dispersion, the detection of potential analytical outliers, and the preliminary assessment of similarities among extraction methodologies and fungal specimens. Therefore, PCA served primarily as an exploratory visualization tool prior to the application of supervised classification methods.

An additional objective of the PCA was the evaluation of analytical reproducibility through the incorporation of Quality Control (QC) samples. The QC injections formed a compact cluster within the multivariate space, demonstrating excellent instrumental stability and high reproducibility of the chromatographic analyses throughout the analytical sequence. The close grouping of QC samples indicates minimal analytical drift, negligible carryover effects, and low instrumental variability, confirming that the observed differences among fungal samples predominantly reflect biological and methodological variation rather than analytical artifacts. Because PCA is fundamentally an unsupervised exploratory technique, it was not intended to maximize sample discrimination. Accordingly, robust discrimination among fungal volatilomes was subsequently investigated using supervised machine-learning approaches. Thirteen classification algorithms, including Partial Least Squares Discriminant Analysis (PLS-DA), were evaluated and compared, with Random Forest exhibiting the best overall predictive performance. Therefore, the conclusions regarding sample discrimination presented in this study are primarily supported by the supervised analyses described in the following sections rather than by PCA alone.

#### 3.3.2. Main Contributors to Variability

**Figure 3:**
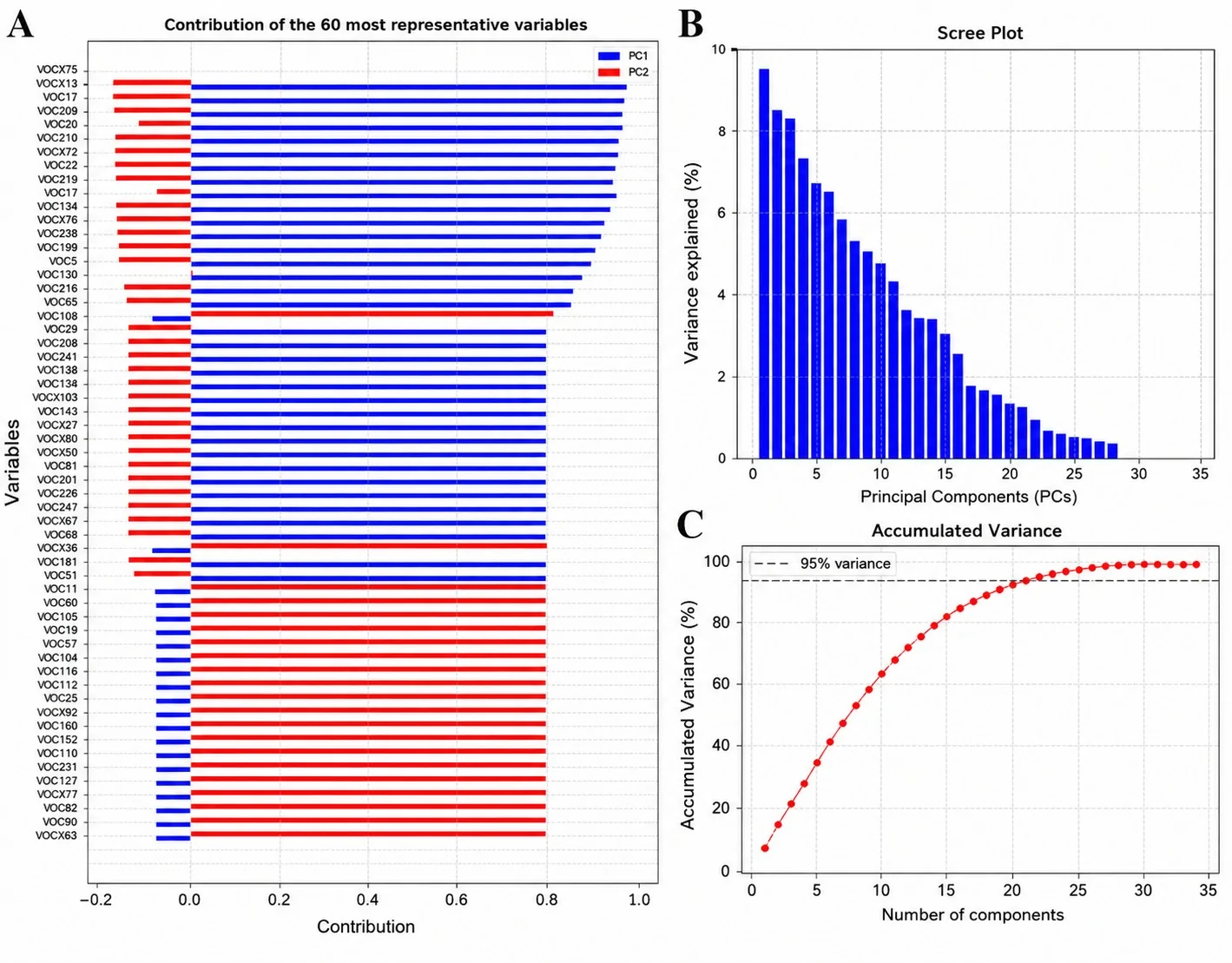
(**A**). Contribution plot of the most representative variables to Principal Components 1 and 2. (**B**). Total variance explained by each principal component. (**C**). Cumulative variance analysis across the sum of principal components.

Following the construction of the PCA model, the contribution of individual volatile variables to the principal components was evaluated to identify the metabolites responsible for the major sources of variation within the fungal volatilome. Figure 3A presents the contribution plot of the 60 variables exhibiting the highest loadings for the first two principal components. Variables contributing predominantly to PC1 are represented in blue, whereas those contributing mainly to PC2 are shown in red. PC1 accounted for the largest proportion of the explained variance (9.53%) and was primarily influenced by VOCX75, VOCX13, VOC117, VOC209, VOC20, VOC120, VOCX72, VOC22, VOC219, VOC173, VOC134, VOCX76, VOC238, VOC199, VOC75, VOC130, and VOC216. Conversely, the variables contributing most strongly to PC2 included VOC53, VOC11, VOC60, VOC105, VOC19, VOC57, VOC104, VOC116, VOC112, VOC25, VOCX92, VOC160, VOC152, VOC110, VOCX23, VOC122, VOCX77, VOCX78, VOC82, VOC90, VOCX63, VOC68, and VOC65. These variables represent the VOCs exerting the greatest influence on the spatial distribution of samples within the PCA score plot and therefore constitute the primary contributors to the chemical variability observed among the analyzed fungal volatilomes.

Figure 3B presents the Scree Plot, illustrating the percentage of variance explained by each principal component. As expected, PC1 explained the highest proportion of variance (9.53%), followed by progressively smaller contributions from subsequent principal components. Rather than showing an abrupt decrease after the first few components, the Scree Plot reveals a gradual decline in explained variance, indicating that the chemical information contained within the fungal volatilome is distributed across numerous latent dimensions. This behavior is characteristic of untargeted volatilomic datasets, where hundreds of metabolites contribute simultaneously and independently to overall biological variability. An important observation derived from the Scree Plot is that incorporation of an additional third principal component would increase the cumulative explained variance only modestly. Consequently, a three-dimensional PCA representation would not substantially improve the visualization of the dataset or alter the biological interpretation obtained from the exploratory analysis. Instead, the results demonstrate that no single principal component dominates the overall variability, reflecting the intrinsic chemical complexity of fungal VOC profiles.

The cumulative variance analysis (Figure 3C) provides additional insight into the dimensional structure of the dataset. The accumulated variance increased progressively with the incorporation of successive principal components, indicating that approximately ten components were required to explain nearly 70% of the total variance, whereas approximately fifteen principal components captured around 85% of the dataset variability. The cumulative variance exceeded 95% only after the inclusion of approximately twenty principal components. These findings demonstrate that the volatilomic dataset possesses a highly multidimensional structure, in which biologically relevant information is distributed among numerous independent variables rather than concentrated within only a few components. Collectively, the Scree Plot and cumulative variance analyses confirm that dimensionality reduction in fungal volatilomic data cannot be adequately represented using only the first two or three principal components. Instead, multiple orthogonal dimensions are required to preserve the underlying chemical information contained in the dataset. This behavior is consistent with the high molecular diversity characteristic of untargeted GC–MS volatilomic analyses and further justifies the subsequent application of supervised machine-learning algorithms to achieve robust sample discrimination and identify the VOCs contributing most strongly to class separation.

#### 3.3.3. Differentiation Among Samples

**Figure 4:**
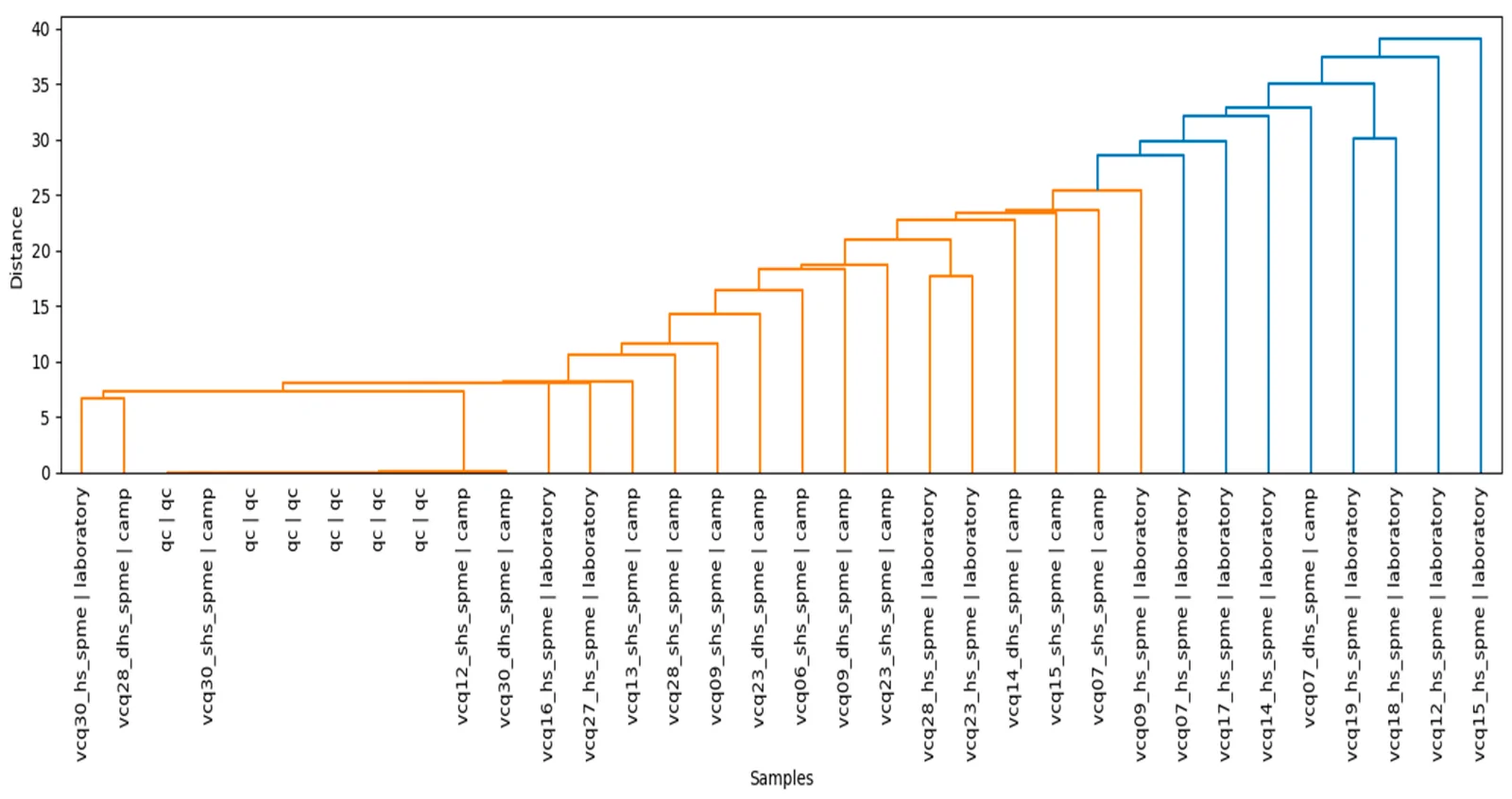
Hierarchical cluster analysis. The dendrogram was generated using Euclidean distance and Ward’s linkage method based on the 60 most representative VOC variables. Orange branches represent the main cluster comprising predominantly field (in situ) and quality control (QC) samples, whereas blue branches represent the cluster comprising predominantly laboratory-extracted samples.

To further investigate the relationships among fungal volatilomic profiles, an unsupervised Hierarchical Cluster Analysis (HCA) was performed using the complete VOC dataset (Figure 4). The resulting dendrogram organized the analyzed samples into two major clusters, represented in orange and blue, according to the overall similarity of their volatile profiles. The orange cluster included all Quality Control (QC) injections, which grouped closely together, indicating high analytical reproducibility throughout the chromatographic sequence. This cluster also contained samples VCQ12, VCQ30, VCQ16, VCQ27, VCQ13, VCQ28, VCQ09, VCQ23, VCQ06, VCQ14, VCQ15, and VCQ07, which were primarily associated with the in situ extraction methodologies (DHS-SPME and SHS-SPME).

In contrast, the blue cluster comprised samples VCQ07, VCQ17, VCQ14, VCQ19, VCQ18, VCQ12, and VCQ15, which were mainly associated with the laboratory HS-SPME extraction approach. Several fungal specimens appeared more than once in the dendrogram because they were analyzed using different extraction methodologies, with each extraction generating an independent volatilomic profile included in the clustering analysis.

Overall, the hierarchical clustering analysis revealed reproducible grouping patterns based on the overall similarity of VOC profiles. The close clustering of the QC samples confirmed the analytical consistency of the chromatographic analyses, whereas the distribution of the biological samples demonstrated the presence of distinct volatilomic profiles across the analyzed fungal specimens. The HCA therefore provides a complementary exploratory representation of the relationships among samples prior to the application of supervised classification models.

#### 3.3.4. Model Identification

**Figure 5:**
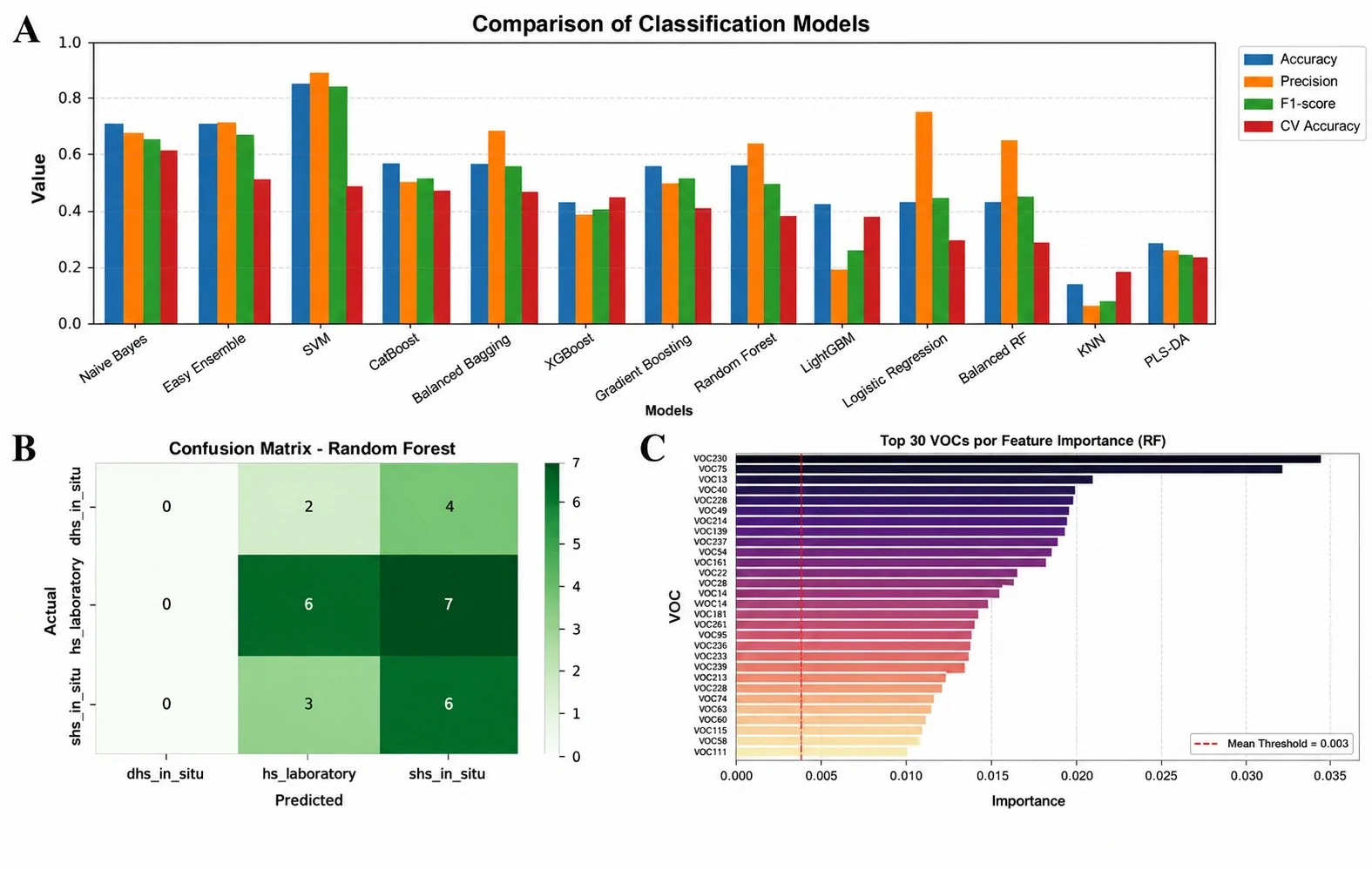
(**A**). Comparison between classification models. (**B**). Confusion matrix of the Random Forest model. (**C**). Variable importance (VIP) according to Random Forest.

Following the exploratory multivariate analyses, a supervised classification approach was implemented to evaluate the ability of different machine-learning algorithms to discriminate fungal volatilomic profiles according to the extraction methodology employed. Figure 5A summarizes the comparative performance of the evaluated classification models using accuracy, precision, F1-score, and cross-validation accuracy as performance metrics. A total of 13 supervised classification algorithms were evaluated, including Naïve Bayes, Easy Ensemble, Support Vector Machine (SVM), CatBoost, Balanced Bagging, XGBoost, Gradient Boosting, Random Forest, LightGBM, Logistic Regression, Balanced Random Forest (BRF), K-Nearest Neighbors (KNN), and Partial Least Squares Discriminant Analysis (PLS-DA). Among the evaluated models, SVM exhibited the highest overall classification metrics, whereas Random Forest also demonstrated high predictive performance and was selected for subsequent variable importance analysis.

The classification performance of the Random Forest model is presented in Figure 5B as a confusion matrix. The matrix summarizes the agreement between the observed and predicted sample classes according to the extraction methodology. The model correctly classified the majority of SHS-SPME in situ and HS-SPME laboratory samples, whereas a limited number of misclassifications were observed between the DHS-SPME in situ and SHS-SPME in situ groups.

The contribution of individual volatile compounds to the Random Forest classification model is presented in Figure 5C. The variable importance plot displays the 30 VOCs exhibiting the highest contribution to sample discrimination according to the mean decrease in impurity. Among the most relevant variables identified were VOC250 (Heneicosane), VOC75 (D-Limonene), VOC31 (*o*-Xylene), VOC209 (Caryophyllene), VOC56 (3-Octanone), VOC54 (1-Octen-3-ol), VOC61 (3-Octanol), VOC32 (*m*-Xylene), VOC234 (2,6-Di-*tert*-butyl-4-methylphenol), VOC207 (Longifolene), VOC236 (Dauca-4(11),8-diene), and VOC220 (2,6-Di-*tert*-butyl-*p*-benzoquinone), together with several additional metabolites that collectively contributed to the classification model.

**Figure 6:**
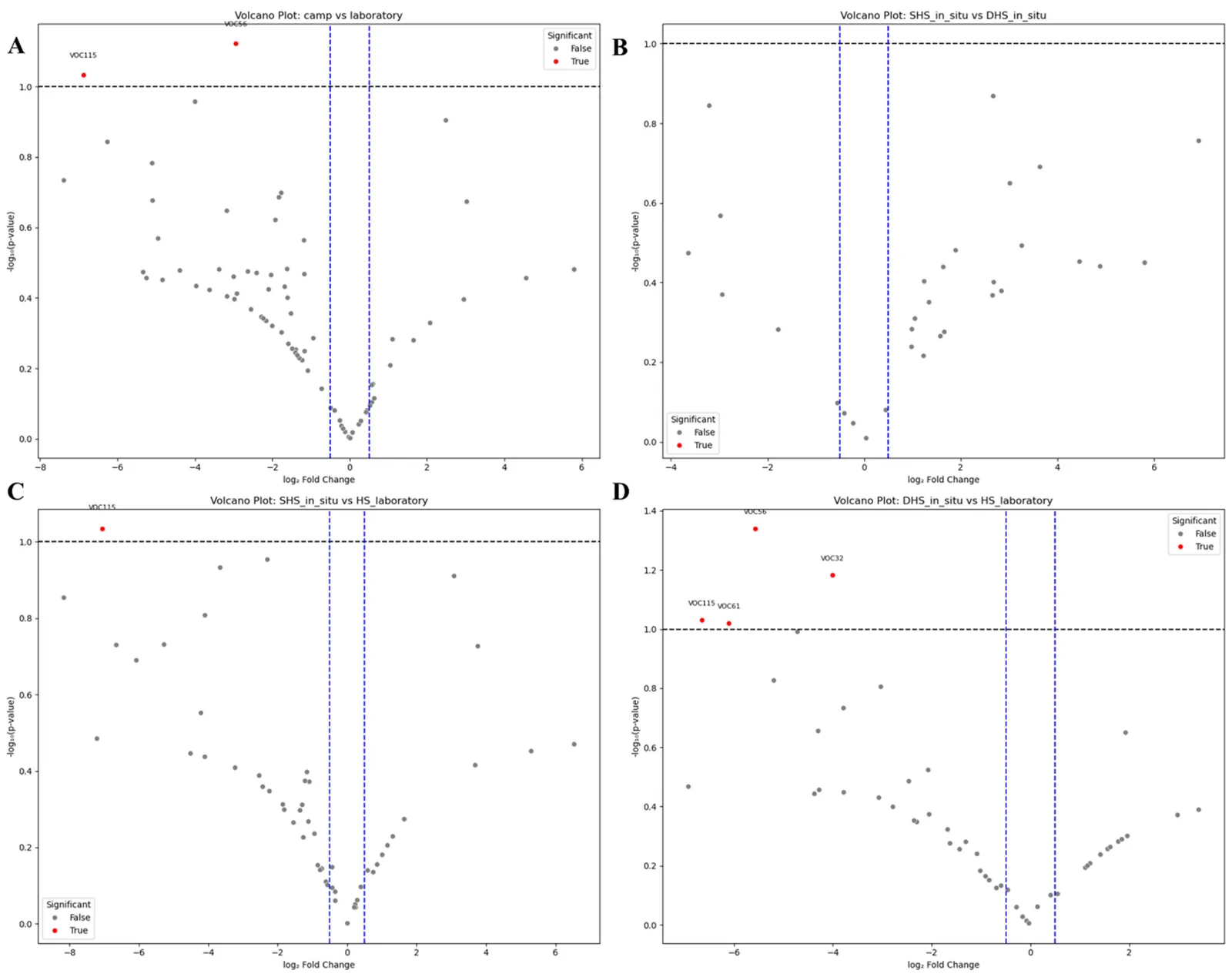
(**A**). Volcano plot comparing field vs. laboratory samples. (**B**). Volcano plot comparing SHS-SPME in situ vs. DHS-SPME in situ samples. (**C**). Volcano plot comparing SHS-SPME in situ vs. HS-SPME laboratory samples. (**D**). Volcano plot comparing DHS-SPME in situ vs. HS-SPME laboratory samples. The vertical blue dashed lines indicate the predefined log-fold-change thresholds used to assess the magnitude of differential VOC abundance, whereas the horizontal dashed line represents the statistical significance threshold.

To further examine differential metabolite abundance among extraction methodologies, volcano plot analyses were performed. Figure 6A compares fungal samples analyzed in situ with those processed under laboratory conditions, whereas Figure 6B–D present pairwise comparisons among the three extraction methodologies. Differentially abundant metabolites identified across these comparisons included VOC56 (3-Octanone), VOC115 (Linalool), VOC32 (*m*-Xylene), and VOC61 (3-Octanol). The number and distribution of significant VOCs varied according to the specific comparison, indicating differences in metabolite abundance among the extraction approaches evaluated.

### 3.4. Metabolic Pathway Analysis

**Figure 7:**
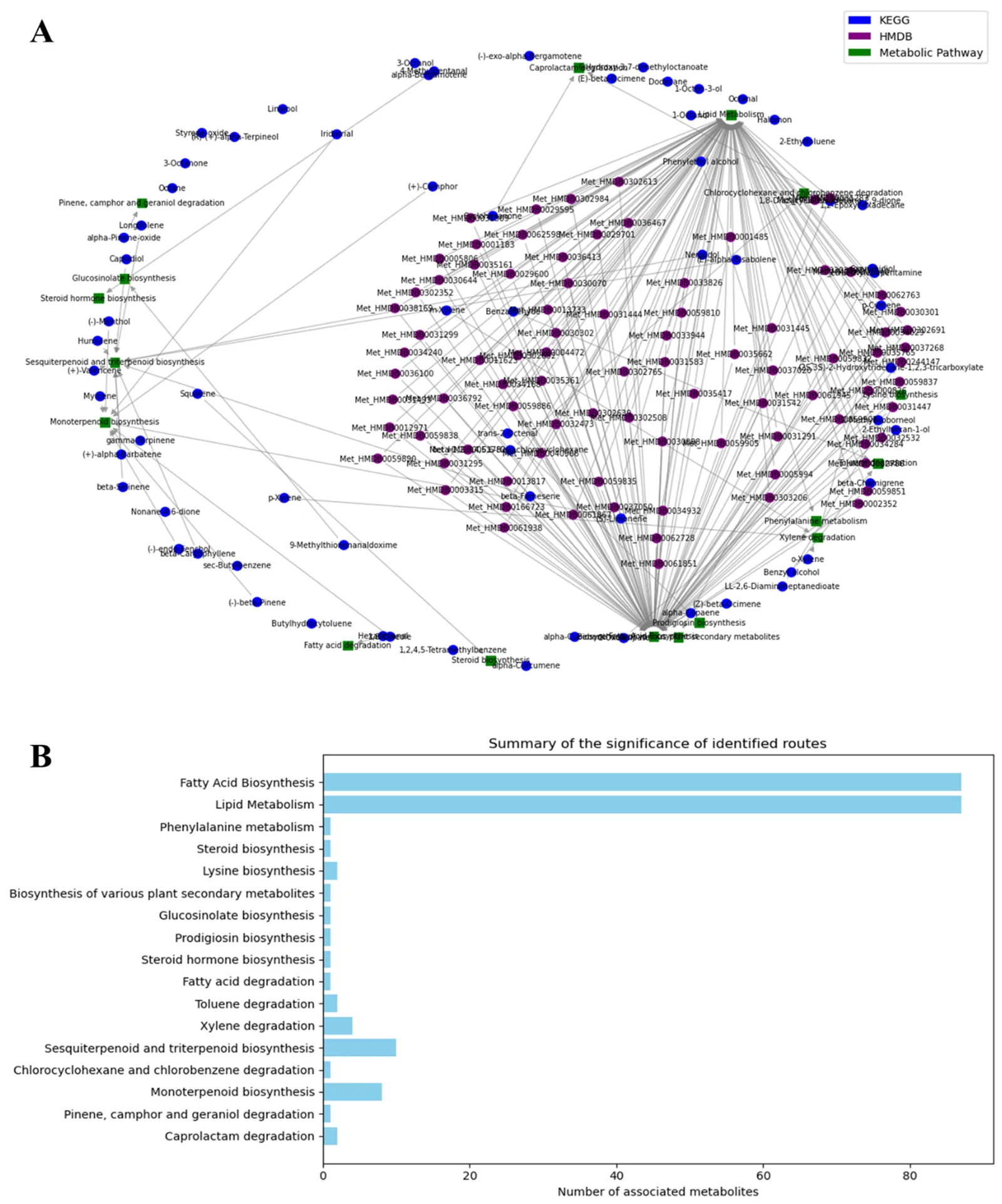
(**A**). Integrated metabolic network. (**B**). Summary relevance of metabolic pathways.

To investigate the biochemical relationships among the identified volatile organic compounds (VOCs), metabolite annotation was performed using KEGG and HMDB identifiers whenever database information was available. The annotated metabolites were subsequently processed using a Python-based workflow to retrieve their corresponding biochemical pathways and to construct a metabolite–pathway association network. Figure 7A presents the resulting network generated from the integrated KEGG and HMDB annotations. In this representation, blue nodes correspond to metabolites annotated in the Kyoto Encyclopedia of Genes and Genomes (KEGG), purple nodes represent metabolites identified in the Human Metabolome Database (HMDB), and green nodes denote the biochemical pathways associated with the annotated metabolites. The resulting network illustrates the connectivity between the detected VOCs and multiple annotated metabolic pathways, showing that several metabolites were associated with more than one biochemical route.

Figure 7B summarizes the pathway enrichment analysis obtained from the annotated metabolite dataset. The enrichment results indicate that Fatty Acid Biosynthesis and Lipid Metabolism were the pathways associated with the largest number of annotated VOCs, each comprising more than 80 metabolites. Additional pathways identified through the annotation process included Sesquiterpenoid and Triterpenoid Biosynthesis, Monoterpenoid Biosynthesis, Xylene Degradation, Toluene Degradation, and Lysine Biosynthesis, together with several other metabolic routes represented by smaller numbers of annotated compounds. The relative representation of these pathways reflects the distribution of the identified metabolites across the biochemical annotations retrieved from the KEGG and HMDB databases.

Overall, the metabolite annotation workflow enabled the association of the detected fungal VOCs with multiple biochemical pathways available in public metabolomic databases. The metabolite–pathway network together with the enrichment analysis provides a comprehensive overview of the biochemical annotations assigned to the identified volatile compounds and serves as the basis for the functional interpretation presented in the Discussion section.

### 3.5. Biochemical Pathways in Fungi

Fungi exhibit a highly diverse biochemistry involving both primary and secondary metabolism, with a remarkable capacity for the degradation of cellulose, lignin, aromatic compounds, and numerous xenobiotics, while actively participating in global carbon and nitrogen cycling. This metabolic versatility enables the production of a wide variety of volatile organic compounds (VOCs) involved in ecological communication, chemical defense, substrate colonization, and interactions with plants, microorganisms, and insects. The pathway enrichment analysis performed in the present study identified Fatty Acid Biosynthesis, Lipid Metabolism, Sesquiterpenoid and Triterpenoid Biosynthesis, Monoterpenoid Biosynthesis, and several aromatic compound degradation pathways as the principal biochemical routes associated with the annotated VOCs [28,29]. Because these pathway assignments were obtained through metabolite annotation using KEGG and HMDB databases, they should be interpreted as bioinformatic associations supported by previously published biochemical evidence rather than as direct experimental demonstrations of pathway activity in the analyzed fungal species.

Among the metabolites identified as the principal discriminant variables in the Random Forest model, several belong to the terpenoid family, including VOC75 (D-Limonene), VOC209 (Caryophyllene), VOC207 (Longifolene), and VOC236 (Dauca-4(11),8-diene). These compounds have previously been reported to originate from the cyclization of isoprenoid precursors synthesized through the mevalonate pathway in fungi, whereas the alternative MEP/DOXP pathway has mainly been described in bacteria and plants associated with fungal communities [30]. Fungal terpene synthases catalyze the conversion of geranyl pyrophosphate, farnesyl pyrophosphate, and geranylgeranyl pyrophosphate into mono-, sesqui-, and diterpenoids, generating compounds that participate in chemical communication, defense against competitors, and ecological interactions with insects and plants [30]. The detection of these terpenoids in several fungal specimens is therefore consistent with previous reports describing terpenoid biosynthesis as one of the major sources of fungal volatile metabolites.

The pathway enrichment analysis also revealed that Fatty Acid Biosynthesis and Lipid Metabolism represented the biochemical categories containing the highest number of annotated VOCs, with more than 80 associated metabolites. This observation agrees with previous studies showing that lipid metabolism constitutes one of the principal biochemical origins of fungal volatile compounds. Representative metabolites identified in the present study, including VOC250 (Heneicosane) [31], VOC253 (2-Methyloctacosane), VOC261 (Tetratetracontane), VOC199 (4-Ethyl-2-methylhexane), VOC95 (2,3,3-Trimethyloctane), VOC28 (2,3,4-Trimethylhexane), VOC56 (3-Octanone), VOC54 (1-Octen-3-ol), and VOC61 (3-Octanol), have previously been associated with fatty acid metabolism through the elongation of acetyl-CoA and malonyl-CoA by the Fatty Acid Synthase (FAS) complex, followed by oxidative reactions that generate aldehydes, alcohols, and ketones [31,32]. In fungi, monooxygenases and related oxidoreductases convert unsaturated fatty acids into characteristic eight-carbon volatiles such as 1-octen-3-ol, widely recognized as the characteristic “mushroom alcohol” [32].

Previous biochemical studies have shown that linoleic acid may be oxidized to 10-hydroperoxide (10-HPOD) through the action of dioxygenases [33]. Subsequently, hydroperoxide lyases catalyze the cleavage of 10-HPOD, producing 1-octen-3-ol together with 10-oxodecanoic acid, whereas additional oxidation-reduction reactions may generate 3-octanone and 3-octanol [34,35]. The occurrence of these metabolites in the present volatilomic dataset is therefore consistent with lipid oxidation pathways previously described in fungi, although no enzymatic or transcriptomic analyses were performed in this study to confirm the activation of these biochemical routes.

Besides lipid-derived metabolites, several aromatic and polycyclic compounds were also identified among the most relevant discriminatory VOCs, including VOC31 (*o*-Xylene), VOC32 (*m*-Xylene), VOC4 (Methyl *N*-hydroxybenzenecarboximidate), VOC234 (2,6-Di-*tert*-butyl-4-methylphenol), VOC220 (2,6-Di-*tert*-butyl-*p*-benzoquinone), and VOC41 (Cyclohexanone). These metabolites have previously been associated with the transformation of aromatic amino acids such as phenylalanine and tyrosine or with secondary metabolism mediated by fungal polyketide synthases (PKSs) [35]. PKS enzymes catalyze the sequential condensation of malonyl-CoA units, generating structurally diverse aromatic metabolites that frequently participate in ecological interactions and stress responses.

The pathway enrichment analysis additionally associated several detected metabolites with xylene degradation, toluene degradation, and related aromatic transformation pathways. Although these annotations are consistent with the remarkable metabolic versatility of fungi and with previous reports describing fungal degradation of aromatic compounds and environmental pollutants [36,37], the present study cannot determine whether these VOCs originated exclusively from endogenous fungal metabolism or whether some compounds may have been influenced by the surrounding environmental matrix. Since the analyzed fruiting bodies were collected directly from natural substrates within the Botanical Garden, adsorption of environmental volatile compounds cannot be completely excluded. Therefore, these pathway assignments should be interpreted as plausible biochemical associations supported by metabolite annotation and literature rather than definitive evidence of active degradation processes in the analyzed specimens.

Overall, the integration of metabolite annotation, pathway enrichment analysis, and multivariate statistics provides a comprehensive biochemical framework for interpreting the fungal volatilome. Rather than demonstrating the activation of specific biosynthetic pathways, the present results identify candidate metabolic processes that are consistent with previous reports on fungal metabolism and provide a basis for future studies involving transcriptomics, proteomics, enzyme activity measurements, or stable-isotope labeling to experimentally validate the biosynthetic origin of the identified VOCs.

## 4. Discussion

### 4.1. Metabolite Identification in Fungi

The use of multiple extraction techniques (DHS-SPME, SHS-SPME, and HS-SPME) highlights the methodological complexity required to achieve a comprehensive characterization of fungal volatile organic compounds (VOCs). Each extraction approach is based on different physicochemical principles and therefore exhibits distinct affinities for volatile metabolites according to their molecular weight, polarity, vapor pressure, and abundance. Dynamic headspace extraction (DHS-SPME) continuously concentrates highly volatile compounds under natural environmental conditions, whereas SHS-SPME performed in situ preserves the equilibrium composition of the surrounding headspace. In contrast, laboratory HS-SPME provides controlled analytical conditions that minimize environmental variability during extraction. Consequently, the differences observed in the number and composition of VOCs recovered by each methodology are expected and indicate that these techniques provide complementary rather than redundant analytical information for fungal volatilomic studies.

The variability in the number of detected VOCs among fungal species also indicates substantial chemical diversity within the analyzed fungal specimens. Species such as *Lentinus* sp., *Crepidotus* sp., *Agaricales* sp., and *Annulohypoxylon stygium* yielded considerably richer volatilomic profiles than *Auricularia nigricans*, suggesting substantial interspecific differences in the detected VOC profiles. Such variability has been widely reported in fungal volatilomics and is commonly associated with differences in phylogeny, developmental stage, substrate composition, nutritional status, and environmental conditions that may influence secondary metabolite production. Therefore, the detected VOC diversity likely reflects the combined influence of taxonomic identity, physiological condition, substrate characteristics, and local environmental factors, rather than a single biological factor.

It is important to note that, according to the molecular identification results, samples VCQ12 and VCQ17 belong to the same fungal genus (*Laetiporus*). However, these specimens were collected from different locations within the Botanical Garden of the University of Caldas and were therefore exposed to potentially different local environmental conditions. Although they share a common taxonomic affiliation, differences in their volatilomic profiles may be associated with variations in microhabitat characteristics, including substrate composition, moisture availability, light exposure, microbial interactions, and other local environmental factors that have been reported to influence fungal secondary metabolism. These observations suggest that fungal VOC profiles may vary among specimens within the same taxonomic group and highlight the potential contribution of local environmental conditions to this variability.

Environmental conditions are well recognized as important factors associated with variation in fungal volatilomes. Factors such as humidity, temperature, nutrient availability, oxygen concentration, and interactions with surrounding microorganisms have been reported to influence VOC production and emission. Likewise, physiological responses associated with sample handling and transfer from natural conditions to laboratory environments may alter the abundance of certain volatile metabolites. Consequently, the complementary use of in situ and laboratory-based extraction strategies provides a broader representation of the fungal volatilome than would be obtained using a single analytical approach, allowing both environmentally emitted VOCs and metabolites detected under standardized analytical conditions to be incorporated into the overall chemical characterization. However, differences between field and laboratory profiles cannot be attributed specifically to oxidative stress in the present study, because no dedicated physiological, biochemical, or molecular measurements of oxidative stress were performed.

Overall, the combined analytical strategy adopted in the present study substantially increased metabolite coverage and indicated that the detected fungal volatilomic composition varied according to both biological characteristics of the specimens and extraction methodology. Rather than representing methodological bias, the differences observed among extraction techniques provide complementary information that improves the chemical characterization of fungal VOCs and may facilitate the identification of candidate discriminatory compounds for future biomarker studies and the investigation of VOCs of potential ecological or biotechnological interest.

### 4.2. Structural Analysis of the Fungal Volatilome

The structural enrichment analysis revealed that the fungal volatilome was characterized predominantly by hydrocarbons, terpenoids, carbonyl compounds, and aromatic metabolites. The predominance of these chemical classes is consistent with previous volatilomic studies reporting that fungal VOCs are largely composed of relatively apolar compounds with high volatility, particularly lipid-derived metabolites and terpenoids, which can be readily released into the surrounding environment. Although the enrichment analysis performed in this study was based on chemical annotation rather than direct metabolic measurements, the observed distribution of metabolite classes is consistent with the general chemical composition previously described for fungal volatilomes.

The enrichment of alkanes and carbonyl compounds may reflect the substantial contribution of lipid metabolism to fungal volatile production. Previous studies have shown that fatty acid degradation and oxidation pathways can generate a wide variety of volatile aldehydes, ketones, alcohols, and hydrocarbons during fungal growth and development. Consequently, the predominance of these compound classes observed in the present study is consistent with the important contribution reported for lipid-derived metabolites in fungal volatilomes. However, because no enzymatic activity or gene expression analyses were performed, these biochemical associations should be interpreted as literature-supported hypotheses derived from metabolite annotation rather than direct evidence of pathway activation.

Likewise, the strong enrichment of monoterpenoids and sesquiterpenoids is consistent with the widespread occurrence of terpene metabolism in fungi. Terpenoid biosynthesis has been extensively described as an important source of fungal secondary metabolites, and individual fungal terpenoids have been associated in the literature with ecological processes such as chemical communication, antimicrobial activity, competition with neighboring microorganisms, and interactions with plants and insects [30]. The recurrent detection of these metabolite classes across multiple fungal specimens therefore suggests that terpenoid-derived compounds contribute substantially to the chemical diversity observed in the analyzed macromycetes, although the activity of the corresponding biosynthetic pathways and their specific ecological functions were not experimentally evaluated in the present study.

Aromatic metabolites, including xylenes, benzene derivatives, and related compounds, were also represented among the enriched chemical classes. These metabolites have previously been associated with the metabolism of aromatic amino acids, polyketide biosynthesis, and the transformation of complex aromatic substrates. Nevertheless, caution should be exercised when interpreting these annotations because the analyzed fruiting bodies were collected directly from their natural environment. Consequently, some aromatic compounds may reflect interactions between fungal metabolism and the surrounding substrate or environmental matrix rather than being exclusively synthesized by the fungi themselves. Additional experimental approaches, such as stable-isotope labeling, transcriptomic analyses, or controlled cultivation experiments, would be required to distinguish endogenous fungal biosynthesis from environmental contributions.

The bubble plot further demonstrated that several chemical classes accounted for most of the statistical enrichment observed in the volatilomic dataset. The concentration of large, highly significant metabolite groups in the upper-right region of the plot indicates that these compound classes showed strong enrichment within the annotated dataset. Such enrichment patterns identify these chemical families as prominent components of the observed volatilome and suggest their potential value for future chemotaxonomic and comparative studies, although their consistency across fungal taxa and their biological significance would require validation using larger and independently replicated datasets.

Overall, the structural enrichment analysis provides a comprehensive overview of the chemical composition of the fungal volatilome and demonstrates that fungal VOCs encompass multiple metabolite classes commonly associated in the literature with lipid metabolism, terpene biosynthesis, and aromatic compound transformation. Rather than demonstrating the activation of specific metabolic pathways, these findings establish a chemical framework for interpreting fungal VOC diversity and identify metabolite classes that may serve as targets for future biochemical, ecological, chemotaxonomic, and functional investigations.

### 4.3. Multivariate Analysis

The multivariate analyses demonstrate the remarkable complexity of the fungal volatilome, as reflected by the relatively low proportion of variance explained by the first two principal components. Although PC1 and PC2 together accounted for only 18.02% of the total variance, this behavior is commonly observed in untargeted metabolomic and volatilomic datasets, where hundreds of chemically diverse metabolites contribute simultaneously to biological variability. Rather than indicating poor model performance, the dispersion of variance across multiple components reflects the intrinsic chemical heterogeneity of fungal VOC profiles. Consequently, PCA fulfilled its primary objective as an exploratory method by revealing the overall structure of the dataset, identifying clustering tendencies, and assessing analytical reproducibility through the quality control samples.

The scree plot and cumulative variance analyses further support this interpretation. Approximately twenty principal components were required to explain nearly 95% of the total variance, demonstrating that the volatilomic information is distributed across numerous independent dimensions rather than concentrated within only a few variables. This behavior is consistent with the highly diverse chemical composition of fungal volatilomes, in which multiple metabolite classes—including lipid-derived compounds, terpenoids, aromatic compounds, alcohols, ketones, and hydrocarbons—collectively contribute to sample variability. Therefore, the relatively low variance explained by the first two principal components should not be interpreted as a limitation of the PCA itself but rather as a reflection of the inherent complexity of the analyzed metabolomic dataset. In this context, the PCA served as an exploratory visualization tool, whereas subsequent supervised analyses were employed to improve sample discrimination and identify the metabolites contributing most strongly to class separation.

The variable contribution analysis revealed that only a limited subset of VOCs exerted a major influence on the first principal components. Several of these metabolites correspond to compounds previously reported in fungal volatilomes, including terpenoids such as D-limonene and caryophyllene, lipid-derived compounds including 1-octen-3-ol, 3-octanone, and 3-octanol, together with several aromatic hydrocarbons. Their high contribution to the multivariate models suggests that these compounds represent major sources of variability within the analyzed volatilomic dataset. Nevertheless, their statistical importance should be interpreted as an indicator of discriminatory capacity rather than direct evidence of biological function.

Hierarchical Cluster Analysis (HCA) demonstrated that the extraction methodology strongly influenced the organization of samples within the multivariate space. Samples obtained using the two in situ extraction techniques (DHS-SPME and SHS-SPME) exhibited greater similarity to one another than to samples analyzed by laboratory HS-SPME, indicating that extraction conditions contributed substantially to the observed volatilomic variation. This clustering pattern suggests that environmental sampling conditions and analytical methodology influence the composition of the detected VOC profiles. Because the laboratory analyses were performed under controlled conditions following specimen transport, differences between field and laboratory profiles may reflect changes associated with sample handling, environmental exposure, or physiological responses occurring after collection. However, the present study did not directly evaluate oxidative stress biomarkers, gene expression, or enzymatic activity, and therefore the observed volatilomic differences cannot be unequivocally attributed to oxidative stress alone.

The supervised classification analyses further demonstrated that fungal volatilomic profiles contain sufficient chemical information to discriminate samples according to extraction methodology. Although the Support Vector Machine (SVM) model achieved slightly higher predictive performance, the Random Forest classifier was selected for biological interpretation because it provides quantitative estimates of variable importance while maintaining excellent predictive accuracy and robustness. The Variable Importance analysis identified several VOCs that consistently contributed to sample discrimination, including 3-octanone, 1-octen-3-ol [32], D-limonene, caryophyllene, m-xylene, longifolene, and 3-octanol. These compounds therefore represent candidate discriminatory metabolites within the analyzed fungal volatilome.

The volcano plot analyses provided an independent statistical approach for evaluating differential metabolite abundance among extraction methodologies. Several metabolites identified as important by the Random Forest model, including 3-octanone, linalool, m-xylene, and 3-octanol, also exhibited significant fold changes in pairwise comparisons between extraction conditions. The convergence of these independent analytical approaches increases confidence in the reproducibility of the statistical findings and suggests that these VOCs consistently contribute to the observed differences among volatilomic profiles. Nevertheless, additional biochemical and physiological studies will be required to determine the biological mechanisms responsible for these differences.

Overall, the integration of unsupervised multivariate analysis, supervised machine-learning classification, variable importance analysis, and differential abundance testing provides a comprehensive statistical framework for characterizing fungal volatilomes. Collectively, these complementary approaches reveal that fungal VOC profiles are highly multidimensional and are influenced by both biological variability and extraction methodology. Rather than demonstrating causal relationships between specific metabolites and biological processes, the present analyses identify statistically robust candidate biomarkers that constitute promising targets for future functional, biochemical, and ecological validation studies.

### 4.4. Metabolic Pathway Analysis

The pathway enrichment analysis provides a comprehensive overview of the biochemical framework potentially associated with the fungal volatilome. The integration of KEGG and HMDB annotations allowed the detected volatile organic compounds (VOCs) to be associated with previously described metabolic pathways, facilitating the interpretation of the chemical diversity observed among the analyzed fungal species. Because these pathway assignments were generated through bioinformatic annotation rather than direct biochemical measurements, they should be interpreted as putative metabolic associations supported by existing databases and published literature rather than as experimental evidence of active metabolic fluxes. Nevertheless, the metabolite-pathway network illustrates the high degree of connectivity among primary and secondary metabolic processes that have previously been reported in fungi.

The enrichment analysis identified Fatty Acid Biosynthesis and Lipid Metabolism as the pathways containing the largest number of annotated metabolites. This observation is consistent with the predominance of lipid-derived VOCs identified throughout the dataset, including several C_8_ compounds that are widely recognized as characteristic fungal volatiles. Previous studies have shown that fatty acid oxidation and lipid turnover constitute major biochemical sources of volatile aldehydes, alcohols, ketones, and hydrocarbons in fungi [32,33]. Consequently, the pathway enrichment obtained in the present study is consistent with the well-established contribution of lipid metabolism to fungal volatilomes. However, the present work did not evaluate enzyme activities, metabolite fluxes, or gene expression; therefore, these pathway assignments should be regarded as functional hypotheses derived from metabolite annotation.

Similarly, the enrichment of Monoterpenoid Biosynthesis and Sesquiterpenoid and Triterpenoid Biosynthesis agrees with the identification of numerous terpene-derived VOCs in the analyzed samples. Terpenoids have been extensively described as important fungal secondary metabolites and have been associated in the literature with ecological interactions, including chemical communication, antimicrobial activity, defense against competitors, and interactions with plants and insects. The presence of these annotated pathways therefore is consistent with the possibility that terpenoid metabolism represents an important component of the fungal volatilome, although the activation of these biosynthetic pathways was not directly demonstrated in the present study.

The pathway analysis also associated several metabolites with xylene degradation, toluene degradation, and other aromatic compound transformation pathways [34,35]. These metabolic annotations are consistent with previous reports describing the remarkable capacity of many fungi to transform aromatic molecules through oxidative and catabolic enzymatic systems, a property that has attracted considerable interest for environmental bioremediation applications. However, these findings should be interpreted with caution. Because the analyzed fruiting bodies were collected from the Botanical Garden of the University of Caldas, an open natural environment influenced by surrounding vegetation, soil organic matter, associated microorganisms, and potential atmospheric inputs from adjacent urban areas, the detected aromatic compounds cannot be unequivocally attributed to endogenous fungal metabolism. Some of these VOCs may have originated from the surrounding substrate or environmental exposure and subsequently been adsorbed or accumulated by the fungal tissues. Consequently, the present study cannot exclude the possibility that part of the detected aromatic profile reflects environmental uptake rather than de novo fungal biosynthesis. Therefore, although the annotated pathways are compatible with metabolic capabilities previously reported for fungi, additional controlled laboratory experiments, isotope-labeling approaches, or enzymatic and transcriptomic analyses will be required to demonstrate active degradation of aromatic compounds and to distinguish endogenous metabolism from exogenous environmental contamination.

The identification of Lysine Biosynthesis among the enriched pathways further illustrates the diversity of metabolic processes represented in the volatilomic dataset. Amino acid metabolism has previously been associated with the formation of several nitrogen-containing volatile compounds and with metabolic interactions connecting primary and secondary metabolism. Although relatively few nitrogen-containing VOCs were detected compared with lipid-derived metabolites and terpenoids, the enrichment analysis suggests that amino acid metabolism may also contribute to the overall chemical diversity of the fungal volatilome.

The combined use of HMDB and KEGG substantially improved metabolite annotation by increasing the number of compounds that could be associated with known biochemical pathways. Owing to the evolutionary conservation of numerous metabolic processes among eukaryotes, databases originally developed for human metabolism can provide valuable complementary information for fungal metabolomics, particularly for non-model organisms whose metabolic pathways remain incompletely characterized. The integration of both databases therefore expanded metabolite coverage and provided additional context for the interpretation of the identified VOCs.

Overall, the pathway enrichment analysis indicates that the fungal volatilome is predominantly associated with biochemical processes related to lipid metabolism, terpenoid biosynthesis, amino acid metabolism, and aromatic compound transformation. Rather than demonstrating the activation of specific metabolic pathways, these analyses provide a bioinformatic framework for interpreting the detected VOCs and identifying metabolic processes that are consistent with previous knowledge of fungal biochemistry. Future investigations integrating transcriptomics, proteomics, enzyme activity assays, or stable-isotope labeling will be necessary to experimentally validate the biosynthetic origin and metabolic regulation of the volatile compounds identified in this study.

### 4.5. Biochemical Pathways in Fungi

The biochemical pathway annotation performed in this study provides a functional framework for interpreting the fungal volatilome and illustrates the remarkable metabolic versatility previously described for fungi. The association of the identified volatile organic compounds (VOCs) with pathways related to terpenoid biosynthesis, lipid metabolism, and aromatic compound transformation is consistent with the integration of both primary and secondary metabolism that has been widely reported in fungal systems. Because these pathway assignments were generated through metabolite annotation using KEGG and HMDB databases, they should be interpreted as putative biochemical associations supported by previous literature rather than as direct evidence of active biosynthetic pathways in the analyzed specimens.

Among the annotated metabolites, several terpenoids—including D-limonene (VOC75), caryophyllene (VOC209), longifolene (VOC207), and dauca-4(11),8-diene (VOC236)—have previously been reported to originate from the fungal mevalonate pathway through the action of terpene synthases that catalyze the cyclization of isoprenoid precursors [35]. These compounds have been associated with ecological processes such as chemical communication, antimicrobial defense, competition with neighboring microorganisms, and interactions with plants and insects. The detection of these metabolites in the analyzed fungal species is therefore consistent with previous reports describing terpenoid metabolism as an important source of fungal volatile compounds, although the activity of these biosynthetic pathways was not experimentally evaluated in the present study.

Lipid-derived VOCs constituted another major group of annotated metabolites. Compounds such as VOC250 (Heneicosane), VOC253 (2-Methyloctacosane), VOC261 (Tetratetracontane), VOC199 (4-Ethyl-2-methylhexane), VOC95 (2,3,3-Trimethyloctane), VOC28 (2,3,4-Trimethylhexane), VOC56 (3-Octanone), VOC54 (1-Octen-3-ol), and VOC61 (3-Octanol) have previously been associated with fatty acid metabolism through the elongation of acetyl-CoA and malonyl-CoA by the Fatty Acid Synthase (FAS) complex, followed by oxidative reactions that generate volatile aldehydes, alcohols, ketones, and hydrocarbons [29,30]. In particular, 1-octen-3-ol is widely recognized as the characteristic “mushroom alcohol” and represents one of the most extensively studied fungal volatile metabolites [35].

Previous biochemical investigations have demonstrated that linoleic acid may be converted into 10-hydroperoxide (10-HPOD) through dioxygenase-mediated oxidation [33]. Hydroperoxide lyases subsequently cleave this intermediate to generate 1-octen-3-ol together with 10-oxodecanoic acid, whereas additional oxidation-reduction reactions may produce 3-octanone and 3-octanol [33,34]. The identification of these metabolites in the present study is therefore consistent with lipid oxidation pathways previously described in fungi. However, no enzymatic assays, transcriptomic analyses, or isotope-labeling experiments were performed; consequently, these biosynthetic relationships should be regarded as literature-supported interpretations rather than experimentally validated metabolic processes.

Several aromatic and polycyclic compounds identified among the discriminatory VOCs—including VOC31 (*o*-Xylene), VOC32 (*m*-Xylene), VOC4 (Methyl *N*-hydroxybenzenecarboximidate), VOC234 (2,6-Di-*tert*-butyl-4-methylphenol), VOC220 (2,6-Di-*tert*-butyl-*p*-benzoquinone), and VOC41 (Cyclohexanone)—have previously been associated with the metabolism of aromatic amino acids and with secondary metabolism mediated by fungal polyketide synthases (PKSs) [31]. PKS enzymes have been reported to catalyze the sequential condensation of malonyl-CoA units to generate structurally diverse aromatic metabolites that frequently exhibit ecological and biological activities. Nevertheless, because the analyzed fruiting bodies were collected directly from the Botanical Garden of the University of Caldas under natural environmental conditions, the present study cannot unequivocally distinguish whether all detected aromatic compounds originated exclusively from endogenous fungal metabolism or whether some were influenced by the surrounding substrate, associated microorganisms, vegetation, soil organic matter, or atmospheric inputs characteristic of an open environment. Consequently, environmental adsorption of some aromatic VOCs cannot be completely excluded.

Likewise, the annotation of pathways related to xylene degradation and toluene degradation is consistent with previous reports describing the metabolic versatility of fungi for transforming aromatic compounds and environmental pollutants [36,37]. Such metabolic capabilities have attracted considerable interest because of their potential applications in environmental bioremediation. However, the present results should not be interpreted as demonstrating active degradation of these compounds by the analyzed fungi. Rather, the detected aromatic VOCs should be considered compatible with metabolic pathways previously described in fungi, while acknowledging that part of the aromatic profile may also reflect environmental exposure associated with the sampling site. Therefore, additional physiological experiments, controlled cultivation under axenic conditions, stable-isotope labeling, and transcriptomic or enzymatic analyses will be necessary to distinguish endogenous fungal metabolism from exogenous environmental contamination and to experimentally confirm these metabolic activities.

Taken together, the pathway annotation indicates that the fungal volatilome is compatible with the coordinated contribution of lipid metabolism, terpenoid biosynthesis, amino acid metabolism, and aromatic compound transformation. Rather than demonstrating the activation of specific biosynthetic routes, the present analyses provide a biologically plausible framework for interpreting the detected VOCs and identifying candidate metabolic processes that may explain the observed chemical diversity. Future studies integrating transcriptomics, proteomics, enzyme activity measurements, and stable-isotope tracing will be necessary to validate these hypotheses and to establish direct links between fungal metabolism and volatile compound production.

## 5. Conclusions

The present study provides one of the first comprehensive volatilomic characterizations of wild macromycetes collected from the Botanical Garden of the University of Caldas (Manizales, Colombia), revealing the remarkable chemical diversity of fungal volatile organic compounds (VOCs). A total of 22 discriminant metabolites were identified through multivariate analysis, including 20 annotated compounds and two unknown metabolites, highlighting the potential of fungal volatilomics for identifying candidate chemical biomarkers associated with differences in fungal diversity and extraction methodology.

The combined application of DHS-SPME, SHS-SPME, and laboratory HS-SPME coupled with GC–MS substantially expanded VOC coverage by providing complementary analytical windows into the fungal volatilome. The multivariate analyses indicated that fungal VOC profiles are highly multidimensional and are influenced by both biological variability and extraction methodology. Although laboratory HS-SPME recovered a broader range of volatile metabolites, the complementary use of in situ and laboratory-based approaches enabled a more comprehensive characterization of fungal volatile emissions under different sampling conditions.

Metabolite annotation and pathway enrichment analyses indicated that many of the detected VOCs are associated with biochemical processes previously reported for fungi, particularly lipid metabolism, fatty acid biosynthesis, terpenoid biosynthesis, amino acid metabolism, and aromatic compound transformation. Likewise, several characteristic fungal VOCs, including 1-octen-3-ol, 3-octanone, 3-octanol, D-limonene, caryophyllene, and longifolene, were identified among the most important discriminatory metabolites. These metabolic associations are based on database annotation and previously published literature and therefore should be regarded as biologically plausible hypotheses rather than experimentally validated biosynthetic pathways.

The comparison between in situ and laboratory extractions demonstrated that sampling conditions were associated with differences in the detected volatilomic profiles, emphasizing the importance of considering analytical methodology during experimental design and data interpretation. Although differences between field and laboratory profiles were observed, the present study did not directly evaluate oxidative stress biomarkers or physiological responses; consequently, the mechanisms responsible for these changes remain to be experimentally investigated.

Overall, this work establishes a robust analytical framework for fungal volatilomics by integrating complementary VOC extraction strategies, GC–MS profiling, multivariate statistics, machine-learning approaches, and metabolite pathway annotation. The resulting dataset contributes to the chemical characterization of understudied tropical macromycetes and provides a valuable resource for future investigations focused on fungal chemical ecology, chemotaxonomy, environmental monitoring, natural product discovery, and the experimental validation of the metabolic pathways inferred in the present study.

## Data Availability

The data is available in the Supplementary Information section. You can also request the information by emailing chemquantum@outlook.com.

## Supplementary Materials

The following supporting information can be downloaded at https://www.mdpi.com/article/10.3390/jof12090630/s1, Table S1. Volatile organic compounds identified in *Morchella esculenta* using HS-SPME-GC-MS under laboratory conditions. Table S2. Volatile organic compounds identified in *Scleroderma meridionale* using DHS-SPME-GC-MS in situ. Table S3. Volatile organic compounds identified in *Scleroderma meridionale* using SHS-SPME-GC-MS in situ. Table S4. Volatile organic compounds identified in *Scleroderma meridionale* using SHS-SPME-GC-MS under laboratory conditions. Table S5. Volatile organic compounds identified in *Pleurotus djamor* using DHS-SPME-GC-MS in situ. Table S6. Volatile organic compounds identified in *Pleurotus djamor* using HS-SPME-GC-MS under laboratory conditions. Table S7. Volatile organic compounds identified in *Pleurotus djamor* using SHS-SPME-GC-MS in situ. Table S8. Volatile organic compounds identified in *Laetiporus sulphureus* using HS-SPME-GC-MS under laboratory conditions. Table S9. Volatile organic compounds identified in *Laetiporus sulphureus* using SHS-SPME-GC-MS in situ. Table S10. Volatile organic compounds identified in *Polyporus tricholoma* using SHS-SPME-GC-MS in situ. Table S11. Volatile organic compounds identified in *Xylaria striata* using DHS-SPME-GC-MS in situ. Table S12. Volatile organic compounds identified in *Xylaria striata* using HS-SPME-GC-MS under laboratory conditions. Table S13. Volatile organic compounds identified in *Lentinus* sp. using HS-SPME-GC-MS under laboratory conditions. Table S14. Volatile organic compounds identified in *Lentinus* sp. using SHS-SPME-GC-MS in situ. Table S15. Volatile organic compounds identified in *Auricularia nigricans* using HS-SPME-GC-MS under laboratory conditions. Table S16. Volatile organic compounds identified in *Laetiporus* sp. using HS-SPME-GC-MS under laboratory conditions. Table S17. Volatile organic compounds identified in *Agarical* using HS-SPME-GC-MS under laboratory conditions. Table S18. Volatile organic compounds identified in *Crepidotus* sp. using HS-SPME-GC-MS under laboratory conditions. Table S19. Volatile organic compounds identified in *Cookeina* sp. using DHS-SPME-GC-MS in situ. Table S20. Volatile organic compounds identified in *Cookeina* sp. using HS-SPME-GC-MS under laboratory conditions. Table S21. Volatile organic compounds identified in *Cookeina* sp. using SHS-SPME-GC-MS in situ. Table S22. Volatile organic compounds identified in *Trametes* sp. using HS-SPME-GC-MS under laboratory conditions. Table S23. Volatile organic compounds identified in *Stereum sanguinolentum* using HS-SPME-GC-MS under laboratory conditions. Table S24. Volatile organic compounds identified in *Stereum sanguinolentum* using SHS-SPME-GC-MS in situ. Table S25. Volatile organic compounds identified in *Stereum sanguinolentum* using DHS-SPME-GC-MS in situ. Table S26. Volatile organic compounds identified in *Panus similis* using DHS-SPME-GC-MS in situ. Table S27. Volatile organic compounds identified in *Panus similis* using SHS-SPME-GC-MS in situ. Table S28. Volatile organic compounds identified in *Panus similis* using HS-SPME-GC-MS under laboratory conditions.

## Abbreviations

ANLA	National Environmental Licensing Authority
BLAST	Basic Local Alignment Search Tool
C8	Eight-carbon compounds
CARB	Carboxen
ChEBI	Chemical Entities of Biological Interest
DAD	Diode Array Detection
DDBJ	DNA Data Bank of Japan
DHS	Dynamic Headspace
DHS-SPME	Dynamic Headspace Solid-Phase Microextraction
DMAPP	Dimethylallyl Pyrophosphate
DNA	Deoxyribonucleic Acid
DOXP	1-Deoxy-D-xylulose 5-phosphate pathway
DVB	Divinylbenzene
EMBL	European Molecular Biology Laboratory
FAS	Fatty Acid Synthase
FFNSC	Flavors and Fragrances of Natural and Synthetic Compounds
FVOC	Fungal Volatile Organic Compound
FVOCs	Fungal Volatile Organic Compounds
GC	Gas Chromatography
GC–MS	Gas Chromatography–Mass Spectrometry
HCA	Hierarchical Cluster Analysis
HMDB	Human Metabolome Database
HPOD	Hydroperoxyoctadecadienoic Acid
HS	Headspace
HS-SPME	Headspace Solid-Phase Microextraction
HPLC	High-Performance Liquid Chromatography
HPLC–DAD/MS	High-Performance Liquid Chromatography with Diode Array Detection and Mass Spectrometry
HZ	Henze-Zirkler
IPP	Isopentenyl Pyrophosphate
ITS	Internal Transcribed Spacer
ITS4	Internal Transcribed Spacer 4 primer
ITS5	Internal Transcribed Spacer 5 primer
KEGG	Kyoto Encyclopedia of Genes and Genomes
MEP	Methylerythritol Phosphate pathway
MS	Mass Spectrometry
MVOC	Microbial Volatile Organic Compound
MVOCs	Microbial Volatile Organic Compounds
NCBI	National Center for Biotechnology Information
NIST	National Institute of Standards and Technology
NMR	Nuclear Magnetic Resonance
NP	Natural Product
NPs	Natural Products
PDB	Protein Data Bank
PCA	Principal Component Analysis
PC1	Principal Component 1
PC2	Principal Component 2
PCR	Polymerase Chain Reaction
PDMS	Polydimethylsiloxane
PKS	Polyketide Synthase
QA	Quality Assurance
QC	Quality Control
QA/QC	Quality Assurance and Quality Control
RT	Retention Time
SHS	Static Headspace
SHS-SPME	Static Headspace Solid-Phase Microextraction
SI	Similarity Index
SPME	Solid-Phase Microextraction
SQC	System Quality Control
SVM	Support Vector Machine
VIP	Variable Importance Plot
VIPs	Variable Importance Variables/Important Differentiating Variables
VOC	Volatile Organic Compound
VOCs	Volatile Organic Compounds
