# Supplementary material for: Volatilomic Study of the Chemodiversity of Metabolites in Wild Macromycetes of the Lower Montane Humid Forest
Source: J Fungi (Basel). 2026 Aug 23;12(9):630. doi: 10.3390/jof12090630 (PMC13608634; doi:10.3390/jof12090630)
Supplement: Supplementary file 1 [file jof-12-00630-s001.zip › jof-4416704-supplementary.pdf]

**Supplementary Information**

**Table S1.** Volatile organic compounds identified in *Morchella esculenta* using HS-SPME-GC-MS under laboratory conditions.

| *Morchella esculenta* SHS-SPME VCQ06 | | | | | | |
|---|---|---|---|---|---|---|
| **RT** | **Name** | **Common name** | **Identification level** | **Area** | **SI** | **S/N** |
| **1.670** | Hexane | Hexane | 3 | 20986738 | 97 | 875.69 |
| **1.800** | Cyclopentane, methyl- | Methylcyclopentane | 3 | 4228159 | 98 | 101.40 |
| **2.090** | Heptane | Heptane | 3 | 870439 | 96 | 46.77 |
| **2.725** | 3-Hexanol | 3-Hexanol | 3 | 836851 | 98 | 1371.65 |
| **3.740** | Oxime-, methoxy-phenyl-_ | methyl N-hydroxybenzenecarboximidate | 3 | 83596 | 90 | 26.25 |
| **8.135** | Butylated Hydroxytoluene | 2,6-Di-tert-butyl-4-methylphenol | 3 | 229766 | 94 | 431.70 |
| **10.625** | RT10.625 | RT10.625 | 3 | 50920 | 88 | 10.09 |

**SI =** Similarity index, **S/N =** signal-to-noise ratio

**Table S2.** Volatile organic compounds identified in *Scleroderma meridionale* using DHS-SPME-GC-MS in situ.

| *Scleroderma meridionale* DHS-SPME VCQ07 | | | | | | |
|---|---|---|---|---|---|---|
| **RT** | **Name** | **Common name** | **Identification level** | **Area** | **SI** | **S/N** |
| **4.805** | 1,3-Octadiene | 1,3-Octadiene | 3 | 322449 | 93 | 193.39 |
| **5.255** | Hexane, 2,3,4-trimethyl- | 2,3,4-trimethylhexane | 3 | 56895 | 87 | 18.19 |
| **5.405** | o-Xylene | o-Xylene | 3 | 73428 | 93 | 3,53 |
| **5.900** | RT5.900 | - | 3 | 38105 | - | 0,64 |
| **8.140** | Decane, 2,2-dimethyl- | 2,2-dimethyldecane | 3 | 36954 | 83 | 9,86 |
| **9.115** | 2-Cyclohexen-1-ol, 1-methyl-4-(1-methylethyl)-, cis- | 1-methyl-4-propan-2-ylcyclohex-2-en-1-ol | 3 | 189466 | 83 | 12,16 |
| **9.795** | RT9.795 | | 3 | 159133 | - | 2,01 |
| **10.810** | Undecane | Undecane | 3 | 51696 | 98 | 5,84 |
| **11.240** | 5-Undecene, 9-methyl-, (Z)- | (Z)-9-methylundec-5-ene | 3 | 303270 | 90 | 9,8 |
| **12.420** | 2-Undecanethiol, 2-methyl- | 2-Methylundecane-2-thiol | 3 | 131676 | 85 | 4,3 |
| **12.525** | Undecane, 3-methyl- | 3-methylundecane | 3 | 234581 | 96 | 12,78 |
| **12.905** | 5-Undecene, 3-methyl-, (E)- | (5E)-3-Methyl-5-undecene | 3 | 79951 | 89 | 6,56 |
| **13.035** | RT13.035 | | 3 | 48651 | - | 0,2 |
| **13.235** | Tridecane | Tridecane | 3 | 1877922 | 97 | 80,63 |
| **13.545** | Decane, 5-ethyl-5-methyl- | 5-ethyl-5-methyldecane | 3 | 395433 | 94 | 4,63 |
| **13.895** | 1-Hexene, 2,4,4-triethyl- | 2,4,4-Triethyl-1-hexene | 3 | 208652 | 80 | 1,29 |
| **14.245** | Cyclopentane, pentyl- | Pentylcyclopentane | 3 | 224021 | 91 | 6,14 |
| **14.515** | RT14.515 | | 3 | 61178 | - | 0,8 |
| **14.610** | 5-Octadecene, (E)- | (E)-5-Octadecene | 3 | 236864 | 89 | 3,18 |
| **14.765** | 10-Methylnonadecane | 10-Methylnonadecane | 3 | 135567 | 88 | 0,99 |
| **15.700** | 1-Nonene, 4,6,8-trimethyl- | 4,6,8-trimethyl-1-nonene | 3 | 422280 | 88 | 8,1 |
| **15.785** | Decane, 2,3,5-trimethyl- | 2,3,5-Trimethyldecane | 3 | 192594 | 84 | 1,94 |
| **16.045** | Cyclopropane, 1-methyl-1-(1-methylethyl)-2-nonyl- | 1-methyl-1-(1-methylethyl)-2-nonylcyclopropane | 3 | 224571 | 80 | 1,05 |
| **16.360** | Nonane, 3-methyl-5-propyl- | 3-Methyl-5-propylnonane | 3 | 211394 | 94 | 2,29 |
| **16.445** | 3,5-Dimethyldodecane | 3,5-Dimethyldodecane | 3 | 116357 | 92 | 2,38 |
| **16.605** | Decane, 3-ethyl-3-methyl- | 3-Ethyl-3-methyldecane | 3 | 223718 | 95 | 3,24 |
| **17.150** | Tridecane, 3-methyl- | 3-Methyltridecane | 3 | 546701 | 95 | 4,95 |
| **17.515** | Hexane, 4-ethyl-2-methyl- | 4-Ethyl-2-methylhexane | 3 | 181571 | 89 | 2,41 |
| **17.880** | RT17.880 | - | 3 | 270867 | - | 7 |
| **18.045** | Longifolene | Longifolene | 3 | 277729 | 96 | 9,93 |

| *Scleroderma meridionale* DHS-SPME VCQ07 | | | | | | |
|---|---|---|---|---|---|---|
| **RT** | **Name** | **Common name** | **Identification level** | **Area** | **SI** | **S/N** |
| **18.820** | Methoxyacetic acid, 3-tridecyl ester | 1-Ethylundecyl methoxyacetate | 3 | 226864 | 91 | 6,08 |
| **19.890** | RT19.890 | | 3 | 181226 | 83 | 22,68 |
| **20.200** | Butylated Hydroxytoluene | 2,6-Di-tert-butyl-4-methylphenol | 3 | 400379 | 98 | 134,33 |
| **21.895** | Pentanoic acid, 2,2,4-trimethyl-3-carboxyisopropyl, isobutyl ester | 1-Isobutyl 4-isopropyl 3-isopropyl-2,2-dimethylsuccinate | 3 | 277951 | 95 | 53,25 |
| **36.220** | Heneicosane | Heneicosane | 3 | 125043 | 90 | 17,16 |

**Table S3.** Volatile organic compounds identified in *Scleroderma meridionale* using SHS-SPME-GC-MS in situ.

| *Scleroderma meridionale* SHS-SPME VCQ07 | | | | | | |
|---|---|---|---|---|---|---|
| **RT** | **Name** | **Common name** | **Identification level** | **Area** | **SI** | **S/N** |
| **1.859** | n-Hexane | Hexane | 3 | 201563 | 94 | 163,09 |
| **3.495** | Toluene | Toluene | 3 | 57509 | 93 | 61,94 |
| **3.834** | 1-Octene | 1-Octene | 3 | 3202891 | 99 | 867,86 |
| **4.445** | 1,3-Octadiene | 1,3-Octadiene | 3 | 27112272 | 100 | 431,54 |
| **5.200** | RT5.200 | - | 3 | 321939 | - | 266,76 |
| **5.344** | Benzene, 1,3-dimethyl- | m-xylene | 3 | 1151807 | 99 | 3,52 |
| **5.851** | o-Xylene | o-Xylene | 3 | 509774 | 99 | 0,9 |
| **6.270** | RT6.270 | - | 3 | 4683596 | - | 2532,74 |
| **7.370** | RT7.370 | - | 3 | 442769 | - | 498,18 |
| **8.004** | 3-Octanone | 3-Octanone | 3 | 2168608 | 99 | 92,47 |
| **9.074** | D-Limonene | D-Limonene | 3 | 726196 | 96 | 23,08 |
| **9.194** | Decane, 2,9-dimethyl- | 2,9-Dimethyldecane | 3 | 366857 | 99 | 5,83 |
| **9.282** | trans-. beta. -Ocimene | (E)-beta-ocimene | 3 | 216609 | 94 | 9,8 |
| **10.221** | Decane, 3,4-dimethyl- | 3,4-dimethyldecane | 3 | 270900 | 94 | 2,51 |
| **10.533** | 3-Undecene, 6-methyl-, (E)- | (3E)-6-Methyl-3-undecene | 3 | 1119324 | 99 | 16,33 |
| **11.949** | (+)-2-Bornanone | Camphor | 3 | 376418 | 94 | 39,32 |
| **12.419** | RT12.419 | - | 3 | 1551565 | - | 42,3 |
| **12.505** | Dodecane, 2,2,11,11-tetramethyl- | 2,2,11,11-tetramethyldodecane | 3 | 601476 | 99 | 21,32 |
| **14.624** | Hexadecane | Hexadecane | 3 | 106897 | 97 | 3,09 |
| **17.379** | Copaene | Copaene | 3 | 87664 | 99 | 24,99 |
| **19.530** | RT19.530 | - | 3 | 1007134 | - | 58,51 |
| **19.640** | Germacrene D | Germacrene D | 3 | 56304 | 98 | 3,52 |
| **20.220** | Butylated Hydroxytoluene | Butylated Hydroxytoluene | 3 | 17124779 | 99 | 1188,13 |

**Table S4.** Volatile organic compounds identified in *Scleroderma meridionale* using SHS-SPME-GC-MS under laboratory conditions.

| *Scleroderma meridionale* SHS-SPME VCQ07 | | | | | | |
|---|---|---|---|---|---|---|
| **RT** | **Name** | **Common name** | **Identification level** | **Area** | **SI** | **S/N** |
| **1.847** | n-Hexane | Hexane | 3 | 158659 | 94 | 23,05 |
| **5.167** | Ethylbenzene | Ethylbenzene | 3 | 1323783 | 95 | 1715,4 |
| **5.334** | Benzene, 1,3-dimethyl- | m-xylene | 3 | 1515762 | 99 | 3,86 |
| **5.802** | 1,3,5,7-Cyclooctatetraene | Cyclooctatetraene | 3 | 2830806 | 94 | 49,57 |
| **6.227** | Oxime-, methoxy-phenyl-_ | methyl N-hydroxybenzenecarboximidate | 3 | 4568988 | 100 | 2386,67 |
| **6.767** | . alpha. -Pinene | alpha-pinene | 3 | 411753 | 99 | 226,43 |
| **7.118** | Bicyclo[2.2.1]heptane, 2,2-dimethyl-3-methylene-, (1S)- | L-Camphene | 3 | 176114 | 93 | 20,71 |
| **7.365** | RT7.365 | - | 3 | 379950 | 96 | 415,17 |
| **7.790** | Bicyclo[3.1.1]heptane, 6,6-dimethyl-2-methylene-, (1S)- | beta-pinene | 3 | 265053 | 98 | 325,95 |
| **8.104** | Heptane, 2,2,4,6,6-pentamethyl- | 2,2,4,6,6-pentamethylheptane | 3 | 162208 | 99 | 68,44 |
| **8.712** | 2-Propanol, 1-(2-methoxypropoxy)- | 1-(2-methoxypropoxy)-2-propanol | 3 | 269477 | 96 | 43,65 |
| **8.957** | p-Cymene | p-Cymene | 3 | 3562411 | 99 | 1638,78 |
| **9.809** | . gamma. -Terpinene | gamma-terpinene | 3 | 1948745 | 90 | 476,17 |
| **10.214** | Pentane, 3,3-dimethyl- | 3,3-dimethylpentane | 3 | 470533 | 88 | 1,25 |
| **10.561** | Cyclopentane, 1-butyl-2-propyl- | 1-butyl-2-propylcyclopentane | 3 | 967266 | 94 | 1,57 |
| **10.692** | Octane, 6-ethyl-2-methyl- | 6-Ethyl-2-methyloctane | 3 | 283127 | 92 | 0,9 |
| **10.808** | Undecane | Undecane | 3 | 8624254 | 100 | 72,73 |
| **11.240** | 5-Undecene, 9-methyl-, (Z)- | 6-Ethyl-2-methyloctane | 3 | 3364569 | 99 | 9,24 |
| **11.668** | Cyclohexane, pentyl- | pentylcyclohexane | 3 | 720634 | 93 | 5,48 |
| **11.941** | Bicyclo[2.2.1]heptan-2-one, 1,7,7-trimethyl-, (1S)- | camphor | 3 | 3321671 | 98 | 107,04 |
| **12.425** | 3-Octene, 2,2-dimethyl- | (3E)-2,2-Dimethyl-3-octene | 3 | 836676 | 94 | 4,63 |
| **12.527** | Undecane, 3-methyl- | 3-methylundecane | 3 | 1724837 | 96 | 19,99 |
| **12.765** | 3-Cyclohexen-1-ol, 4-methyl-1-(1-methylethyl)-, (R)- | (-)-4-Terpineol | 3 | 152132 | 93 | 0,15 |
| **12.888** | Undecane, 3-methylene- | 2-Ethyl-1-decene | 3 | 682916 | 99 | 7,34 |
| **13.032** | Cyclotetradecane | Cyclotetradecane | 3 | 665965 | 89 | 0,81 |
| **13.238** | Tridecane | Tridecane | 3 | 7682229 | 99 | 105,27 |

| *Scleroderma meridionale* SHS-SPME VCQ07 | | | | | | |
|---|---|---|---|---|---|---|
| **RT** | **Name** | **Common name** | **Identification level** | **Area** | **SI** | **S/N** |
| **13.560** | Decane, 5-ethyl-5-methyl- | 5-ethyl-5-methyldecane | 3 | 1382803 | 95 | 6,94 |
| **15.704** | 1-Nonene, 4,6,8-trimethyl- | 4,6,8-trimethylnon-1-ene | 3 | 682113 | 97 | 4,72 |
| **17.137** | Undecane, 2,10-dimethyl- | 2,10-Dimethylundecane | 3 | 215489 | 98 | 8,75 |
| **17.485** | RT17.485 | - | 3 | 940985 | - | 37,79 |
| **18.824** | 2-Hexyl-1-octanol | 2-Hexyl-1-octanol | 3 | 427162 | 99 | 6,25 |
| **21.870** | RT21.870 | - | 3 | 1448837 | - | 257,65 |
| **37.411** | Heneicosane | Heneicosane | 3 | 222941 | 99 | 9,27 |
| **40.212** | Squalene | Squalene | 3 | 300791 | 98 | 45,41 |

**Table S5.** Volatile organic compounds identified in *Pleurotus djamor* using DHS-SPME-GC-MS in situ.

| *Pleurotus djamor* DHS-SPME VCQ09 | | | | | | |
|---|---|---|---|---|---|---|
| **RT** | **Name** | **Common name** | **Identification level** | **Area** | **SI** | **S/N** |
| **3.212** | Cyclohexane, methyl- | methylcyclohexane | 3 | 219702 | 99 | 188,2 |
| **5.455** | RT5.455 | - | 3 | 15434 | - | 3,42 |
| **5.925** | Cyclohexanone | cyclohexanone | 3 | 153411 | 98 | 251,76 |
| **6.845** | (1R)-2,6,6-Trimethylbicyclo [3.1.1] hept-2-ene | alpha-pinene | 3 | 151882 | 99 | 148,83 |
| **7.840** | . beta. -Pinene | beta-pinene | 3 | 52668 | 97 | 54,3 |
| **8.163** | . beta. -Myrcene | beta-Myrcene | 3 | 1008646 | 100 | 942,98 |
| **8.925** | Benzene, 1,2,4-trimethyl- | 1,2,4-trimethylbenzene | 3 | 138739 | 99 | 196,55 |
| **9.081** | D-Limonene | D-Limonene | 3 | 183457 | 99 | 130,43 |
| **9.249** | Indane | Indane | 3 | 40736 | 91 | 17 |
| **9.626** | 2-Tolyloxirane | 2-(2-Methylphenyl) oxirane | 3 | 120182 | 97 | 108,03 |
| **9.714** | Benzene, (1-methylpropyl)- | 2-Phenylbutane | 3 | 42586 | 84 | 50,3 |
| **9.801** | Benzene, 2-ethyl-1,3-dimethyl- | 2-Ethyl-m-xylene | 3 | 140627 | 95 | 6,4 |
| **10.011** | Benzene, 1-methyl-4-propyl- | p-Propyltoluene | 3 | 43778 | 94 | 3,49 |
| **10.303** | RT10.303 | - | 3 | 176313 | - | 7,39 |
| **10.460** | Benzene, 1-ethyl-2,3-dimethyl- | 3-Ethyl-o-xylene | 3 | 178758 | 99 | 27,38 |
| **10.981** | Benzene, 4-ethyl-1,2-dimethyl- | 4-Ethyl-o-xylene | 3 | 53359 | 95 | 1,39 |
| **11.218** | RT11.218 | - | 3 | 116277 | - | 65,64 |
| **11.322** | Benzene, 1,2,4,5-tetramethyl- | Durene | 3 | 171224 | 99 | 168,46 |
| **11.788** | 1H-Indene, 2,3-dihydro-4-methyl- | 4-methyl-2,3-dihydro-1H-indene | 3 | 21621 | 89 | 16,79 |
| **12.104** | 1,3-Cyclopentadiene, 1,2,3,4-tetramethyl-5-methylene- | 1,2,3,4-Tetramethylfulvene | 3 | 91374 | 88 | 30,05 |
| **18.324** | Caryophyllene | beta-caryophyllene | 3 | 22138 | 88 | 5,18 |
| **20.177** | Butylated Hydroxytoluene | 2,6-Di-tert-butyl-4-methylphenol | 3 | 147535 | 98 | 144,75 |
| **36.066** | Heptadecane, 2,6,10,15-tetramethyl- | 2,6,10,15-tetramethylheptadecane | 3 | 21812 | 93 | 18,48 |
| **38.579** | Heneicosane | Heneicosane | 3 | 48929 | 96 | 20,71 |
| **38.760** | RT38.760 | - | 3 | 37571 | - | 26,76 |
| **39.771** | Hexacosane | Hexacosane | 3 | 55979 | 96 | 22,38 |

**Table S6.** Volatile organic compounds identified in *Pleurotus djamor* using HS-SPME-GC-MS under laboratory conditions.

| *Pleurotus djamor* HS - SPME VCQ09 | | | | | | |
|---|---|---|---|---|---|---|
| **RT** | **Name** | **Common name** | **Identification level** | **Area** | **SI** | **S/N** |
| **3.208** | Cyclohexane, methyl- | methylcyclohexane | 3 | 204775 | 99 | 188,2 |
| **5.927** | Cyclohexanone | cyclohexanone | 3 | 153011 | 98 | 251,76 |
| **6.843** | (1R)-2,6,6-Trimethylbicyclo [3.1.1] hept-2-ene | alpha-pinene | 3 | 150257 | 99 | 148,83 |
| **7.843** | . beta. -Pinene | beta-pinene | 3 | 52668 | 97 | 54,3 |
| **8.161** | . beta. -Myrcene | beta-myrcene | 3 | 1008434 | 100 | 942,98 |
| **8.928** | Benzene, 1,2,4-trimethyl- | 1,2,4-trimethylbenzene | 3 | 139314 | 99 | 196,55 |
| **9.084** | D-Limonene | D-Limonene | 3 | 183807 | 99 | 130,43 |
| **9.253** | Indane | Indane | 3 | 40862 | 91 | 17 |
| **9.617** | 2-Tolyloxirane | 2-Tolyloxirane | 3 | 118877 | 97 | 108,03 |
| **9.725** | RT9.725 | - | 3 | 46003 | - | 50,3 |
| **9.797** | Benzene, 2-ethyl-1,3-dimethyl- | 2-Ethyl-m-xylene | 3 | 132131 | 94 | 6,4 |
| **10.017** | Benzene, 1-methyl-4-propyl- | p-Propyltoluene | 3 | 34087 | 93 | 3,49 |
| **10.284** | Benzene, 2-ethyl-1,4-dimethyl- | 2-Ethyl-p-xylene | 3 | 175728 | 90 | 7,39 |
| **10.463** | Benzene, 1-ethyl-2,3-dimethyl- | 3-Ethyl-o-xylene | 3 | 178481 | 99 | 27,38 |
| **10.987** | Benzene, 4-ethyl-1,2-dimethyl- | 4-Ethyl-o-xylene | 3 | 53131 | 95 | 1,39 |
| **11.219** | Benzene, 1,2,4,5-tetramethyl- | Durene | 3 | 116277 | 99 | 65,64 |
| **11.325** | RT11.325 | - | 3 | 171224 | - | 168,46 |
| **11.784** | 1H-Indene, 2,3-dihydro-4-methyl- | 4-methylindan | 3 | 13690 | 88 | 16,79 |
| **12.097** | 1,3-Cyclopentadiene, 1,2,3,4-tetramethyl-5-methylene | 1,2,3,4-Tetramethylfulvene | 3 | 91374 | 88 | 30,05 |
| **12.656** | RT12.656 | - | 3 | 10839 | - | 4,87 |
| **17.493** | RT17.493 | - | 3 | 10579 | - | 9,01 |
| **18.323** | Caryophyllene | beta-caryophyllene | 3 | 20069 | 88 | 5,18 |
| **20.179** | Butylated Hydroxytoluene | Butylhydroxytoluene | 3 | 146927 | 99 | 144,75 |
| **34.738** | RT34.738 | - | 3 | 10317 | - | 10,68 |
| **35.153** | RT35.153 | | 3 | 52127 | | |
| **36.065** | 2-methyloctacosane | 2-methyloctacosane | 3 | 20623 | 90 | 18,48 |
| **37.375** | RT37.375 | | 3 | 82292 | | |
| **37.428** | RT37.428 | - | 3 | 91687 | 74 | 31,91 |
| **38.581** | Heneicosane | Heneicosane | 3 | 48606 | 96 | 20,71 |
| **38.761** | RT38.761 | - | 3 | 36436 | - | 26,76 |
| **39.085** | 2-methyloctacosane | Isononacosane | 3 | 50736 | 85 | 0,61 |

| *Pleurotus djamor* HS - SPME VCQ09 | | | | | | |
|---|---|---|---|---|---|---|
| **RT** | **Name** | **Common name** | **Identification level** | **Area** | **SI** | **S/N** |
| **39.774** | Hexacosane | Hexacosane | 3 | 54595 | 96 | 22,38 |
| **40.559** | Tetratetracontane | n-Tetratetracontane | 3 | 145171 | 99 | 127,05 |

**Table S7.** Volatile organic compounds identified in *Pleurotus djamor* using SHS-SPME-GC-MS in situ.

| *Pleurotus djamor* SHS - SPME VCQ09 | | | | | | |
|---|---|---|---|---|---|---|
| **RT** | **Name** | **Common name** | **Identification level** | **Area** | **SI** | **S/N** |
| **5.429** | RT5.429 | - | 3 | 20672 | - | 11,01 |
| **6.796** | (1R)-2,6,6-Trimethylbicyclo [3.1.1] hept-2-ene | alpha-pinene | 3 | 63054 | 95 | 46,14 |
| **7.808** | . beta. -Pinene | beta-pinene | 3 | 66738 | 94 | 73,93 |
| **8.024** | 3-Octanone | 3-octanone | 3 | 55468 | 94 | 75 |
| **8.960** | o-Cymene | o-cymene | 3 | 20540 | 80 | 12,16 |
| **9.134** | Eucalyptol | eucalyptol | 3 | 83335 | 96 | 16,43 |
| **9.809** | . gamma. -Terpinene | gamma-terpinene | 3 | 17974 | 89 | 14,12 |
| **12.874** | 2-Methylisoborneol | 2-methylisoborneol | 3 | 20711 | 90 | 15,51 |
| **17.254** | 1H-Cyclopropa[a]naphthalene, 1a,2,6,7,7a,7b-hexahydro-1,1,7,7a | 1,2,9,10-tetradehydroaristolane | 3 | 20587 | 91 | 9,1 |
| **21.187** | Nerolidol | nerolidol | 3 | 94693 | 98 | 44,79 |
| **35.135** | Isopropyl tetradecyl ether | isopropyl tetradecyl ether | 3 | 78780 | 78 | 18,94 |
| **40.751** | Tetratetracontane | n-tetratetracontane | 3 | 118433 | 97 | 44,07 |

**Table S8.** Volatile organic compounds identified in *Laetiporus sulphureus* using HS-SPME-GC-MS under laboratory conditions.

| *Laetiporus sulphureus* HS - SPME VCQ12 | | | | | | |
|---|---|---|---|---|---|---|
| **RT** | **Name** | **Common name** | **Identification level** | **Area** | **SI** | **S/N** |
| **3.919** | RT3.919 | - | 3 | 59141 | - | 76,65 |
| **4.385** | RT4.385 | - | 3 | 25138 | - | 16,27 |
| **4.618** | RT4.618 | - | 3 | 50967 | - | |
| **4.873** | RT4.873 | - | 3 | 25727 | - | 15,38 |
| **5.656** | RT5.656 | - | 3 | 747097 | - | 687,31 |
| **5.830** | RT5.830 | - | 3 | 54027 | - | 0,93 |
| **6.763** | . alpha. -Pinene | alpha-pinene | 3 | 16715 | 82 | 22,22 |
| **7.752** | RT7.752 | - | 3 | 9432 | - | 1,19 |
| **7.869** | 1-Octen-3-ol | 1-octen-3-ol | 3 | 127758 | 95 | 41,08 |
| **7.998** | 3-Heptanone, 5-methyl- | 5-methylheptan-3-one | 3 | 287749 | 98 | 74,39 |
| **8.115** | . beta. -Myrcene | beta-myrcene | 3 | 49428 | 91 | 18,9 |
| **8.243** | 3-Octanol | 3-octanol | 3 | 190953 | 97 | 154,14 |
| **8.919** | RT8.919 | - | 3 | 28383 | - | 13,74 |
| **9.069** | D-Limonene | d-limonene | 3 | 1932815 | 100 | 1137,19 |
| **9.599** | RT9.599 | - | 3 | 17966 | | |
| **10.312** | RT10.312 | - | 3 | 15758 | - | 1,08 |
| **10.475** | RT10.475 | - | 3 | 26264 | - | 4,53 |
| **10.833** | RT10.833 | - | 3 | 19205 | - | 12,48 |
| **11.213** | RT11.213 | - | 3 | 18266 | - | 16,3 |
| **11.333** | 1,3-Cyclopentadiene, 1,2,3,4-tetramethyl- | 1,2,3,4-tetramethylcyclopenta-1,3-diene | 3 | 13029 | 83 | 21,23 |
| **12.177** | RT12.177 | - | 3 | 103514 | - | 83,1 |
| **16.755** | . alpha. -Cubebene | alpha-cubebene | 3 | 175789 | 100 | 106,89 |
| **17.432** | 2H-2,4a-Methanonaphthalene, 1,3,4,5,6,7-hexahydro | isolongifolene | 3 | 445756 | 95 | 109,9 |
| **17.673** | Alloaromadendrene | alloaromadendrene | 3 | 178791 | 81 | 38,64 |
| **18.338** | Bicyclo[7.2.0]undec-4-ene, 4,11,11-trimethyl-8-methylene-,[1R- | caryophyllene | 3 | 72166 | 82 | 9,44 |
| **18.440** | RT18.440 | - | 3 | 48842 | - | 6,15 |
| **18.610** | trans-. alpha. -Bergamotene | trans-alpha-bergamotene | 3 | 30166 | 93 | 4,61 |
| **18.869** | RT18.869 | - | 3 | 24480 | - | 8,69 |

| *Laetiporus sulphureus* HS - SPME VCQ12 | | | | | | |
|---|---|---|---|---|---|---|
| **RT** | **Name** | **Common name** | **Identification level** | **Area** | **SI** | **S/N** |
| **19.375** | (4S,4aR,6R)-4,4a-Dimethyl-6-(prop-1-en- | capsidiol | 3 | 37852 | 90 | 6,12 |
| **19.477** | 1H-Cyclopropa[a]naphthalene, decahydro-1,1,3a | 1,1,3a-trimethyl-7-methylenedecahydro-1h-cyclopropa[a]naphthalene | 3 | 206138 | 99 | 29,58 |
| **19.650** | (E)-. beta. -Famesene | (e)-beta-farnesene | 3 | 215582 | 99 | 59,02 |
| **19.738** | Naphthalene, 1,2,3,5,6,7,8,8a-octahydro-1,8a-dimethyl | valencene | 3 | 58092 | 96 | 2,6 |
| **19.898** | Spiro [5.5] undec-2-ene, 3,7,7-trimethyl-11-methylene | beta-chamigrene | 3 | 94697 | 82 | 1,16 |
| **20.183** | Butylated Hydroxytoluene | butylhydroxytoluene | 3 | 259567 | 98 | 207,87 |
| **20.440** | 1-Isopropyl-4,7-dimethyl-1,2,3,5,6,8a-hexahydronaphthalene | delta-amorphene | 3 | 66742 | 98 | 5,27 |
| **20.591** | (3aS,8aS)-6,8a-Dimethyl-3-(propan-2-ylidene)-1,2,3,3a,4,5,8,8a | dauca-4(11),8-diene | 3 | 308954 | 100 | 171,13 |
| **21.904** | RT21.904 | - | 3 | 15108 | - | 4,58 |
| **37.009** | RT37.009 | - | 3 | 28018 | - | 12,03 |
| **38.779** | RT38.779 | - | 3 | 13918 | - | 10,82 |
| **40.382** | RT40.382 | - | 3 | 13746 | - | 9,13 |
| **40.708** | RT40.708 | - | 3 | 14049 | - | 7,69 |

**Table S9.** Volatile organic compounds identified in *Laetiporus sulphureus* using SHS-SPME-GC-MS in situ.

| *Laetiporus sulphureus* SHS - SPME VCQ12 | | | | | | |
|---|---|---|---|---|---|---|
| **RT** | **Name** | **Common name** | **Identification level** | **Area** | **SI** | **S/N** |
| **8.134** | . beta. -Myrcene | beta-Myrcene | 3 | 197848 | 98 | 100,39 |
| **8.331** | RT8.331 | - | 3 | 24551 | - | 9,96 |
| **9.074** | Cyclobutane, 1,2-bis(1-methylethenyl)-, trans- | 1,2-Diisopropenylcyclobutane | 3 | 45761 | 86 | 12,98 |
| **17.436** | 3-Isopropyl-6,8a-dimethyl-1,2,4,5,8,8a-hexahydroazulene | Daucene | 3 | 27378 | 92 | 15,47 |
| **20.190** | Butylated Hydroxytoluene | Butylhydroxytoluene | 3 | 44509 | 93 | 29,38 |
| **20.595** | (3aS,8aS)-6,8a-Dimethyl-3-(propan-2-ylidene)-1,2,3,3a,4,5,8,8a | Dauca-4(11),8-diene | 3 | 33685 | 92 | 18,38 |

**Table S10.** Volatile organic compounds identified in *Polyporus tricholoma* using SHS-SPME-GC-MS in situ.

| *Polyporus tricholoma* SHS-SPME VCQ13 | | | | | | |
|---|---|---|---|---|---|---|
| **RT** | **Name** | **Common name** | **Identification level** | **Area** | **SI** | **S/N** |
| **6.787** | (1R)-2,6,6-Trimethylbicyclo [3.1.1] hept-2-ene | alpha-pinene | 3 | 63054 | 95 | 46.14 |
| **7.802** | . beta. -Pinene | beta-pinene | 3 | 66738 | 94 | 73.93 |
| **8.016** | 3-Octanone | 3-Octanone | 3 | 55468 | 94 | 75.00 |
| **8.962** | o-Cymene | o-Cymene | 3 | 20540 | 80 | 12.16 |
| **9.126** | Eucalyptol | Eucalyptol | 3 | 83335 | 96 | 16.43 |
| **9.809** | . gamma. -Terpinene | gamma-terpinene | 3 | 17974 | 89 | 14.12 |
| **12.869** | 2-Methylisoborneol | 2-methylisoborneol | 3 | 20711 | 90 | 15.51 |
| **17.255** | 1H-Cyclopropa[a]naphthalene, 1a,2,6,7,7a,7b-hexahydro-1,1,7,7a-tetramethyl-, [1aR-(1a. alpha.,7. alpha.,7a. alpha.,7b. alpha.)]- | 1,2,9,10-Tetradehydroaristolane | 3 | 20587 | 91 | 9.10 |
| **21.182** | Nerolidol | Nerolidol | 3 | 94693 | 98 | 44.79 |
| **40.744** | Tetratetracontane | Tetratetracontane | 3 | 118433 | 97 | 44.07 |

**Table S11.** Volatile organic compounds identified in *Xylaria striata* using DHS-SPME-GC-MS in situ.

| *Xylaria striata* DHS - SPME VCQ14 | | | | | | |
|---|---|---|---|---|---|---|
| **RT** | **Name** | **Common name** | **Identification level** | **Area** | **SI** | **S/N** |
| **3.501** | RT3.501 | - | 3 | 193293 | - | 17,48 |
| **3.916** | RT3.916 | - | 3 | 98213 | - | 66,15 |
| **4.577** | 2-Pentenal, 2-methyl- | 2-methyl-2-pentenal | 3 | 216118 | 99 | 177,06 |
| **4.790** | RT4.790 | - | 3 | 43986 | - | 41,46 |
| **5.343** | o-Xylene | o-xylene | 3 | 21176 | 87 | 5,56 |
| **5.652** | RT5.652 | - | 3 | 918440 | - | 901,34 |
| **5.811** | Styrene | styrene | 3 | 113020 | 86 | 29,22 |
| **7.883** | RT7.883 | - | 3 | 36736 | - | 29,4 |
| **8.999** | 3-Octanone | 3-octanone | 3 | 32717 | 86 | 18,79 |
| **8.120** | RT8.120 | - | 3 | 29634 | - | 14,36 |
| **9.054** | D-Limonene | D-limonene | 3 | 632348 | 100 | 369,74 |
| **9.770** | RT9.770 | - | 3 | 14547 | - | |
| **10.818** | RT10.818 | - | 3 | 13395 | - | |
| **11.206** | RT11.206 | - | 3 | 19206 | - | |
| **11.930** | Bicyclo[2.2.1]heptan-2-one, 1,7,7-trimethyl-, (1S)- | camphor | 3 | 12655 | 87 | 6,08 |
| **12.165** | RT12.165 | - | 3 | 46776 | - | 53,95 |
| **12.400** | RT12.400 | - | 3 | 14481 | - | 5,23 |
| **13.229** | RT13.229 | - | 3 | 9755 | - | 16,18 |
| **17.490** | Hexane, 4-ethyl-2-methyl- | 4-ethyl-2-methylhexane | 3 | 21222 | 86 | 19,63 |
| **18.324** | Caryophyllene | caryophyllene | 3 | 49624 | 96 | 12,17 |
| **20.178** | Butylated Hydroxytoluene | butylhydroxytoluene | 3 | 231336 | 99 | 210,53 |
| **35.152** | RT35.152 | - | 3 | 21534 | - | 7,93 |
| **37.015** | RT37.015 | - | 3 | 16377 | - | 14,3 |
| **38.777** | RT38.777 | - | 3 | 18935 | - | 11,3 |
| **40.371** | RT40.371 | - | 3 | 19408 | - | 9,09 |

**Table S12.** Volatile organic compounds identified in *Xylaria striata* using HS-SPME-GC-MS under laboratory conditions.

| *Xylaria striata* HS - SPME VCQ14 | | | | | | |
|---|---|---|---|---|---|---|
| **RT** | **Name** | **Common name** | **Identification level** | **Area** | **SI** | **S/N** |
| **3.044** | Pentane, 2,2,3,4-tetramethyl- | 2,2,3,4-tetramethylpentane | 3 | 136537 | 99 | 37,3 |
| **3.188** | Cyclohexane, methyl- | methylcyclohexane | 3 | 2767650 | 100 | 6067,69 |
| **3.284** | Cyclopentane, ethyl- | ethylcyclopentane | 3 | 364049 | 88 | 65,4 |
| **5.311** | Ethylbenzene | ethylbenzene | 3 | 65992 | 88 | 72,79 |
| **5.465** | Benzene, 1,3-dimethyl- | m-xylene | 3 | 289894 | 97 | 26,81 |
| **5.941** | Cyclohexanone | cyclohexanone | 3 | 372378 | 97 | 105,89 |
| **8.955** | Benzene, 1,2,3-trimethyl- | 1,2,3-trimethylbenzene | 3 | 65744 | 89 | 21,47 |
| **9.836** | RT9.836 | - | 3 | 24252 | - | 1,97 |
| **19.356** | RT19.356 | - | 3 | 13661 | - | |
| **19.876** | RT19.876 | - | 3 | 26620 | - | 19,77 |
| **20.251** | Phenol, 3,5-bis(1,1-dimethylethyl)- | 3,5-di-tert-butylphenol | 3 | 66310 | 15,07 | 19.750 |
| **21.863** | Pentanoic acid, 2,2,4-trimethyl-3-carboxyisopropyl, isobutyl | | 3 | 43678 | 30,83 | 21.365 |
| **22.031** | RT22.031 | - | 3 | 11421 | - | 19,12 |
| **23.186** | RT23.186 | - | 3 | 43244 | - | 24,65 |
| **23.928** | RT23.928 | - | 3 | 21621 | - | 2,09 |
| **25.084** | RT25.084 | - | 3 | 23973 | - | 9,16 |
| **25.237** | RT25.237 | - | 3 | 12236 | - | 11,26 |
| **26.058** | RT26.058 | - | 3 | 43790 | - | 25,86 |
| **26.555** | RT26.555 | - | 3 | 16801 | - | 9,42 |
| **26.860** | 1,2-Benzenedicarboxylic acid, bis (2-methylpropyl | | 3 | 64518 | | |
| **27.183** | RT27.183 | - | 3 | 13652 | - | 3,27 |
| **27.659** | RT27.659 | - | 3 | 14328 | - | 2,61 |
| **28.627** | RT28.627 | - | 3 | 35917 | - | 20,12 |
| **31.002** | RT31.002 | - | 3 | 31237 | - | 13,15 |
| **31.992** | Heptadecane, 2,6,10,15-tetramethyl- | 2,6,10,15-tetramethylheptadecane | 3 | 49939 | 84 | 8,48 |
| **33.405** | Heneicosane | heneicosane | 3 | 165144 | | 29,43 |
| **34.769** | RT34.769 | - | 3 | 461254 | - | 115,7 |
| **36.083** | RT36.083 | - | 3 | 1096820 | - | 189,73 |
| **36.646** | Triphenylphosphine oxide | | 3 | 1060974 | | 48,95 |

| *Xylaria striata* HS - SPME VCQ14 | | | | | | |
|---|---|---|---|---|---|---|
| **RT** | **Name** | **Common name** | **Identification level** | **Area** | **SI** | **S/N** |
| **37.353** | RT37.353 | - | 3 | 2311765 | - | 301,41 |
| **38.133** | 2-methyloctacosane | 2-methyloctacosane | 3 | 70513 | | 0,61 |
| **39.340** | RT39.340 | - | 3 | 113456 | - | 3,2 |
| **39.466** | RT39.466 | - | 3 | 56394 | - | 6,39 |
| **39.667** | 4,4'-Ethylenebis(2,6-di-tert-butylphenol) | | 3 | 236344 | | 380,75 |
| **39.777** | RT39.777 | - | 3 | 3981528 | - | 785,57 |
| **40.544** | RT40.544 | - | 3 | 155563 | - | 9,34 |
| **40.757** | RT40.757 | - | 3 | 4190190 | - | 783,21 |

**Table S13.** Volatile organic compounds identified in *Lentinus* sp. using HS-SPME-GC-MS under laboratory conditions.

| *Lentinus* sp. HS - SPME VCQ15 | | | | | | |
|---|---|---|---|---|---|---|
| **RT** | **Name** | **Common name** | **Identification level** | **Area** | **SI** | **S/N** |
| **3.162** | Cyclohexane, methyl- | methylcyclohexane | 3 | 235289 | 98 | 73,37 |
| **3.681** | Toluene | toluene | 3 | 768823 | 99 | 1264,21 |
| **4.510** | Heptane, 2,4-dimethyl- | 2,4-dimethylheptane | 3 | 95044 | 89 | 89,91 |
| **4.871** | 2,4-Dimethylhept-1-ene | 2,4-dimethyl-1-heptene | 3 | 172492 | 99 | 91,05 |
| **5.290** | Hexane, 2,3,4-trimethyl- | | 3 | 152479 | | |
| **5.433** | Benzene, 1,3-dimethyl- | m-xylene | 3 | 309745 | 100 | 9,3 |
| **5.929** | Cyclohexanone | cyclohexanone | 3 | 2909772 | 99 | 373,69 |
| **6.036** | RT6.036 | - | 3 | 84630 | - | 4,27 |
| **6.609** | Benzene, (1-methylethyl)- | cumene | 3 | 45586 | 92 | 69,18 |
| **6.838** | (1R)-2,6,6-Trimethylbicyclo [3.1.1] hept-2-ene | alpha-pinene | 3 | 1017329 | 100 | 60,32 |
| **7.467** | RT7.467 | - | 3 | 325171 | - | 32,27 |
| **7.837** | . beta. -Pinene | beta-pinene | 3 | 1512954 | 99 | 0,12 |
| **8.052** | 3-Heptanone, 5-methyl- | 5-methylheptan-3-one | 3 | 520772 | 90 | 2,21 |
| **8.319** | . beta. -Myrcene | beta-myrcene | 3 | 41255310 | 99 | 245,63 |
| **8.463** | 2-Octen-1-ol, (E)- | trans-2-octen-1-ol | 3 | 1090615 | 88 | 1,44 |
| **8.625** | Octane, 3,3-dimethyl- | 3,3-dimethyloctane | 3 | 982663 | 99 | 8,46 |
| **8.718** | Decane, 4-methyl- | 4-methyldecane | 3 | 1014520 | 98 | 15,53 |
| **8.937** | RT8.937 | | 3 | 1749444 | | |
| **9.138** | D-Limonene | d-limonene | 3 | 7660197 | 100 | 1347,61 |
| **9.458** | Cyclopropane, 1,2-dimethyl-3-pentyl- | 1,2-dimethyl-3-pentylcyclopropane | 3 | 609873 | 88 | 2,24 |
| **9.787** | Undecane, 5,7-dimethyl- | 5,7-dimethylundecane | 3 | 1017595 | 98 | 33,66 |
| **10.123** | 1-Octanol | 1-octanol | 3 | 1304774 | 100 | 270,77 |
| **10.317** | 2-Undecene, 4,5-dimethyl-, [R*, S*-(Z)]- | (2z)-4,5-dimethyl-2-undecene | 3 | 543416 | 100 | 13,87 |
| **10.423** | 1-Dodecanol, 3,7,11-trimethyl- | 3,7,11-trimethyl-1-dodecanol | 3 | 469340 | 100 | 6,13 |
| **10.559** | Cyclohexene, 3-methyl-6-(1-methylethylidene)- | isoterpinolene | 3 | 340896 | 96 | 11,97 |
| **10.679** | Nonane, 5-butyl- | | 3 | 222262 | | |
| **10.887** | Decane, 2,3,5,8-tetramethyl- | | 3 | 772702 | | |
| **11.003** | Decane, 3,7-dimethyl- | 3,7-dimethyldecane | 3 | 157353 | 99 | 4,09 |
| **11.201** | 1-Undecene, 4-methyl- | 4-methyl-1-undecene | 3 | 143359 | 72 | 2,52 |

| *Lentinus* sp. HS - SPME VCQ15 | | | | | | |
|---|---|---|---|---|---|---|
| **RT** | **Name** | **Common name** | **Identification level** | **Area** | **SI** | **S/N** |
| **12.365** | RT12.365 | - | 3 | 59337 | - | 1,96 |
| **13.221** | Dodecane | n-dodecane | 3 | 72151 | 96 | 16,56 |
| **13.361** | Decanal | decanal | 3 | 70382 | 96 | 5,29 |
| **13.562** | Undecane, 4,6-dimethyl- | 4,6-dimethylundecane | 3 | 65080 | 94 | 9,66 |
| **14.418** | Nonane, 4,5-dimethyl- | 4,5-dimethylnonane | 3 | 51077 | 77 | 1,65 |
| **15.106** | Dodecane, 4,6-dimethyl- | 4,6-dimethyldodecane | 3 | 58472 | 96 | 3,87 |
| **15.712** | 11-Methyldodecanol | isotridecanol | 3 | 61299 | 96 | 11,69 |
| **15.899** | 1-Undecene, 7-methyl- | 7-methyl-1-undecene | 3 | 82713 | 97 | 15,52 |
| **16.113** | RT16.113 | - | 3 | 83864 | - | |
| **16.181** | RT16.181 | - | 3 | 82178 | - | 72,28 |
| **17.459** | RT17.459 | - | 3 | 112736 | - | |
| **18.326** | Caryophyllene | beta-caryophyllene | 3 | 241859 | 99 | 3,42 |
| **18.855** | (3aR,4R,8R,8aS)-3a,4,8a-Trimethyl-7-methylenedecahydro | (. +/-.)-gymnomitrene | 3 | 65935 | 95 | 2,17 |
| **19.047** | Humulene | humulene | 3 | 76816 | 95 | 8,81 |
| **19.252** | 2,5-Cyclohexadiene-1,4-dione, 2,6-bis(1,1-dimethylethyl)- | 2,6-di-tert-butyl-p-benzoquinone | 3 | 60245 | 96 | 17,7 |
| **19.338** | 2,5-cyclohexadien-1-one, 2,6-bis(1,1-dimethylethyl)-4-hydroxy | 2,6-di(tert-butyl)-4-hydroxy-4-methyl-2,5-cyclohexadien-1-one | 3 | 56590 | 96 | 5,02 |
| **19.499** | RT.12.499 | - | 3 | 111249 | - | 44,28 |
| **20.189** | Butylated Hydroxytoluene | butylhydroxytoluene | 3 | 1228432 | 100 | 914,61 |
| **20.584** | (3aS,8aS)-6,8a-Dimethyl-3-(propan-2-ylidene)-1,2,3,3a,4,5,8,8a | dauca-4(11),8-diene | 3 | 42109 | 90 | 28,81 |
| **21.855** | Pentanoic acid, 2,2,4-trimethyl-3-carboxyisopropyl, isobutyl este | 1-isobutyl 4-isopropyl 3-isopropyl-2,2-dimethylsuccinate | 3 | 41553 | 96 | 67,42 |

**Table S14.** Volatile organic compounds identified in *Lentinus* sp. using SHS-SPME-GC-MS in situ.

| *Lentinus* sp. SHS - SPME VCQ15 | | | | | | |
|---|---|---|---|---|---|---|
| **RT** | **Name** | **Common name** | **Identification level** | **Area** | **SI** | **S/N** |
| **3.976** | Hexanal | Hexanal | 3 | 91277 | 95 | 72,18 |
| **4.387** | RT4.387 | - | 3 | 20631 | - | 30,27 |
| **4.580** | 2-Pentenal, 2-methyl- | 2-methyl-2-pentenal | 3 | 94572 | 95 | 54,93 |
| **4.816** | 2-Hexanol, 2-methyl- | 2-methyl-2-hexanol | 3 | 34826 | 89 | 35,52 |
| **5.347** | RT5.347 | - | 3 | 72854 | - | 11,82 |
| **5.646** | RT5.646 | - | 3 | 940971 | - | 918,06 |
| **5.847** | 2-Hexenal, 2-ethyl- | 2-ethyl-2-hexenal | 3 | 200755 | 95 | 19,03 |
| **6.764** | . alpha. -Pinene | alpha-pinene | 3 | 51938 | 97 | 42,91 |
| **7.132** | 2-Heptenal, 2-methyl- | (2e)-2-methyl-2-heptenal | 3 | 23901 | 92 | 15,84 |
| **7.442** | RT7.442 | - | 3 | 41258 | - | 21,29 |
| **7.802** | 1-Octen-3-one | 1-octen-3-one | 3 | 1615716 | 99 | 2,71 |
| **7.985** | 1-Octen-3-ol | 1-Octen-3-ol | 3 | 9942663 | 96 | 151,01 |
| **8.152** | RT8.152 | - | 3 | 29564 | - | 38,25 |
| **8.318** | 3-Octanol | 3-Octanol | 3 | 4904212 | 99 | 2828,26 |
| **8.423** | Octanal | Octanal | 3 | 106268 | 99 | 3,45 |
| **8.916** | RT8.916 | - | 3 | 41403 | - | 18,98 |
| **9.073** | D-Limonene | D-Limonene | 3 | 2288856 | 100 | 1699,71 |
| **9.166** | RT9.166 | - | 3 | 34211 | - | |
| **9.515** | RT9.515 | - | 3 | 50733 | - | |
| **9.763** | 2-Octenal, (E)- | trans-2-Octenal | 3 | 337256 | 99 | 224,15 |
| **10.096** | RT10.096 | - | 3 | 80495 | - | |
| **10.492** | RT10.492 | - | 3 | 50058 | - | 30,6 |
| **10.824** | Linalool | Linalool | 3 | 28961 | 92 | 12,44 |
| **10.919** | RT10.919 | - | 3 | 52191 | - | |
| **11.394** | Octanoic acid, methyl ester | Methyl octanoate | 3 | 64719 | 93 | 41,13 |
| **11.944** | RT11.944 | - | 3 | 12931 | - | |
| **12.165** | RT12.15 | - | 3 | 44738 | - | 48,53 |
| **16.203** | RT16.203 | - | 3 | 12706 | - | 9,7 |
| **17.484** | Hexane, 4-ethyl-2-methyl- | 4-Ethyl-2-methylhexane | 3 | 17849 | 85 | 9,23 |
| **18.317** | RT18.317 | - | 3 | 13719 | - | 3,47 |
| **18.839** | RT18.839 | - | 3 | 10768 | - | 2,74 |
| **20.176** | Butylated Hydroxytoluene | Butylhydroxytoluene | 3 | 275355 | 99 | 227,91 |

**Table S15.** Volatile organic compounds identified in *Auricularia nigricans* using HS-SPME-GC-MS under laboratory conditions.

| *Auricularia nigricans* HS-SPME VCQ16 | | | | | | |
|---|---|---|---|---|---|---|
| **RT** | **Name** | **Common name** | **Identification level** | **Area** | **SI** | **S/N** |
| **3.135** | Oxime-, methoxy-phenyl-_ | methyl N-hydroxybenzenecarboximidate | 3 | 2185581 | 87 | 608.84 |
| **5.545** | Acetic acid, (1,2,3,4,5,6,7,8-octahydro-3,8,8-trimethylnaphth-2-yl) methyl ester | (3,8,8-Trimethyl-1,2,3,4,5,6,7,8-octahydro-2-naphthalenyl) methyl acetate | 3 | 2185581 | 99 | 709.66 |
| **5.790** | (E)-. beta. -Famesene | (E)-beta-farnesene | 3 | 266789 | 95 | 33.54 |
| **7.760** | RT7.760 | RT7.760 | 3 | 70388 | 82 | 2.96 |

**Table S16.** Volatile organic compounds identified in *Laetiporus* sp. using HS-SPME-GC-MS under laboratory conditions.

| *Laetiporus* sp. HS - SPME VCQ17 | | | | | | |
|---|---|---|---|---|---|---|
| **RT** | **Name** | **Common name** | **Identification level** | **Area** | **SI** | **S/N** |
| **3.039** | Pentane, 2,2,3,4-tetramethyl- | 2,2,3,4-tetramethylpentane | 3 | 112049 | 94 | 30,27 |
| **3.114** | Cyclohexane, methyl- | methylcyclohexane | 3 | 2141517 | 99 | 1233,03 |
| **4.378** | Acetic acid, butyl ester | butyl acetate | 3 | 66854 | 85 | 12,3 |
| **5.275** | Ethylbenzene | ethylbenzene | 3 | 184154 | 95 | 195,08 |
| **5.423** | Benzene, 1,3-dimethyl- | m-xylene | 3 | 856737 | 99 | 4,31 |
| **5.897** | Cyclohexanone | cyclohexanone | 3 | 1139267 | 95 | 405,67 |
| **8.928** | Benzene, 1,2,3-trimethyl- | 1,2,3-trimethylbenzene | 3 | 939284 | 99 | 279,6 |
| **9.090** | D-Limonene | D-Limonene | 3 | 179159 | 90 | 29,33 |
| **9.272** | Indane | Indane | 3 | 324790 | 98 | 39,26 |
| **9.637** | 2-Tolyloxirane | 2-Tolyloxirane | 3 | 525810 | 99 | 47 |
| **9.798** | Benzene, 1-ethyl-3,5-dimethyl- | 5-Ethyl-m-xylene | 3 | 1059105 | 94 | 12,06 |
| **10.031** | Benzene, 1-methyl-4-propyl- | 1-methyl-4-propylbenzene | 3 | 269137 | 96 | 4,26 |
| **10.294** | RT10.294 | - | 3 | 744101 | - | 1,29 |
| **10.480** | Benzene, 4-ethyl-1,2-dimethyl- | 4-ethyl-o-xylene | 3 | 915406 | 99 | 12,27 |
| **11.010** | RT11.010 | - | 3 | 128864 | - | 1,87 |
| **11.236** | RT11.236 | - | 3 | 248515 | - | 52,2 |
| **11.337** | Benzene, 1,2,4,5-tetramethyl- | 1,2,4,5-tetramethylbenzene | 3 | 614436 | 99 | 136,29 |
| **12.159** | RT12.159 | - | 3 | 348886 | - | 152,84 |
| **17.429** | 3-Isopropyl-6,8a-dimethyl-1,2,4,5,8,8a-hexa | Daucene | 3 | 122413 | 97 | 38,69 |
| **17.683** | Cyclohexane, 1-ethenyl-1-methyl-2,4-bis(1-methylethenyl)-, [1S-(1. alpha.,2. beta.,4. beta.)]- | (-)-cis-. beta. -Elemene | 3 | 426583 | 99 | 39,54 |
| **18.462** | (3S,3aS,8aR)-6,8a-Dimethyl-3-(prop-1-en-2-yl)-1,2,3,3 | Isodaucene | 3 | 124954 | 96 | 16,69 |
| **19.030** | (1S,7S,8aR)-1,8a-Dimethyl-7-(prop-1-en-2-yl)-1,2,3,7,8,8 | Eremophila-1(10),8,11-triene | 3 | 73783 | 93 | 13,59 |
| **19.116** | RT19.116 | - | 3 | 124909 | - | 29,83 |
| **19.326** | RT19.326 | - | 3 | 183275 | - | 7,08 |
| **19.472** | 4a,8-Dimethyl-2-(prop-1-en-2-yl)-1,2,3,4,4a,5,6,7-octa | 2-Isopropenyl-4a,8-dimethyl-1,2,3,4,4a,5,6,7-octahydronaphthalene | 3 | 708498 | 100 | 14,37 |
| **19.593** | RT19.593 | - | 3 | 140811 | - | 11,34 |

| | | | | | | |
|---|---|---|---|---|---|---|
| **19.730** | Naphthalene, decahydro-4a-methyl-1-methylene-7- ( | beta-selinene | 3 | 231022 | 98 | 2,13 |
| **19.984** | cis-. alpha. -Bisabolene | (Z)-alpha-Bisabolene | 3 | 1627865 | 97 | 14,84 |
| **20.172** | Butylated Hydroxytoluene | Butylhydroxytoluene | 3 | 2317267 | 99 | 610,08 |
| **20.574** | (3aS,8aS)-6,8a-Dimethyl-3-(propan-2-ylidene)-1,2,3,3a,4,5,8,8 | Dauca-4(11),8-diene | 3 | 3378156 | 100 | 1663,17 |
| **20.863** | Cyclohexene, 4-[(1E)-1,5-dimethyl-1,4-hexadien-1-yl]-1- | (E)-alpha-bisabolene | 3 | 221837 | 66 | 4,96 |
| **21.864** | Pentanoic acid, 2,2,4-trimethyl-3-carboxyisopropyl | 1-Isobutyl 4-isopropyl 3-isopropyl-2,2-dimethylsuccinate | 3 | 229842 | 97 | 91,82 |
| **21.981** | RT21.981 | - | 3 | 220805 | - | 23,83 |
| **23.842** | RT23.842 | - | 3 | 122474 | - | |
| **33.427** | Heneicosane | Heneicosane | 3 | 278603 | 98 | 34,97 |
| **34.785** | RT34.785 | - | 3 | 653061 | - | 90,39 |
| **36.098** | RT36.098 | - | 3 | 1306670 | - | 117,66 |
| **36.894** | 2-methyloctacosane | 2-methyloctacosane | 3 | 103827 | 85 | 0,19 |
| **37.369** | RT37.369 | - | 3 | 1939462 | - | 290,43 |
| **38.140** | Octacosane | n-Octacosane | 3 | 50035 | 94 | 0,45 |
| **38.599** | RT38.599 | - | 3 | 2727376 | - | 250,26 |
| **39.351** | RT39.351 | - | 3 | 113492 | - | 6,27 |
| **39.466** | RT39.466 | - | 3 | 48216 | - | 4,14 |
| **39.791** | RT39.791 | - | 3 | 3070690 | - | 331,97 |
| **40.768** | RT40.768 | - | 3 | 3085912 | - | 771,57 |

**Table S17.** Volatile organic compounds identified in *Agarical* using HS-SPME-GC-MS under laboratory conditions.

| Agarical HS - SPME VCQ18 | | | | | | |
|---|---|---|---|---|---|---|
| **RT** | **Name** | **Common name** | **Identification level** | **Area** | **SI** | **S/N** |
| **3.359** | RT3.359 | - | 3 | 4623648 | - | 224,29 |
| **4.616** | 2-Pentenal, 2-methyl- | 2-methyl-2-pentenal | 3 | 2830794 | 93 | 29,63 |
| **4.780** | 2-Pentanone, 4-hydroxy-4-methyl- | Diacetone alcohol | 3 | 6815381 | 98 | 2554,41 |
| **5.671** | RT5.671 | - | 3 | 23042597 | - | 987,72 |
| **7.877** | 1-Octen-3-ol | 1-Octen-3-ol | 3 | 2121192 | 99 | 300,7 |
| **8.017** | 3-Octanone | 3-Octanone | 3 | 2656319 | 97 | 198,65 |
| **8.285** | RT8.285 | - | 3 | 4173828 | - | |
| **8.924** | Benzene, 1-ethyl-2-methyl- | 1-Ethyl-2-methylbenzene | 3 | 417311 | 87 | 28,57 |
| **9.062** | D-Limonene | D-Limonene | 3 | 29296795 | 100 | 5223,43 |
| **9.558** | 1,3,6-Octatriene, 3,7-dimethyl-, (Z)- | (z)-beta-ocimene | 3 | 261483 | 93 | 0,03 |
| **9.790** | 1,3,5-Cycloheptatriene, 3,7,7-trimethyl- | 3,7,7-trimethylcyclohepta-1,3,5-triene | 3 | 135573 | 83 | 1,09 |
| **9.900** | Pentanal, 2,4-dimethyl- | 2,4-dimethylpentanal | 3 | 182274 | 93 | 62,4 |
| **10.165** | 2-Octanol, 2-methyl-6-methylene- | 2-Methyl-6-methyleneoctan-2-ol | 3 | 157229 | 89 | 58,95 |
| **10.306** | Benzene, 1-ethyl-2,3-dimethyl- | 1-Ethyl-2,3-dimethylbenzene | 3 | 292830 | 95 | 3,82 |
| **10.816** | Linalool | Linalool | 3 | 1279141 | 96 | 103,05 |
| **10.910** | Nonanal | nonanal | 3 | 3208698 | 98 | 20,82 |
| **11.197** | Fenchol, exo- | Fenchol | 3 | 1067437 | 91 | 44,79 |
| **11.338** | 1,3-Cyclopentadiene, 1,2,3,4-tetramethyl- | 1,2,3,4-tetramethylcyclopenta-1,3-diene | 3 | 559943 | 97 | 109,05 |
| **11.933** | (+)-2-Bornanone | D-camphor | 3 | 545264 | 98 | 51,66 |
| **12.396** | RT12.396 | - | 3 | 441363 | - | 17,84 |
| **12.488** | Octane, 2,7-dimethyl- | 2,7-dimethyloctane | 3 | 266808 | 95 | 10,8 |
| **12.646** | Cyclohexanol, 5-methyl-2-(1-methylethyl)-, [1S-(1. alpha.,2. | menthol | 3 | 110774 | 95 | 2,07 |
| **13.223** | Tridecane | Tridecane | 3 | 434721 | 99 | 42,12 |
| **13.540** | RT13.540 | - | 3 | 86802 | - | 8,41 |

| Agarical HS - SPME VCQ18 | | | | | | |
|---|---|---|---|---|---|---|
| **RT** | **Name** | **Common name** | **Identification level** | **Area** | **SI** | **S/N** |
| **14.520** | RT14.520 | - | 3 | 189340 | - | |
| **14.706** | Dodecane, 2-methyl- | 2-methyldodecane | 3 | 79017 | 87 | 1,73 |
| **15.096** | Nonane, 5-(2-methylpropyl)- | 5-Isobutylnonane | 3 | 51720 | 94 | 1,91 |
| **15.302** | RT15.302 | - | 3 | 137088 | - | 1,29 |
| **15.447** | Cyclohexanol, 2-(1,1-dimethylethyl)- | 2-tert-Butylcyclohexanol | 3 | 98846 | 96 | 2,66 |
| **15.565** | RT15.565 | - | 3 | 88133 | - | 4,43 |
| **15.721** | RT15.721 | - | 3 | 329478 | - | 175,35 |
| **16.347** | Hexadecane | Hexadecane | 3 | 70028 | 94 | 3,08 |
| **17.337** | . alfa. -Copaene | alpha-copaene | 3 | 51039 | 99 | 14,32 |
| **17.478** | Hexane, 4-ethyl-2-methyl- | 4-Ethyl-2-methylhexane | 3 | 601894 | 99 | 45,19 |
| **18.310** | Caryophyllene | beta-caryophyllene | 3 | 2333242 | 100 | 81,83 |
| **18.585** | Bicyclo[3.1.1]hept-2-ene, 2,6-dimethyl-6-(4-methyl-3-pentenyl)- | alpha-Bergamotene | 3 | 72924 | 96 | 5,64 |
| **18.835** | (3aR,4R,8R,8aS)-3a,4,8a-Trimethyl-7-methylenedecahydro-4,8- | (. +/-.)-Gymnomitrene | 3 | 80382 | 90 | 0,16 |
| **19.034** | Humulene | Humulene | 3 | 293071 | 99 | 25,72 |
| **19.247** | 2,5-Cyclohexadiene-1,4-dione, 2,6-bis(1,1-dimethylethyl)- | 2,6-Di-tert-butyl-P-benzoquinone | 3 | 271053 | 95 | 64,49 |
| **20.168** | Butylated Hydroxytoluene | Butylhydroxytoluene | 3 | 15882529 | 100 | 438,21 |
| **20.699** | 1H-Cycloprop[e]azulene, decahydro-1,1,7-trimethyl-4-methylene | Spathulenol | 3 | 70176 | 95 | 0,18 |
| **20.841** | Selina-3,7(11)-diene | Selina-3,7(11)-diene | 3 | 44675 | 97 | 10,81 |

**Table S18.** Volatile organic compounds identified in *Crepidotus* sp. using HS-SPME-GC-MS under laboratory conditions.

| *Crepidotus* sp. HS - SPME VCQ19 | | | | | | |
|---|---|---|---|---|---|---|
| **RT** | **Name** | **Common name** | **Identification level** | **Area** | **SI** | **S/N** |
| **4.621** | 2-Pentenal, 2-methyl- | 2-methyl-2-pentenal | 3 | 6370773 | 100 | 1041,5 |
| **4.789** | 2-Pentanone, 4-hydroxy-4-methyl- | Diacetone alcohol | 3 | 5879199 | 99 | 928,95 |
| **5.680** | RT5.680 | - | 3 | 28171147 | - | 5731,47 |
| **7.868** | 1-Octen-3-ol | 1-Octen-3-ol | 3 | 5044022 | 99 | 579,5 |
| **8.013** | 3-Octanone | 3-Octanone | 3 | 5159685 | 97 | 209,25 |
| **9.066** | D-Limonene | D-Limonene | 3 | 20424793 | 100 | 2198,56 |
| **9.574** | RT9.574 | - | 3 | 327493 | - | 1,64 |
| **9.898** | Heptanal, 2-methyl- | 2-Methylheptanal | 3 | 196744 | 96 | 97,09 |
| **10.144** | 1-Octanol | 1-octanol | 3 | 905449 | 98 | 59,73 |
| **10.302** | RT10.302 | - | 3 | 366379 | - | |
| **10.541** | . alpha. -Terpineol | alpha-terpineol | 3 | 728066 | 94 | 59,12 |
| **10.815** | Linalool | Linalool | 3 | 2142018 | 97 | 168,43 |
| **10.906** | Nonanal | nonanal | 3 | 3567213 | 99 | 44,2 |
| **11.196** | Fenchol, exo- | Fenchol | 3 | 1059571 | 93 | 39,22 |
| **11.935** | (+)-2-Bornanone | D-camphor | 3 | 498989 | 98 | 58,24 |
| **12.399** | RT12.399 | - | 3 | 339561 | - | 13,36 |
| **12.487** | 2,2-Dimethyleicosane | 2,2-Dimethyleicosane | 3 | 251886 | 98 | 10,01 |
| **12.648** | Cyclohexanol, 1-methyl-4-(1-methylethyl)- | p-Menthan-1-ol | 3 | 165624 | 86 | 3,05 |
| **13.057** | RT13.057 | - | 3 | 369276 | - | |
| **13.220** | Tridecane | Tridecane | 3 | 640344 | 99 | 39,83 |
| **13.899** | RT13.899 | - | 3 | 134573 | - | 31,19 |
| **14.531** | RT14.531 | - | 3 | 177692 | - | |
| **15.451** | Cyclohexanol, 2-(1,1-dimethylethyl)- | 2-tert-Butylcyclohexanol | 3 | 83462 | 99 | 2,87 |
| **15.565** | RT15.565 | - | 3 | 128679 | - | 6,45 |
| **15.721** | RT15.721 | - | 3 | 241269 | - | 134,77 |
| **17.039** | RT17.039 | - | 3 | 4757582 | - | 769,07 |
| **17.477** | Hexane, 4-ethyl-2-methyl- | 4-Ethyl-2-methylhexane | 3 | 396425 | 99 | 23,37 |
| **17.599** | 3-Tetradecene, (E)- | 3-Tetradecene | 3 | 408957 | 99 | 5,03 |
| **17.757** | Pentadecane | Pentadecane | 3 | 606020 | 99 | 22,56 |

| *Crepidotus* sp. HS - SPME VCQ19 | | | | | | |
|---|---|---|---|---|---|---|
| **RT** | **Name** | **Common name** | **Identification level** | **Area** | **SI** | **S/N** |
| **18.019** | Bicyclo[7.2.0]undec-4-ene, 4,11,11-trimethyl-8-methylene-,[1R-( | beta-caryophyllene | 3 | 205810 | 95 | 12,23 |
| **18.137** | (3aR,4R,8R,8aS)-3a,4,7,8a-Tetramethyl-1,2,3,3a,4,5,8,8a-octa | (+)-alpha-barbatene | 3 | 338911 | 99 | 15,96 |
| **18.311** | Caryophyllene | beta-caryophyllene | 3 | 2971172 | 100 | 35,48 |
| **18.837** | (3aR,4R,8R,8aS)-3a,4,8a-Trimethyl-7-methylenedecahydro-4,8 | (.+/-.)-Gymnomitrene | 3 | 755880 | 99 | 1,57 |
| **19.036** | Humulene | Humulene | 3 | 590158 | 99 | 38,74 |
| **19.246** | 2,5-Cyclohexadiene-1,4-dione, 2,6-bis(1,1-dimethylethyl)- | 2,6-Di-tert-butyl-P-benzoquinone | 3 | 321082 | 95 | 38,84 |
| **19.491** | 1s,4R,7R,11R-1,3,4,7-Tetramethyltricyclo [5.3.1.0(4,11)] undec | 1s,4R,7R,11R-1,3,4,7-Tetramethyltricyclo [5.3.1.0(4,11)] undec-2-en-8-one | 3 | 169369 | 98 | 22,57 |
| **19.720** | Bicyclo[5.3.0]decane, 2-methylene-5-(1-methylvinyl)-8-methyl- | 7-Isopropenyl-1-methyl-4-methylenedecahydroazulene | 3 | 187337 | 93 | 1,09 |
| **20.164** | Phenol, 3,5-bis(1,1-dimethylethyl)- | 3,5-Di-tert-butylphenol | 3 | 21046916 | 97 | 4191,96 |
| **20.694** | 1H-Cycloprop[e]azulene, decahydro-1,1,7-trimethyl-4-methylene- | Spathulenol | 3 | 154305 | 94 | 0,55 |
| **20.841** | Selina-3,7(11)-diene | Selina-3,7(11)-diene | 3 | 191661 | 97 | 31,41 |
| **21.730** | 3-Octadecene, (E)- | (E)-3-Octadecene | 3 | 240291 | 94 | 6,38 |
| **21.863** | Hexadecane | Hexadecane | 3 | 531298 | 97 | 21,39 |
| **22.604** | 2,6-Bis(1,1-dimethylethyl)-4-(1-oxopropyl) phenol | 2,6-Bis(1,1-dimethylethyl)-4-(1-oxopropyl) phenol | 3 | 105731 | 98 | 97,75 |
| **23.201** | RT23.201 | - | 3 | 178249 | - | 11,82 |
| **23.843** | 2-methyloctacosane | 2-methyloctacosane | 3 | 77408 | 95 | 10,05 |

**Table S19.** Volatile organic compounds identified in *Cookeina* sp. using DHS-SPME-GC-MS in situ.

| *Cookeina* **sp. DHS-SPME VCQ23** | | | | | | |
|---|---|---|---|---|---|---|
| **RT** | **Name** | **Common name** | **Identification level** | **Area** | **SI** | **S/N** |
| **3.077** | Cyclohexane, methyl- | methylcyclohexane | 3 | 1576048 | 98 | 794,69 |
| **5.261** | Hexane, 2,3,4-trimethyl- | 2,3,4-trimethylhexane | 3 | 127202 | 94 | 33,07 |
| **5.401** | Benzene, 1,3-dimethyl- | m-xylene | 3 | 107013 | 93 | 4,7 |
| **6.807** | . alpha. -Pinene | alpha-pinene | 3 | 16932 | 83 | 21,06 |
| **7.451** | RT7.451 | - | 3 | 29111 | - | 36,24 |
| **9.772** | Undecane, 4,7-dimethyl- | 4,7-Dimethylundecane | 3 | 113854 | 97 | 17,09 |
| **9.908** | Octane, 2,3,3-trimethyl- | 2,3,3-Trimethyloctane | 3 | 41028 | 90 | 4,89 |
| **10.812** | Undecane | Undecane | 3 | 336495 | 96 | 26,06 |
| **12.416** | 1-Nonene, 4,6,8-trimethyl- | 4,6,8-trimethyl-1-nonene | 3 | 37645 | 87 | 2,31 |
| **12.534** | Undecane, 3-methyl- | 3-methylundecane | 3 | 60005 | 90 | 8,24 |
| **13.238** | Tridecane | Tridecane | 3 | 798287 | 99 | 83,05 |
| **13.555** | Dodecane, 2,6,10-trimethyl- | Farnesane | 3 | 157915 | 93 | 4,26 |
| **14.605** | 1-Dodecanol, 3,7,11-trimethyl- | 3,7,11-Trimethyl-1-dodecanol | 3 | 44013 | 85 | 2,47 |
| **15.691** | 1-Hexanol, 5-methyl-2-(1-methylethyl)- | 2-Isopropyl-5-methylhexan-1-ol | 3 | 185077 | 92 | 7,42 |
| **16.368** | Octane, 5-ethyl-2-methyl- | 5-Ethyl-2-methyloctane | 3 | 79099 | 88 | 2,2 |
| **16.615** | Decane, 3-ethyl-3-methyl- | 3-Ethyl-3-methyldecane | 3 | 96314 | 93 | 5,14 |
| **17.141** | Tridecane, 3-methyl- | 3-Methyltridecane | 3 | 205202 | 88 | 4,5 |
| **20.200** | Butylated Hydroxytoluene | 2,6-Di-tert-butyl-4-methylphenol | 3 | 793143 | 99 | 439,76 |
| **36.198** | Heneicosane | Heneicosane | 3 | 176480 | 94 | 24,43 |

**Table S20.** Volatile organic compounds identified in *Cookeina* sp. using HS-SPME-GC-MS under laboratory conditions.

| *Cookeina* **sp. HS-SPME VCQ23** | | | | | | |
|---|---|---|---|---|---|---|
| **RT** | **Name** | **Common name** | **Identification level** | **Area** | **SI** | **S/N** |
| **1.843** | n-Hexane | Hexane | 3 | 175201 | 97 | 122,87 |
| **3.441** | Hexane, 3,3,4-trimethyl- | 3,3,4-trimethylhexane | 3 | 112424 | 95 | 9,44 |
| **3.535** | Heptane, 3-methyl- | 3-methylheptane | 3 | 132264 | 84 | 24,57 |
| **3.632** | Cyclohexane, 1,3-dimethyl-, cis- | cis-1,3-Dimethylcyclohexane | 3 | 130828 | 97 | 77,68 |
| **3.966** | Octane | Octane | 3 | 174291 | 98 | 18,46 |
| **4.077** | Cyclohexane, 1,4-dimethyl-, cis- | 1,4-dimethylcyclohexane | 3 | 42169 | 93 | 4,48 |
| **4.595** | Cyclohexane, ethyl- | ethylcyclohexane | 3 | 425140 | 93 | 25,08 |
| **4.760** | 2-Hexanol, 2-methyl- | 2-methyl-2-hexanol | 3 | 862543 | 89 | 387,81 |
| **5.334** | Benzene, 1,3-dimethyl- | m-xylene | 3 | 742810 | 98 | 2,58 |
| **5.519** | 5-Oxa-6-azaspiro [3.4] oct-6-ene | 5-Oxa-6-azaspiro [3.4] oct-6-ene | 3 | 214151 | 94 | 82,93 |
| **5.699** | 1-Ethyl-4-methylcyclohexane | 1-Ethyl-4-methylcyclohexane | 3 | 129829 | 90 | 2,74 |
| **5.839** | o-Xylene | o-Xylene | 3 | 299409 | 98 | 0,81 |
| **5.958** | Nonane | Nonane | 3 | 226732 | 97 | 32,24 |
| **6.196** | Oxime-, methoxy-phenyl-_ | methyl N-hydroxybenzenecarboximidate | 3 | 2271842 | 99 | 798,18 |
| **7.345** | RT7.345 | - | 3 | 349481 | - | 328,44 |
| **8.548** | Octane, 3,3-dimethyl- | 3,3-Dimethyloctane | 3 | 219305 | 98 | 25,28 |
| **8.649** | Nonane, 2,6-dimethyl- | 2,6-dimethylnonane | 3 | 244137 | 98 | 23,61 |
| **8.877** | Decane, 4-methyl- | 4-methyldecane | 3 | 276747 | 92 | 10,8 |
| **9.750** | Undecane, 4,7-dimethyl- | 4,7-Dimethylundecane | 3 | 440207 | 99 | 13,84 |
| **10.291** | 1-Nonanol, 4,8-dimethyl- | 4,8-Dimethyl-1-nonanol | 3 | 260206 | 99 | 36,69 |
| **10.400** | RT10.400 | - | 3 | 1410548 | - | 957,1 |
| **10.517** | Heptane, 1,1'-oxybis- | Ether, diheptyl | 3 | 246865 | 94 | 2,24 |
| **10.853** | Decane, 3,7-dimethyl- | 3,7-dimethyldecane | 3 | 493694 | 98 | 4,4 |
| **10.992** | RT10.992 | - | 3 | 168073 | - | 1,81 |
| **11.198** | RT11.198 | - | 3 | 436765 | | 251,42 |
| **12.402** | Dodecane, 4,6-dimethyl- | 4,6-Dimethyldodecane | 3 | 467680 | 98 | 10,32 |
| **12.489** | Heptane, 2,2,4-trimethyl- | 2,2,4-trimethylheptane | 3 | 169666 | 97 | 6,68 |
| **12.651** | Tridecane, 6-methyl- | 6-methyltridecane | 3 | 184842 | 90 | 5,14 |
| **14.505** | RT14.505 | - | 3 | 311273 | - | 41,11 |
| **15.704** | 11-Methyldodecanol | Isotridecanol | 3 | 255654 | 99 | 17,39 |
| **15.898** | 1-Undecene, 7-methyl- | 7-Methyl-1-undecene | 3 | 357253 | 99 | 18,65 |
| **16.359** | Decane, 2,9-dimethyl- | 2,9-Dimethyldecane | 3 | 213057 | 96 | 3,8 |

| *Cookeina* sp. HS-SPME VCQ23 | | | | | | |
|---|---|---|---|---|---|---|
| **RT** | **Name** | **Common name** | **Identification level** | **Area** | **SI** | **S/N** |
| **18.326** | Caryophyllene | beta-caryophyllene | 3 | 116969 | 99 | 29,97 |
| **19.510** | RT19.510 | - | 3 | 391641 | - | 173,75 |

**Table S21.** Volatile organic compounds identified in *Cookeina* sp. using SHS-SPME-GC-MS in situ.

| *Cookeina* **sp. SHS-SPME VCQ23** | | | | | | |
|---|---|---|---|---|---|---|
| **RT** | **Name** | **Common name** | **Identification level** | **Area** | **SI** | **S/N** |
| **1.838** | n-Hexane | Hexane | 3 | 187844 | 99 | 131,61 |
| **3.480** | Toluene | Toluene | 3 | 55106 | 91 | 71,55 |
| **5.022** | Nonane, 4-ethyl-5-methyl- | 4-Ethyl-5-methylnonane | 3 | 24612 | 89 | 17,23 |
| **5.175** | RT5.175 | - | 3 | 404929 | - | 237,48 |
| **5.329** | Benzene, 1,3-dimethyl- | m-xylene | 3 | 641678 | 99 | 3,34 |
| **5.835** | o-Xylene | o-Xylene | 3 | 652225 | 98 | 0,88 |
| **6.188** | Oxime-, methoxy-phenyl-_ | methyl N-hydroxybenzenecarboximidate | 3 | 3258263 | 100 | 2008,12 |
| **6.541** | Benzene, (1-methylethyl)- | cumene | 3 | 24898 | 86 | 31,87 |
| **6.757** | (1R)-2,6,6-Trimethylbicyclo [3.1.1] hept-2-ene | alpha-pinene | 3 | 68853 | 97 | 68,13 |
| **7.107** | Camphene | Camphene | 3 | 31303 | 86 | 11,22 |
| **7.340** | RT7.340 | - | 3 | 313711 | - | 91,63 |
| **7.777** | . beta. -Pinene | beta-pinene | 3 | 53693 | 98 | 63,19 |
| **7.890** | RT7.890 | - | 3 | 159599 | - | 9,09 |
| **8.091** | Heptane, 2,2,4,6,6-pentamethyl- | 2,2,4,6,6-pentamethylheptane | 3 | 650708 | 99 | 92,95 |
| **8.946** | p-Cymene | p-Cymene | 3 | 762520 | 98 | 535,58 |
| **9.539** | Hexadecane | Hexadecane | 3 | 202610 | 96 | 1,56 |
| **10.791** | Undecane | Undecane | 3 | 3782213 | 99 | 118,65 |
| **11.225** | 5-Undecene, 9-methyl-, (Z)- | (Z)-9-methylundec-5-ene | 3 | 1358577 | 98 | 10,81 |
| **11.928** | Bicyclo[2.2.1]heptan-2-one, 1,7,7-trimethyl-, (1S)- | camphor | 3 | 1286535 | 96 | 59,17 |
| **12.409** | 2-Undecanethiol, 2-methyl- | 2-Methylundecane-2-thiol | 3 | 463462 | 96 | 3,49 |
| **12.512** | Undecane, 3-methyl- | 3-methylundecane | 3 | 773230 | 97 | 30,9 |
| **13.221** | Tridecane | Tridecane | 3 | 4004739 | 99 | 85,71 |
| **13.544** | Decane, 5-ethyl-5-methyl- | 5-ethyl-5-methyldecane | 3 | 762625 | 96 | 6,74 |
| **14.226** | Cyclopentane, hexyl- | hexylcyclopentane | 3 | 617118 | 96 | 7,97 |
| **14.596** | 1-Decanol, 2-hexyl- | 2-Hexyldecan-1-ol | 3 | 370235 | 98 | 4,63 |
| **15.686** | 1-Nonene, 4,6,8-trimethyl- | 4,6,8-trimethyl-1-nonene | 3 | 463147 | 95 | 7,74 |
| **16.354** | Heptadecane, 7-methyl- | 7-methylheptadecane | 3 | 252283 | 95 | 2,6 |
| **17.123** | Tridecane, 3-methyl- | 3-Methyltridecane | 3 | 533212 | 97 | 5,86 |
| **17.475** | RT17.475 | - | 3 | 650697 | - | 40,13 |

| *Cookeina* sp. SHS-SPME VCQ23 | | | | | | |
|---|---|---|---|---|---|---|
| **RT** | **Name** | **Common name** | **Identification level** | **Area** | **SI** | **S/N** |
| **18.804** | 2-Hexyl-1-octanol | 2-Hexyl-1-octanol | 3 | 164428 | 96 | 6,28 |

**Table S22.** Volatile organic compounds identified in *Trametes* sp. using HS-SPME-GC-MS under laboratory conditions.

| *Trametes* sp. HS - SPME VCQ27 | | | | | | |
|---|---|---|---|---|---|---|
| **RT** | **Name** | **Common name** | **Identification level** | **Area** | **SI** | **S/N** |
| **7.488** | Benzaldehyde | Benzaldehyde | 3 | 62935 | 83 | 20,72 |
| **7.887** | 1-Octen-3-ol | 1-Octen-3-ol | 3 | 2127537 | 99 | 1320,95 |
| **8.024** | 3-Octanone | 3-Octanone | 3 | 479173 | 99 | 266,15 |
| **8.265** | 3-Octanol | 3-Octanol | 3 | 265495 | 97 | 253,5 |
| **8.417** | Octanal | Octanal | 3 | 80367 | 93 | 0,72 |
| **9.079** | D-Limonene | D-Limonene | 3 | 91907 | 97 | 52,3 |
| **9.511** | RT9.511 | - | 3 | 32751 | - | 10,93 |
| **9.779** | 2-Octenal, (E)- | trans-2-Octenal | 3 | 375044 | 99 | 154,82 |
| **10.073** | 2-Decen-1-ol | trans-2-Decen-1-ol | 3 | 571025 | 97 | 197,04 |
| **20.186** | Butylated Hydroxytoluene | Butylhydroxytoluene | 3 | 119781 | 98 | 146,73 |
| **35.227** | Cannabidiol | Cannabidiol | 3 | 121565 | 97 | 98,75 |

**Table S23.** Volatile organic compounds identified in *Stereum sanguinolentum* using HS-SPME-GC-MS under laboratory conditions.

| *Stereum sanguinolentum* DHS-SPME VCQ28 | | | | | | |
|---|---|---|---|---|---|---|
| **RT** | **Name** | **Common name** | **Identification level** | **Area** | **SI** | **S/N** |
| **5.248** | Hexane, 2,3,4-trimethyl- | 2,3,4-trimethylhexane | 3 | 82917 | 92 | 21,8 |
| **5.385** | o-Xylene | o-xylene | 3 | 88691 | 91 | 3,5 |
| **6.798** | . alpha. -Pinene | alpha-pinene | 3 | 26758 | 95 | 25,9 |
| **7.445** | RT7.455 | - | 3 | 23854 | - | 30,07 |
| **8.974** | p-Cymene | p-Cymene | 3 | 79471 | 95 | 51,06 |
| **9.100** | RT9.100 | - | 3 | 106499 | - | 19,23 |
| **10.810** | Undecane | Undecane | 3 | 204240 | 94 | 12,92 |
| **11.945** | Camphor | Camphor | 3 | 54314 | 92 | 8,33 |
| **13.242** | Tridecane | Tridecane | 3 | 343581 | 97 | 42,45 |
| **17.824** | 1,2,4-Methenoazulene, decahydro-1,5,5,8a-tetramethyl-, [1S-(1. alpha.,2. alpha.,3a. beta.,4. alpha.,8a. beta.,9R*)]- | 1,2,4-Methenoazulene, decahydro-1,5,5,8a-tetramethyl- | 3 | 89905 | 98 | 18,61 |
| **17.968** | 2H-2,4a-Methanonaphthalene, 1,3,4,5,6,7-hexahydro-1,1,5,5-tetramethyl-, (2S)- | trans-Isolongifolene | 3 | 39551 | 95 | 11,51 |
| **18.053** | Longifolene | Longifolene | 3 | 91529 | 98 | 15,12 |
| **20.202** | Butylated Hydroxytoluene | 2,6-Di-tert-butyl-4-methylphenol | 3 | 582833 | 99 | 239,35 |
| **34.836** | Heneicosane | Heneicosane | 3 | 130272 | 97 | 27,96 |

**Table S24.** Volatile organic compounds identified in *Stereum sanguinolentum* using SHS-SPME-GC-MS in situ.

| *Stereum sanguinolentum* HS-SPME VCQ28 | | | | | | |
|---|---|---|---|---|---|---|
| **RT** | **Name** | **Common name** | **Identification level** | **Area** | **SI** | **S/N** |
| **3.828** | 1-Octene | 1-Octene | 3 | 358617 | 99 | 113,51 |
| **4.768** | 2-Pentanone, 4-hydroxy-4-methyl- | Diacetone alcohol | 3 | 386366 | 92 | 171,56 |
| **5.168** | Ethylbenzene | Ethylbenzene | 3 | 509955 | 99 | 296,31 |
| **5.329** | Benzene, 1,3-dimethyl- | m-xylene | 3 | 3254808 | 99 | 4,16 |
| **5.836** | RT5.836 | - | 3 | 1364911 | 99 | 0,81 |
| **6.197** | Oxime-, methoxy-phenyl-_ | methyl N-hydroxybenzenecarboximidate | 3 | 1783958 | 99 | 884,54 |
| **6.761** | . alpha. -Pinene | alpha-pinene | 3 | 92411 | 98 | 100,06 |
| **7.350** | RT7.350 | - | 3 | 420467 | - | 321,71 |
| **7.847** | 1-Octen-3-ol | 1-Octen-3-ol | 3 | 9907624 | 99 | 924,9 |
| **7.989** | 3-Octanone | 3-Octanone | 3 | 5751739 | 99 | 67,57 |
| **8.228** | 3-Octanol | 3-Octanol | 3 | 7848097 | 99 | 1442,16 |
| **8.497** | 2-Pentanol, propanoate | 2-Pentyl propionate | 3 | 606134 | 99 | 0,08 |
| **9.110** | Eucalyptol | Eucalyptol | 3 | 2256278 | 99 | 385,67 |
| **9.899** | Nonane, 3-methyl- | 3-methylnonane | 3 | 117924 | 96 | 6,07 |
| **10.205** | Decane, 3,4-dimethyl- | 3,4-dimethyldecane | 3 | 194115 | 93 | 2,43 |
| **10.518** | Heptane, 1,1'-oxybis- | Ether, diheptyl | 3 | 744421 | 99 | 18,66 |
| **10.796** | Undecane | Undecane | 3 | 212061 | 92 | 2,32 |
| **11.220** | RT11.215 | - | 3 | 509181 | - | 2,52 |
| **11.394** | Heptane, 4-ethyl-2,2,6,6-tetramethyl- | 4-ethyl-2,2,6,6-tetramethylheptane | 3 | 138323 | 96 | 4,96 |
| **12.403** | Dodecane, 4,6-dimethyl- | 4,6-Dimethyldodecane | 3 | 1031987 | 99 | 45,74 |
| **12.489** | Octane, 2,2-dimethyl- | 2,2-Dimethyloctane | 3 | 365841 | 98 | 22,31 |
| **13.555** | Dodecane, 2,6,10-trimethyl- | Farnesane | 3 | 43550 | 81 | 5,67 |
| **13.915** | RT13.915 | - | 3 | 145816 | 98 | 70,48 |
| **14.608** | Decane, 2-methyl- | 2-methyldecane | 3 | 122298 | 97 | 3,15 |
| **14.688** | Nonane, 3,7-dimethyl- | 3,7-dimethylnonane | 3 | 142239 | 88 | 3,49 |
| **14.844** | RT14.844 | - | 3 | 166395 | 83 | 2,85 |
| **16.444** | Decane, 3,7-dimethyl- | 3,7-dimethyldecane | 3 | 47341 | 94 | 1,37 |
| **16.550** | Valerena-4,7(11)-diene | valerena-4,7(11)-diene | 3 | 86113 | 98 | 17,29 |
| **16.920** | Tetradecane, 2,5-dimethyl- | 2,5-dimethyltetradecane | 3 | 61859 | 83 | 3,02 |
| **17.493** | Hexane, 4-ethyl-2-methyl- | 4-Ethyl-2-methylhexane | 3 | 738976 | 99 | 28,62 |

| *Stereum sanguinolentum* HS-SPME VCQ28 | | | | | | |
|---|---|---|---|---|---|---|
| **RT** | **Name** | **Common name** | **Identification level** | **Area** | **SI** | **S/N** |
| **18.856** | (3aR,4R,8R,8aS)-3a,4,8a-Trimethyl-7-methylenedecahydro-4,8-methanoazulene-rel- | Gymnomitrene | 3 | 118692 | 97 | 17,95 |
| **19.252** | 2,5-Cyclohexadiene-1,4-dione, 2,6-bis(1,1-dimethylethyl)- | 2,6-Di-tert-butyl-P-benzoquinone | 3 | 336012 | 100 | 39,17 |
| **20.193** | Butylated Hydroxytoluene | 2,6-Di-tert-butyl-4-methylphenol | 3 | 12804553 | 100 | 2617,23 |
| **23.210** | RT23.210 | - | 3 | 215535 | - | 83,14 |

**Table S25.** Volatile organic compounds identified in *Stereum sanguinolentum* using DHS-SPME-GC-MS in situ.

| *Stereum sanguinolentum* SHS-SPME VCQ28 | | | | | | |
|---|---|---|---|---|---|---|
| **RT** | **Name** | **Common name** | **Identification level** | **Area** | **SI** | **S/N** |
| **1.853** | n-Hexane | Hexane | 3 | 137069 | 98 | 99,69 |
| **5.180** | RT5.180 | - | 3 | 338644 | - | 285,52 |
| **5.334** | o-Xylene | o-Xylene | 3 | 590996 | 99 | 2,49 |
| **5.838** | p-Xylene | p-Xylene | 3 | 618966 | 96 | 0,73 |
| **6.185** | Oxime-, methoxy-phenyl-_ | methyl N-hydroxybenzenecarboximidate | 3 | 2202409 | 100 | 836,55 |
| **6.760** | . alpha. -Pinene | alpha-pinene | 3 | 114455 | 99 | 102,96 |
| **7.340** | RT7.340 | - | 3 | 266238 | - | 414,44 |
| **7.859** | 1-Octen-3-ol | 1-Octen-3-ol | 3 | 654056 | 96 | 46,98 |
| **8.094** | Heptane, 2,2,4,6,6-pentamethyl- | 2,2,4,6,6-pentamethylheptane | 3 | 827046 | 99 | 141,83 |
| **8.947** | p-Cymene | p-Cymene | 3 | 724371 | 99 | 655,2 |
| **9.797** | . gamma. -Terpinene | gamma-terpinene | 3 | 526004 | 92 | 150,18 |
| **10.791** | Undecane | Undecane | 3 | 3411838 | 99 | 91,4 |
| **11.095** | RT11.095 | - | 3 | 269768 | - | 114,45 |
| **11.215** | RT11.215 | - | 3 | 1102019 | - | 7,99 |
| **11.928** | Bicyclo[2.2.1]heptan-2-one, 1,7,7-trimethyl-, (1S)- | camphor | 3 | 1023279 | 98 | 56,12 |
| **12.412** | 1-Heptanol, 2,4-diethyl- | 2,4-Diethylheptan-1-ol | 3 | 206480 | 92 | 2,76 |
| **12.512** | Undecane, 3-methyl- | 3-methylundecane | 3 | 602420 | 99 | 17,34 |
| **12.875** | Undecane, 3-methylene- | 2-Ethyl-1-decene | 3 | 161853 | 97 | 6,67 |
| **13.222** | Tridecane | Tridecane | 3 | 3967798 | 99 | 85,02 |
| **13.544** | Decane, 5-ethyl-5-methyl- | 5-ethyl-5-methyldecane | 3 | 408696 | 97 | 4,92 |
| **14.225** | Cyclopentane, pentyl- | Pentylcyclopentane | 3 | 459023 | 98 | 8,94 |
| **14.595** | 1-Decanol, 2-hexyl- | 2-Hexyl-1-decanol | 3 | 233170 | 97 | 4,29 |
| **15.686** | 1-Nonene, 4,6,8-trimethyl- | 4,6,8-trimethyl-1-nonene | 3 | 193182 | 96 | 7,68 |
| **17.123** | Tridecane, 3-methyl- | 3-Methyltridecane | 3 | 611565 | 98 | 7,35 |
| **21.858** | 2,2,4-Trimethyl-1,3-pentanediol diisobutyrate | Texanol Isobutyrate | 3 | 194891 | 98 | 85,98 |
| **37.837** | Tetratetracontane | Tetratetracontane | 3 | 23439870 | 100 | 3490,2 |

**Table S26.** Volatile organic compounds identified in *Panus similis* using DHS-SPME-GC-MS in situ.

| *Panus similis* DHS-SPME VCQ30 | | | | | | |
|---|---|---|---|---|---|---|
| **RT** | **Name** | **Common name** | **Identification level** | **Area** | **SI** | **S/N** |
| **7.329** | 4,4-Dimethylpent-2-enal | 4,4-Dimethylpent-2-enal | 3 | 172581 | 96 | 6,47 |
| **7.886** | RT7.886 | - | 3 | 33629 | 83 | 18,75 |
| **8.015** | 3-Octanone | 3-Octanone | 3 | 142728 | 94 | 77,99 |
| **8.256** | 3-Octanol | 3-Octanol | 3 | 110544 | 86 | 55,24 |
| **9.063** | D-Limonene | D-Limonene | 3 | 274939 | 98 | 87,53 |
| **10.826** | Linalool | Linalool | 3 | 25612 | 83 | 9,64 |
| **12.172** | RT12.172 | - | 3 | 27736 | - | 47,86 |
| **17.488** | Hexane, 4-ethyl-2-methyl- | 4-Ethyl-2-methylhexane | 3 | 55438 | 97 | 68,3 |
| **20.188** | Butylated Hydroxytoluene | 2,6-Di-tert-butyl-4-methylphenol | 3 | 1097567 | 100 | 850,68 |

**Table S27.** Volatile organic compounds identified in *Panus similis* using SHS-SPME-GC-MS in situ.

| *Panus similis* SHS-SPME VCQ30 | | | | | | |
|---|---|---|---|---|---|---|
| **RT** | **Name** | **Common name** | **Identification level** | **Area** | **SI** | **S/N** |
| **3.728** | 1-Hexanol | 1-Hexanol | 3 | 92221 | 85 | 31,42 |
| **5.194** | 1-Hexene, 3,5,5-trimethyl- | 3,5,5-Trimethyl-1-hexene | 3 | 91894 | 94 | 85,63 |
| **5.284** | 3-Octanone | 3-Octanone | 3 | 250462 | 96 | 163,13 |
| **5.952** | D-Limonene | D-Limonene | 3 | 379874 | 97 | 131,02 |
| **12.661** | Butylated Hydroxytoluene | 2,6-Di-tert-butyl-4-methylphenol | 3 | 402981 | 99 | 449,35 |

**Table S28.** Volatile organic compounds identified in *Panus similis* using HS-SPME-GC-MS under laboratory conditions.

| *Panus similis* HS-SPME VCQ30 | | | | | | |
|---|---|---|---|---|---|---|
| **RT** | **Name** | **Common name** | **Identification level** | **Area** | **SI** | **S/N** |
| **7.445** | Benzaldehyde | Benzaldehyde | 3 | 237194 | 97 | 75,85 |
| **8.040** | 1-Octen-3-ol | 1-Octen-3-ol | 3 | 13259478 | 97 | 6184,77 |
| **8.133** | 3-Octanone | 3-Octanone | 3 | 3929427 | 99 | 6578,86 |
| **8.348** | 3-Octanol | 3-Octanol | 3 | 3948227 | 99 | 940,07 |
| **8.450** | Octanal | Octanal | 3 | 312671 | 99 | 1,36 |
| **9.092** | D-Limonene | D-Limonene | 3 | 2240465 | 100 | 1249,74 |
| **9.441** | RT9.441 | - | 3 | 388097 | - | 219,69 |
| **9.775** | 2-Octenal, (E)- | trans-2-Octenal | 3 | 819257 | 100 | 615,41 |
| **10.124** | 1-Octanol | 1-Octanol | 3 | 1022323 | 99 | 749,68 |
| **10.863** | Linalool | Linalool | 3 | 2123954 | 99 | 709,65 |
| **11.195** | Phenylethyl Alcohol | 2-phenylethanol | 3 | 258138 | 97 | 151,59 |
| **15.039** | Oxirane, (7-octenyl)- | 1,2-Epoxy-9-decene | 3 | 151666 | 99 | 52,88 |
| **15.150** | 1-Tetradecyne | 1-Tetradecyne | 3 | 69335 | 89 | 17,35 |
| **17.439** | 3-Isopropyl-6,8a-dimethyl-1,2,4,5,8,8a-hexahydroazulene | Daucene | 3 | 611743 | 100 | 317,8 |
| **18.461** | (3S,3aS,8aR)-6,8a-Dimethyl-3-(prop-1-en-2-yl)-1,2,3,3a,4,5,8,8a-octahydroazulene | Isodaucene | 3 | 372168 | 100 | 62,09 |
| **19.308** | (1R,4R,5S)-1,8-Dimethyl-4-(prop-1-en-2-yl) spiro [4.5] dec-7-ene | 4-epi-alpha-acoradiene | 3 | 48766 | 97 | 13,74 |
| **19.979** | cis-. alpha. -Bisabolene | cis-alpha-Bisabolene | 3 | 398345 | 100 | 32,96 |
| **20.185** | Butylated Hydroxytoluene | 2,6-Di-tert-butyl-4-methylphenol | 3 | 396132 | 95 | 271,86 |
| **20.602** | (3aS,8aS)-6,8a-Dimethyl-3-(propan-2-ylidene)-1,2,3,3a,4,5,8,8a-octahydroazulene | Dauca-4(11),8-diene | 3 | 1164124 | 100 | 701,86 |

| *Panus similis* HS-SPME VCQ30 | | | | | | |
|---|---|---|---|---|---|---|
| **RT** | **Name** | **Common name** | **Identification level** | **Area** | **SI** | **S/N** |
| **20.785** | Cyclohexene, 4-[(1E)-1,5-dimethyl-1,4-hexadien-1-yl]-1-methyl- | trans-alpha-bisabolene | 3 | 70510 | 95 | 49,52 |
| **34.752** | Heneicosane | Heneicosane | 3 | 278093 | 100 | 25,95 |
